# Vertical stratification of the air microbiome in the lower troposphere

**DOI:** 10.1073/pnas.2117293119

**Published:** 2022-02-07

**Authors:** Daniela I. Drautz-Moses, Irvan Luhung, Elena S. Gusareva, Carmon Kee, Nicolas E. Gaultier, Balakrishnan N. V. Premkrishnan, Choou Fook Lee, See Ting Leong, Changsook Park, Zhei Hwee Yap, Cassie E. Heinle, Kenny J. X. Lau, Rikky W. Purbojati, Serene B. Y. Lim, Yee Hui Lim, Shruti Ketan Kutmutia, Ngu War Aung, Elaine L. Oliveira, Soo Guek Ng, Justine Dacanay, Poh Nee Ang, Samuel D. Spence, Wen Jia Phung, Anthony Wong, Ryan J. Kennedy, Namrata Kalsi, Santhi Puramadathil Sasi, Lakshmi Chandrasekaran, Akira Uchida, Ana Carolina M. Junqueira, Hie Lim Kim, Rudolf Hankers, Thomas Feuerle, Ulrich Corsmeier, Stephan C. Schuster

**Affiliations:** ^a^Singapore Centre for Environmental Life Sciences Engineering, Nanyang Technological University, Singapore 637551;; ^b^The Asian School of the Environment, Nanyang Technological University, Singapore 637459;; ^c^Departamento de Genética, Instituto de Biologia, Universidade Federal do Rio de Janeiro, Rio de Janeiro 21941-590, Brazil;; ^d^Institute of Flight Guidance, Technische Universität, 38108 Braunschweig, Germany;; ^e^Institute of Meteorology and Climate Research, Karlsruhe Institute of Technology, 76021 Karlsruhe, Germany

**Keywords:** microbial ecology, bioaerosols, air microbiome, atmospheric turbulence, microbial dispersal

## Abstract

The troposphere constitutes the final frontier of global ecosystem research due to technical challenges arising from its size, low biomass, and gaseous state. Using a vertical testing array comprising a meteorological tower and a research aircraft, we conducted synchronized measurements of meteorological parameters and airborne biomass (*n* = 480) in the vertical air column up to 3,500 m. The taxonomic analysis of metagenomic data revealed differing patterns of airborne microbial community composition with respect to time of day and height above ground. The temporal and spatial resolution of our study demonstrated that the diel cycle of airborne microorganisms is a ground-based phenomenon that is entirely absent at heights >1,000 m. In an integrated analysis combining meteorological and biological data, we demonstrate that atmospheric turbulence, identified by potential temperature and high-frequency three-component wind measurements, is the key driver of bioaerosol dynamics in the lower troposphere. Multivariate regression analysis shows that at least 50% of identified airborne microbial taxa (*n* = ∼10,000) are associated with either ground or height, allowing for an understanding of dispersal patterns of microbial taxa in the vertical air column. Due to the interconnectedness of atmospheric turbulence and temperature, the dynamics of microbial dispersal are likely to be impacted by rising global temperatures, thereby also affecting ecosystems on the planetary surface.

## Significance

Large-scale meteorological and biological data demonstrate the vertical stratification of airborne biomass. The previously described diel cycle of airborne microorganisms is shown to disappear at height. Atmospheric turbulence and stratification are shown to be defining factors for the scale and boundaries, dynamics, and natural variability of airborne biomass, resulting in the biological organization of the planetary air ecosystem. The atmosphere above the mixing layer height is proposed to act as a sink for microorganisms. With atmospheric processes being temperature dependent, rising global temperatures will result in major disruptions of the currently observed airborne microbial community structures. Increased abundances of radio-tolerant bacteria at height will allow investigation of these microorganisms’ life cycle in the planetary atmosphere.

The scale and boundaries of microbial biomass in the earth’s atmosphere remain elusive despite the advances made for the understanding of microbial community composition of terrestrial and aquatic ecosystems (1, 2). This is despite a century of innovative research into the processes that disperse bacteria, fungi, and pollen in the atmosphere. Early interest in airborne microbial organisms is attributed to the works and writing of Louis Pasteur in 1865 (3), with subsequent assessments of airborne microorganisms relying on microscopic and cultivation-based methods (4, 5). Attempts to analyze the microbial content of the vertical air column date back to the early 20th century when aviation pioneers such as Charles Lindberg conducted the Skyhook experiments (6–10). Other vertical experimental designs resorted to air sampling on mountains with high elevation (11–13), low-height sampling at research towers (14), or meteorological balloons (15). In parallel to addressing the issues associated with vertical sampling, more recent studies have advanced the field taxonomically with molecular biological approaches, while still being challenged by limiting amounts of airborne biomass (16, 17). Furthermore, the recently discovered diel cycle of airborne microbial communities (18) mandates synchronized, time-resolved experimental approaches with short sampling times, requiring an array of high-volumetric air samplers. In this study, we have addressed the aforementioned issues through the combination of a meteorological tower and a research aircraft, fitted with a total of 38 high-volumetric air samplers. The newly designed vertical testing array allowed for simultaneous sampling of bioaerosols at a large spatial scale, while maintaining high temporal and taxonomic resolution. The combination of aviation, physicochemical measurements, advances in metagenomics, and air sampling techniques resulted in a high-resolution vertical map of airborne microorganisms in the lower troposphere. The previously discovered diel cycle of airborne microorganisms is now shown to be a ground-based phenomenon that diminishes with height. Our analysis identified diurnal atmospheric turbulence and atmospheric stability as the key factors for the dispersal of ground-based microorganisms and the transport of biomass from higher air layers to the ground. Among the investigated physicochemical factors, temperature was identified as the main driver for atmospheric dynamics. Given the role of air temperature in regard to the abundance and diversity of airborne microorganisms, investigating the impact of global warming on atmospheric ecosystems is warranted, also in light of emerging novel airborne diseases (19).

## Results

### Vertical Testing Array.

**Fig. 1.**
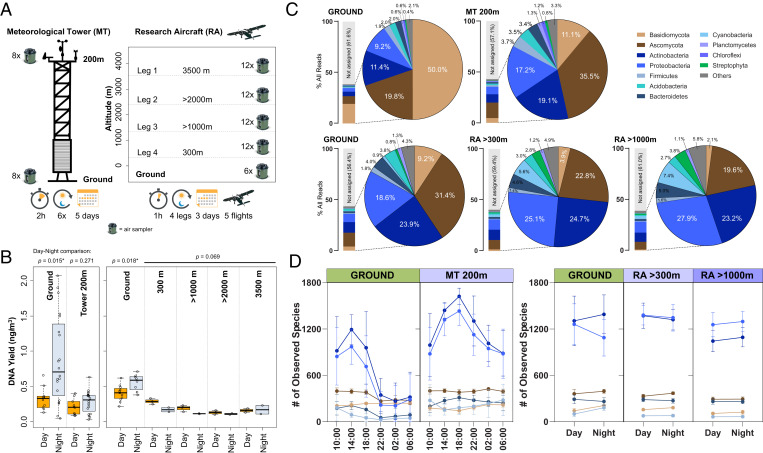
Vertical testing array and airborne microbial communities at different heights. (*A*) Air sampling campaigns conducted at the MT (*Left*) and with a RA (*Right*). (*B*) Total airborne DNA concentration per volume of air (ng/m^3^) at different sampling heights from the MT (*Left* box) and RA (*Right* box). The air samples were grouped based on day (orange) or night (gray) sampling. The lines represent the median values, while the error bars denote SD among replicates. (*C*) Metagenomic composition of air samples at different sampling heights (MT *Top*, RA *Bottom*). The bars highlight the portion (% all reads) of DNA assignable at phylum level. Pie charts indicate % of assigned reads for the 10 most abundant phyla. (*D*) Diversity of airborne taxa at different altitudes and time of day (MT *Left*, RA *Right*) for each phylum. The dots are mean values, while the error bars denote SD among replicates.

To enable the collection of metagenomics samples from vertical air columns, multiple spatial settings were evaluated. Sites that do not provide unrestricted airflow, such as high-rise buildings and mountain tops, were found to be unsuitable as they obstruct the natural airflow, resulting in highly similar taxonomic profiles at different heights (20) (*SI Appendix*, sections 11 and 12). An ideal experimental setup was found in the combination of a 200-m meteorological tower (MT) (Karlsruhe Institute of Technology [KIT], Institute of Meteorology and Climate Research, Karlsruhe, Germany) and a research aircraft (RA) (Technische Universität Braunschweig, Institute of Flight Guidance, Braunschweig, Germany), which were both fitted with an extensive set of meteorological sensors (e.g., temperature, absolute humidity, solar radiation, and wind speed) (Fig. 1*A*). In the instance of the MT, an automated sampling system was devised that allowed simultaneous air sampling at different heights and time points without human supervision. This resulted in an environmental time series with high temporal resolution (MT, ground and 200 m, 120 samples over 5 consecutive days). In the instance of RA sampling at a large spatial scale (0 to 3,500 m, 40-km triangle), human supervision for air sampling was required (five 4-h flights, 48 in-flight samples per flight, 12 samples per height, 24 ground samples, 360 total for the RA campaign). Twelve air samplers were rack mounted inside the aircraft cabin and provided with a stream of outside air that exceeded the samplers’ airflow rate (300 L/min/sampler) by five- to sixfold (*SI Appendix*, Fig. S34 and Table S3). MT and RA experimental setups independently resulted in highly reproducible metagenomic profiles of the air samples taken. This enabled us to investigate airborne microbial communities relative to atmospheric stability in day/night settings and at various heights. By coordinating sampling times between MT and RA, datasets with both high temporal (six time points per 24 h) and large spatial resolution (0 to 3,500 m, 40-km triangle) were obtained.

### Abundance and Diversity of Airborne Microbial Communities in the Vertical Air Column.

Extracted DNA concentrations were used as a proxy for bioaerosol amassment as previously described (17, 18). As a result, we show that recorded DNA amounts vary with time of day and height. It is therefore important to investigate any height-related changes in a time-specific manner. In detail, a 400% difference in DNA abundance was observed between day and night samples in the instance of the MT experiment (range, 0.2 to 2 ng/m^3^), whereas a much smaller diel difference was observed for the large spatial-scale RA experiment (0.2 to 0.6 ng/m^3^). The decline in DNA concentration in the vertical air column was independently confirmed by both sampling settings, MT and RA, based on 480 samples (Fig. 1*B* and *SI Appendix*, Figs. S30 and S37). Metagenomic sequencing of the extracted DNA resulted in a taxonomic classification (17) (*SI Appendix*, Figs. S18–S28 and S40–S62) with respect to altitude, as shown in Fig. 1*C*. Of the 10 most abundant phyla in the MT experiment, Basidiomycota showed the largest reduction in relative abundance with height (38.9%), while Ascomycota was found to increase by 15.7% at the tower top. Actino- and Proteobacteria as well as Firmicutes similarly showed a moderate increase with height (2 to 8%). The large spatial-scale experiment (RA) confirmed the same taxonomic patterns for Basidiomycota and bacterial phyla. However, relative abundance of Ascomycota was found to decrease at higher altitudes. These differences in Basidiomycota abundance are attributed to 1) different land types at the two sampling sites (MT, forested; RA, grass land), and 2) a difference in spatial scale of the two experiments. The fraction of unassignable DNA reads in both the MT and RA ranged from 56 to 61% (Fig. 1*C*), dependent on current versions of public sequence databases (18). The taxonomic diversity of the air samples was assessed by computing the Chao-1 index for each respective height. In the case of the MT, the highest diversity was observed for the two taxonomic groups Actino- and Proteobacteria at 14:00 h at ground level, while a similar increase in diversity was recorded 4 h later at 200-m height (Fig. 1*D*). This finding was validated in the RA experiment with Actino- and Proteobacteria being the most diverse taxonomic groups at all sampling heights (Fig. 1*D*).

### The Diel Cycle of Airborne Microorganisms Decreases with Height.

**Fig. 2.**
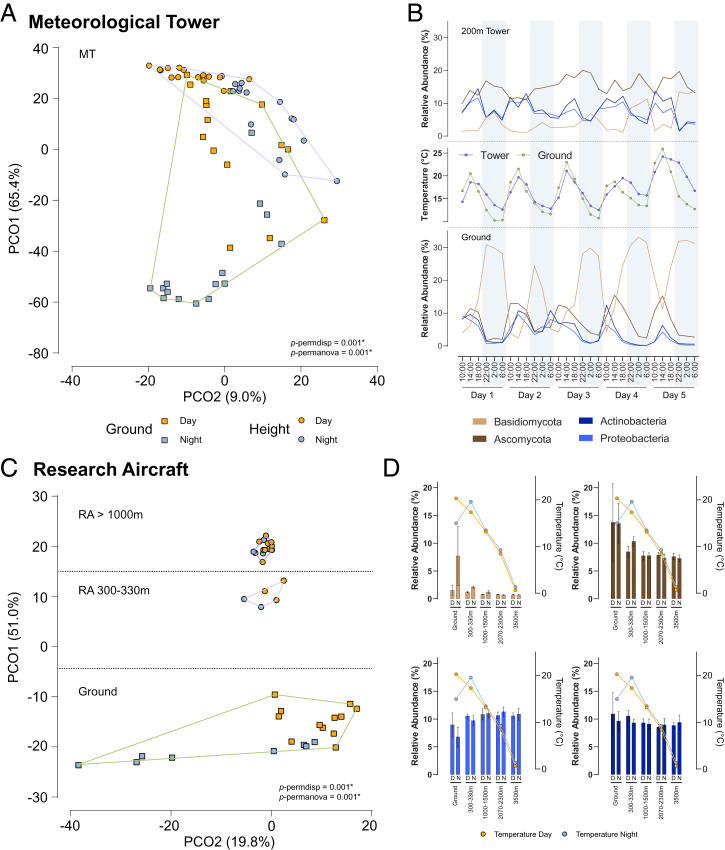
Diel cycle of airborne microbial communities at different altitudes. (*A*) Principal coordinate analysis (Bray–Curtis dissimilarity, species level) between MT air samples collected at different times of day and altitudes. Circles represent 200-m tower-top samples and the squares, ground-level samples. Orange color depicts day samples, while gray color represents night samples. (*B*) Time series of relative abundances of the top four most abundant phyla from the MT experiment. Nighttime periods are shaded in gray. Temperature (°C) profiles at the two sampling heights (ground, green; 200 m, blue) are shown in the *Center*. (*C*) Principal coordinate analysis (Bray–Curtis dissimilarity, species level) between RA air samples collected at different times of day and altitudes. Ground-level air samples are indicated as squares, while air samples collected during flights (300 to 3,500 m) are shown as circles. Daytime samples are colored in orange, and the nighttime samples are colored in gray. (*D*) Diel fluctuations in relative abundances of the top four most abundant phyla in the RA experiments. The bars denote the mean values, whereas the error bars are SDs. Temperature profiles (°C) at different heights and times of day are overlayed on the bars by the two colored lines (daytime [D], orange; nighttime [N], gray).

Clustering of sample data in the first two principal coordinates (PCos), based on Bray–Curtis dissimilarity, revealed significantly larger taxonomic variation for airborne microbial communities at the ground level compared to the tower top (200 m) (Fig. 2*A*). As previously reported for the tropical climate (18), our high temporal resolution analysis indicates that the microbial community compositions also alternate in temperate climate settings between day and night (*SI Appendix*, Fig. S18). In detail, the strict diel pattern of Basidiomycota, as well as the daytime abundance of Ascomycota and bacterial taxa at the ground, are beginning to be disrupted at a 200-m height (Fig. 2*B*). This observation becomes apparent in the small spatial scale of the MT experiment, which results in partially overlapping microbial communities in the PCo analysis. In contrast, the larger spatial scale of the RA experiment results in a complete separation of ground- and height-based communities. The scale of this experiment reveals that the diel cycle of airborne microbial organisms diminishes with height and is entirely absent at greater heights (1,000 to 3,500 m) (Fig. 2*C*). Heights of 200 to 300 m (MT and RA) were found to be in close enough proximity to the ground to contain components of the ground-based diel cycle. Importantly, at heights of 1,000 to 3,500 m, physicochemical parameters remain constant, as shown by the day/night temperature convergence, resulting in highly similar taxonomic profiles for all identified airborne microbial taxa (Fig. 2*D*). Temperature is therefore the key environmental parameter that drives the diel cycle of airborne microorganisms, as no other measured meteorological factor was significant. In fact, both experiments show that during the day, the air temperature near ground level is higher than at greater altitudes, whereas during nighttime, the air temperature profile is reversed, resulting in higher temperature at heights up to 300 m as compared to ground (Fig. 2*B* and *D*). This is reflected in slightly higher abundances of fungal taxa at heights of 200 to 300 m at night.

### Diurnal Atmospheric Turbulence as a Driver of Atmospheric Microbial Dispersal.

**Fig. 3.**
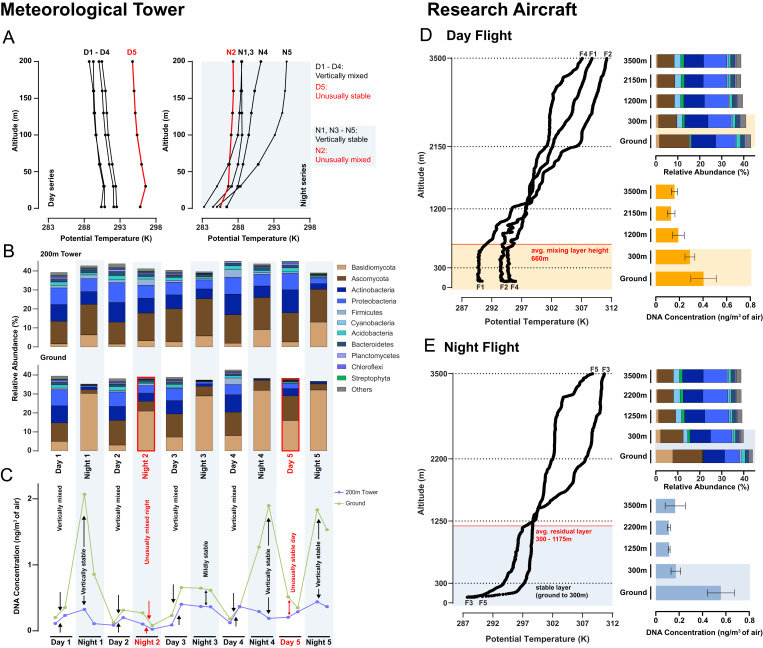
Atmospheric turbulence drives bioaerosol stratification in the vertical air column. (*A*) PT profiles for the five sampling days in the MT experiment segregated into daytime series (*Left*) and nighttime series (*Right*). Samples with mixed period of sunlight were excluded from analysis (6 AM and 6 PM). PT profiles that indicate unusual day or nighttime atmospheric stability are highlighted in red. (*B*) Averaged relative abundance (%) of airborne microbial communities for the five day and night samples of the MT experiment. Day and night samples with unusual atmospheric stabilities are highlighted in red. (*C*) Absolute abundance (total DNA concentration) profile of the ground (green) and 200 m tower-top (blue) air samples for the MT experiment. The state of stability (based on the PT profiles) is annotated according to the sampling time. (*D*) Averaged PT at various altitudes (*Left*) and airborne microbial community profiles (*Top Right*: relative abundance; *Bottom Right*: DNA concentration) of ground and flight air samples that were collected during daytime and (*E*) nighttime in the RA experiment. The estimated MLH is highlighted in orange for day flights, while the stable and residual layers formed at night are highlighted in gray.

Diurnal dynamic and thermodynamic processes cause turbulence due to wind shear and/or buoyant convection of air masses located between the ground and the top of the mixing layer (ranging from 500 to 1,500 m). One measure that can indicate atmospheric turbulence when high-frequency wind measurements are not available is potential temperature (PT). When PT is constant or decreasing with altitude, vertical mixing is likely to occur. Inversely, increasing values of PT result in vertically stable, stratified air masses (21). This is illustrated in the MT experiment, where daytime measurements show decreasing PT values with height, indicating strong vertical convection during this time period (Fig. 3 *A*, *Left*). In contrast, during the night, PT values increase with height, indicating vertically stable air masses (Fig. 3 *A*, *Right*). Exceptions to this pattern were observed for day 5 (partly stable stratification) and night 2 (partly unstable stratification). Our data show that metagenomic profiles (Fig. 3*B*) and absolute abundances (Fig. 3*C*) of airborne biomass in height-stratified air layers can be matched to the established meteorological observations. This is illustrated by near-identical taxonomic profiles (Fig. 3*B*) and similar levels of DNA concentration (converging arrows, Fig. 3*C*) at the ground and tower top when the vertical air masses are mixed. In contrast, when air masses are vertically stable, taxonomic profiles become distinct for ground and tower top (Fig. 3*B*), which is also supported by the differing DNA measurements between the two heights (opposing arrows, Fig. 3*C*). PT and horizontal wind speed measurements (as a proxy for vertical wind speed data) independently confirm the above-described atmospheric states of our MT environmental time series experiments (*SI Appendix*, Fig. S29).

Using the large spatial scale of the RA experiment (up to 3,500 m, 63 to 67 m/s air speed, 40-km triangle), mixing layer height (MLH) and stable residual layer (SRL) height were determined. The sensors of the RA allowed for direct measurements of the vertical wind speed, which were combined with the PT calculations of the height-dependent temperature measurements (Fig. 3 *D* and *E* and *SI Appendix*, Fig. S39 and Table S4). During the day flights, an average MLH of 660 m was imputed (Fig. 3*D*). No major differences were found below and above the MLH, with the exception of an increase of Basidiomycota and decrease of Firmicutes at ground level during daytime. During nighttime, a SRL was formed up to a height of 1,175 m, with a strongly stratified stable layer of up to 150-m height above the ground. This results in contrasting taxonomic and abundance profiles for the ground layer samples and to a lesser degree for the samples of up to 300-m height. The atmospheric conditions encountered above the boundary layer, therefore, explain the observed lack of diel dynamics of airborne microbial communities at greater height.

### Height- and Ground-Associated Airborne Microorganisms.

**Fig. 4.**
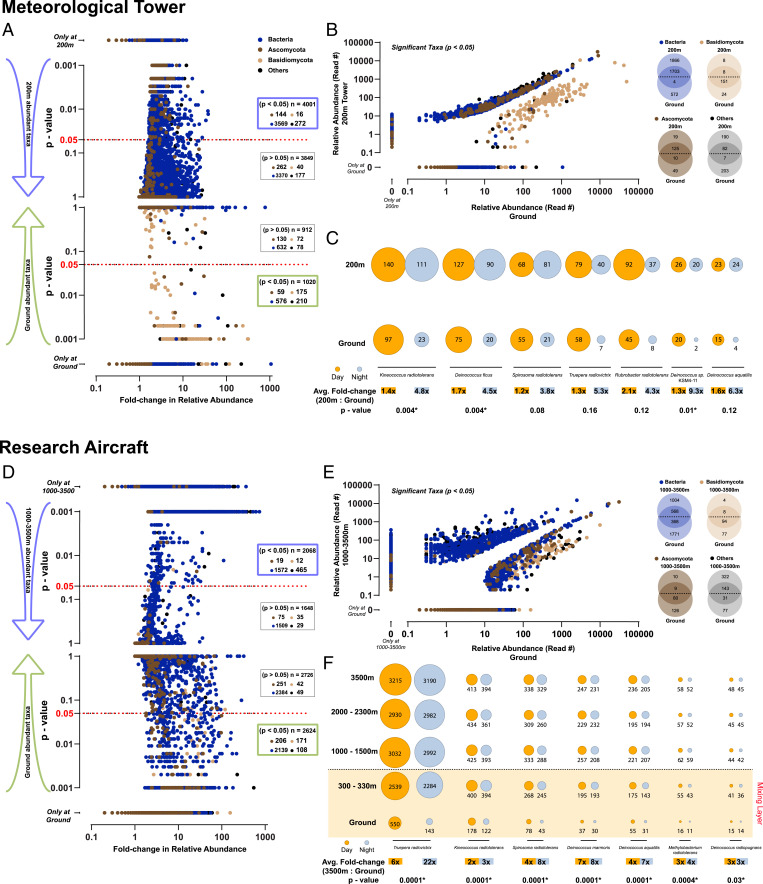
Association of airborne microorganisms to different altitudes in the lower troposphere. (*A* and *D*) Volcano graph plotting the mean fold change in relative abundance of specific species (*x* axis) and its corresponding *P* value difference (manyglm method, *y* axis) (*A*) between ground and 200-m tower-top air samples in the MT experiment and (*D*) between ground and 1,000- to 3,500-m air samples in the RA experiment. Each species is color labeled based on their domain/phylum. Species with significantly higher abundance at the 200-m tower top or 1,000- to 3,500-m height are plotted in the *Top* graph, while species with significantly higher abundance in the ground-level air are shown in the *Bottom* graph. Species with *P* value <0.05 are considered to be significantly associated with height. (*B* and *E*) Taxa cloud graph plotting the mean relative abundance of the species identified (*B*) in the MT experiment at ground level (*x* axis) and in the 200-m tower-top air layer (*y* axis) and (*E*) in the RA experiment on the ground level (*x* axis) and in the 1,000- to 3,500-m air layer (*y* axis). Only species significantly diverging between ground and high altitude are plotted (*P* < 0.05, manyglm method). The Venn diagrams denote the number of species that overlap between ground and high-altitude air samples in each group (domain/phylum/others). (*C* and *F*) Bubble charts showing the change in relative proportion of the top seven most abundant radio-tolerant bacterial species at different sampling times/altitudes in (*C*) the MT experiment and (*F*) the RA experiment. Day abundances are colored orange, while night abundances are in gray. The fold change and the corresponding *P* value (manyglm method) for the chosen taxa are listed at the *Bottom*.

Using the established measurements for MLH and nighttime SRL height, we investigated potential associations of identified airborne microorganisms to either ground or height. Despite the height of the tower (200 m) not exceeding the MLH, we can show that height-associated microbial diversity is larger than those of ground-level air masses (Fig. 4*A*). In our multivariate regression analysis, we calculated association of species abundance with height and plotted *P* values against the fold change of relative abundance. The analysis demonstrates a significant association (*P* < 0.05) of a large number of bacterial (*n* = 3,569 species) and Ascomycota (*n* = 144 species) taxa to height, in contrast to Basidiomycota (*n* = 16 species) (Fig. 4*A*, blue box). The respective ground-associated numbers are: Bacteria (*n* = 576 species), Ascomycota (*n* = 59 species), and Basidiomycota (*n* = 175 species) (Fig. 4*A*, green box). Considering only taxa significantly associated with height, a graph plotting abundances relative to ground and height visualizes the dispersal of bacteria, Ascomycota, and Basidiomycota in the respective air layers (Fig. 4*B*). The observed differences between the ground- and height-associated communities are further amplified by the larger spatial scale of the RA experiment, in which ground-level samples were compared to samples from heights of 1,000 m and above (Fig. 4*D*). In this experiment (Fig. 4*D*, blue box), heights above 1,000 m were largely dominated by bacterial taxa (*n* = 1,572), while Ascomycota taxa were much less frequent (*n* = 19). The RA ground-level air layers (Fig. 4*D*, green box) showed similar ratios of Ascomycota (*n* = 206) and bacterial (*n* = 2,139) taxa with a location-specific reduction in Basidiomycota (*n* = 108). As in the MT, the ratio of Basidiomycota taxa between ground and higher altitudes was 10-fold, indicative of being strictly associated with the ground layer (sky, *n* = 12; ground, *n* = 108), albeit at a lesser abundance compared to the MT experiment (Fig. 4 *D* and *E*).

### Abundance of Radio-Tolerant Microorganisms in Higher Air Layers.

The above-described dispersal and stratification patterns of specific microbiota in the vertical air column can be shown in detail using species-level taxonomic resolution. A key finding of our analysis is the observation that bacterial species, which have previously been described to be radio tolerant, are significantly more abundant at height (Fig. 4 *C* and *F*) (22). Specifically, the observed nighttime difference for the most abundant radio-tolerant taxon, *Kineococcus radiotolerans*, was found to be 4.8-fold between ground and tower top (200 m), whereas during daytime, the difference was only 1.4-fold in the MT experiment. This dispersal pattern is also observed for other radio-tolerant bacterial species (Fig. 4*C*). In the large spatial-scale RA experiment (Fig. 4*F*), the finding was independently confirmed for observations from a similar height range between ground-level and heights of 300 to 330 m. For heights above 1,000 m, this finding is even more pronounced with nighttime differences between ground and 3,500 m, resulting in a 22.3-fold change for *Truepera radiovictrix*. Corresponding daytime changes show a 5.8-fold increase, indicative of more pronounced daytime mixing and downward transport. No significant differences for day/nighttime were observed for any of the investigated radio-tolerant bacteria taxa in a range of 1,000 to 3,500 m. The spatial-temporal distribution of radio-tolerant airborne bacterial organisms, therefore, make them suitable proxies for studying the dispersal of atmospheric biomass above and below the mixing layer.

## Discussion

An innovative experimental design for investigating the stratification and microbial abundance of the vertical air column was developed by combining meteorological measurements with species-level metagenomic sequencing. Consisting of a MT and RA, the use of a vertical testing array resulted in the integration of temporally and spatially resolved physicochemical and biological data. This allowed interrogation of the potential stratification of airborne biomass in the near-surface atmosphere with a temporal resolution of 4 h and 200-m height at a fixed location in the instance of the MT and up to 3,500-m height, within a horizontal area of a 40-km triangle for the RA. Importantly, the experimental design consisting of the simultaneous use of 38 high-volumetric flow-rate air samplers, operated at two locations (MT and RA) and at various heights (MT, two heights; RA, five heights), allows for assessment of the dynamics of bioaerosol transport with respect to atmospheric processes. By combining the biological data with the meteorological parameters (e.g., PT, wind speed), we can show that daytime turbulence results in atmospheric mixing, which drives the dispersal of ground-based microorganisms to greater heights. Importantly, the turbulence is also responsible for the reverse transport of microorganisms from higher air layers to the ground. The downward movement of air masses amplifies the effect of wet and dry deposition on bioaerosols (23, 24).

The resulting steady state of bioaerosol distribution, caused by opposing vertical forces of convection and gravity, is reflected in our taxonomic analysis of airborne microorganisms. As an outcome, we show the height-associated differences in the composition and abundance of the top 10 most prevalent taxonomic groups (Fig. 1), with pronounced differences for bacteria, Ascomycota, and Basidiomycota (Figs. 2–4). Furthermore, our approach uses differences in airborne microbial community composition as a proxy for vertical transport, without the need for artificial tracer substances. As a result, airborne microbial taxa can be associated with either ground or height in a statistically significant manner with high taxonomic and spatial resolution. This is exemplified by the association of specific bacteria with height, which are only being transported toward the ground via atmospheric turbulence during the daytime. The lack of atmospheric turbulence at night, in combination with enhanced nighttime wet deposition, results in substantially reduced bacterial abundance in the near-surface air layers. Consequently, the air layers above MLH act as a sink for airborne biomass that replenishes the near-surface air layers after sunrise the following day. During nighttime, wet deposition is triggered by stable stratification, due to near-surface air temperatures that are lower than those at 200- to 300-m height (Fig. 2*D*) and by condensation of air masses. In addition to physical factors, microbial abundances are impacted by biological processes, such as sporulation. One example is the active release of basidiospores, which occurs only within a small range of meteorological parameters at nighttime (25, 26). Consequently, this diurnally repeating pattern of daytime atmospheric turbulence and nighttime stratification shapes the long-term amassment and enrichment of airborne biomass at the respective heights. Therefore, these diurnal patterns impact the scale and boundaries, as well as the dynamics and natural variability of airborne biomass, resulting in the biological organization of the planetary air ecosystem.

Our experimental design combines physical and biological measurements, revealing the height-specific stratification of airborne microorganisms in the vertical air column. Based on our observations, we propose a model in which the heating of the planetary surface by nonionizing radiation (infrared + ultraviolet [UV]) results in increased temperatures within ground-level air masses, thereby driving the diel cycle of ground-originated microorganisms. Conversely, the absence of day/night temperature differences results in the air microbiome being entirely devoid of diel fluctuations at higher altitudes. As air layers in the lower atmosphere are exposed to similar amounts of incoming solar radiation, air/surface temperature is indicated as the key driver governing air microbiome composition and dynamics. Our observations are also relevant with respect to global warming, as rising atmospheric temperatures will potentially cause substantial disturbances to the currently recorded airborne microbial community compositions. A warmer atmosphere will therefore result in a greater abundance of microorganisms reaching greater heights and dispersing across larger distances. This effect will predominantly impact bacteria that use daytime convection for aerial transport but will have a particularly large influence on the aerodynamically shaped ascospores. The latter include a substantial number of taxa that have been identified as plant pests and will potentially result in a wider spread of plant diseases. In contrast, increasing temperatures are not expected to impact the range of Basidiomycota dispersal. However, increased nighttime temperatures potentially will lead to altered sporulation patterns of this important class of saprophytes. Lastly, changes in atmospheric temperature are expected to disrupt currently observed circadian rhythms of microorganisms, with cyanobacteria being one such example (27). Future research will need to investigate the extend of interconnectedness between the physiological response of the microbial circadian rhythm and the diel cycle of airborne microorganisms, which is considered to be a combination of physical and biological processes.

Our model for the stratification of biomass in the vertical air column is further supported by the species-level analysis of ground- and height-based bacterial groups. We show that radio-tolerant bacteria, as well as cyanobacteria (*SI Appendix*, Fig. S79) have evolved physiological traits that provide for their resilience in one of the harshest ecological settings. Both classes of organisms either harvest light via their chlorophyll-based photosynthetic systems (28) or have adapted to survive at significantly elevated levels of ionizing radiation (22).

The discovery of radio-tolerant bacteria being associated with greater heights in the atmosphere opens the possibility that these organisms’ lifecycles partially occur in the atmosphere. The large tolerance of this bacterial group toward environmental stresses, including resistance against ionizing and nonionizing radiation, extreme desiccation, and temperature, has allowed them to gain an evolutionary advantage relative to strictly ground-based organisms. Radio-tolerant bacteria, as well as cyanobacteria, may therefore serve as examples of multiple taxonomic groups that validate the proposed model for the atmospheric stratification of airborne biomass.

Lastly, our vertical testing methods can form the experimental basis for investigating whether the evolutionary principle of radio tolerance has evolved at higher layers of the planetary atmosphere. Alternatively, the observed stratification of radio-tolerant bacteria could merely be the result of airborne biomass enrichment in a gaseous but stratified vertical ecosystem.

## Materials and Methods

### Sample Collection.

All air samples were collected using dry electret filters and SASS3100 air samplers (Research International), operated at an airflow rate of 300 L/min. For the MT experiment, samples were collected in duplicates for each sampling height and time point (total of 130 filters for the entire MT experiment). For each flight of the RA experiment, 12 air filters were collected on the aircraft at each of the four sampling altitudes for a total of 48 filters per flight. Concurrently, an additional six ground-level air samples were collected for each altitude, resulting in a total of 360 collected air samples for the RA experiment.

### Filter Processing and DNA Extraction.

While filters from the MT experiment were processed individually, multiple filters from the RA experiment were pooled to obtain sufficient DNA for library preparation and sequencing. Biomass removal from the filters and subsequent DNA extraction were performed as previously described (17, 18). In short, biomass was removed from the filters with a Triton X-100–containing phosphate-buffered saline wash buffer, followed by biomass concentration from the resulting wash solution onto an Anodisc filter (Cytiva Whatman). The Anodisc filter was then subjected to DNA extraction with the DNeasy PowerWater Kit, following the manufacturer’s recommendations with some slight modifications as previously described.

### DNA Library Preparation and Metagenomic Sequencing.

Libraries from the DNA extracts were prepared with the Accel-NGS 2S Plus DNA Library Kit (Swift Biosciences), following the instructions provided in the kit. All libraries were dual barcoded with the 2S Dual Indexing Kit (Swift Biosciences). Subsequent sequencing was performed on the Illumina HiSeq2500 platform in rapid mode and at a read length of 250 bp paired end.

### Data Processing and Metagenomic Analysis.

Resulting metagenomic datasets for the air samples were processed with Cutadapt v.1.8.1 (29) to remove adapter sequences and quality trim the sequencing reads with a Phred quality score cutoff of Q20. Potential human contamination was then identified and subsequently removed by aligning the trimmed data against the GRCh38 human genome reference, using Bowtie2 v.2.4.1 with default parameters (30). Datasets that revealed significant human contamination were also subjected to a co-occurrence analysis to identify and remove sequencing reads derived from human-associated microorganisms (Spearman *R* > 0.8) (31). Mapped and unmapped reads were separated using Samtools v.1.10 (32). The unmapped portion of the sequencing reads was then aligned against the National Center for Biotechnology Information (NCBI) nonredundant (nr) protein database using Kaiju v1.7.2 (33), and taxon identifications (IDs) for the resulting alignments were subsequently assigned with Megan v6.18 (34).

### Statistical Analysis.

For each time point, relative abundances of the technical replicates were averaged. We used the Wilcoxon test (35) to assess significance of differences in DNA yields between day and night samples as implemented in R v.4.0.2. The generalized linear regression framework (manyglm method) was used to assess association of sampling location with the corresponding multivariate species abundance, as implemented in the mvabund package (36) in R v.4.0.2. Sampling timing (day/night) was taken as the covariate in the regression model. Correlations between species response variables were accounted for, while other settings were used as default.

To visualize multivariate patterns in microbial communities, Bray–Curtis dissimilarity distances among centroids for each sample series were calculated in the vegan package (37) in R v.4.0.2. PCos (38) were used as an ordination method. A permutation test for homogeneity of multivariate dispersions (PERMDISP) (39) and analysis of variance (PERMANOVA) (40) were used to assess significance of differences between the clusters of samples as implemented in the vegan package (37) in R v.4.0.2. The alpha-diversity index Chao-1 was calculated in R v.4.0.2. Venn diagrams were created using eulerr packages (41) in R v.4.0.2.

Please refer to *SI Appendix* for detailed descriptions of the methods along with other relevant information.

## Supplementary Material

Supplementary File

## Data Availability

The raw metagenomic sequencing data reported in this paper have been deposited in the NCBI database (BioProject ID code PRJNA791118).
