# Supplementary material for: Vertical stratification of the air microbiome in the lower troposphere
Source: Proc Natl Acad Sci U S A. 2022 Feb 7;119(7):e2117293119. doi: 10.1073/pnas.2117293119 (PMC8851546; doi:10.1073/pnas.2117293119)
Supplement: Supplementary File [file pnas.2117293119.sapp.pdf]

Supplementary Information for

Vertical stratification of the air microbiome in the lower troposphere.

Daniela I. Drautz-Moses[1†], Irvan Luhung[1†], Elena S. Gusareva[1,2], Carmon Kee[1], Nicolas E. Gaultier[1], Balakrishnan N. V. Premkrishnan[1], Choou Fook Lee[1], See Ting Leong[1], Changsook Park[1], Zhei Hwee Yap[1], Cassie E. Heinle[1], Kenny J. X. Lau[1], Rikky W. Purbojati[1], Serene B. Y. Lim[1], Yee Hui Lim[1], Shruti Ketan Kutmutia[1], Ngu War Aung[1], Elaine L. Oliveira[1], Soo Guek Ng[1], Justine Dacanay[1], Poh Nee Ang[1], Samuel D. Spence[1], Wen Jia Phung[1], Anthony Wong[1], Ryan J. Kennedy[1], Namrata Kalsi[1], Santhi Puramadathil Sasi[1], Lakshmi Chandrasekaran[1], Akira Uchida[1], Ana Carolina M. Junqueira[3], Hie Lim Kim[1,2], Rudolf Hankers[4], Thomas Feuerle[4], Ulrich Corsmeier[5], and Stephan C. Schuster[1]*

[1]Singapore Centre for Environmental Life Sciences Engineering (SCELSE), Nanyang Technological University; 60 Nanyang Drive, SBS-01N-27, Singapore 637551.

[2]The Asian School of the Environment, Nanyang Technological University; Singapore 637459.

[3]Departamento de Genética, Instituto de Biologia, Universidade Federal do Rio de Janeiro; Rio de Janeiro, 21941-590 Brazil.

[4]Institute of Flight Guidance, Technische Universität; Braunschweig, Germany.

[5]Karlsruhe Institute of Technology (KIT), Institute of Meteorology and Climate Research; Karlsruhe, Germany.

* Correspondence:
Stephan C. Schuster
SCELSE (Singapore Centre for Environmental Life Sciences Engineering)
Nanyang Technological University
60 Nanyang Drive, SBS-B3N-27
Singapore 637551
Phone: +65 6592 7890
Email: scschuster@ntu.edu.sg or stephan.c.schuster@gmail.com

†These authors contributed equally to this work.

This PDF file includes:

Materials and Methods
Overview of Sampling Sites, Study Design & Outcomes
Figures S1 to S80
Tables S1 to S5
Supplementary References

Materials and Methods

1) Sample Collection & Storage

All air samples were collected with bioaerosol electret filters (6cm diameter, particle retention efficiency of 50% for 0.5µm particles; Research International), mounted on SASS3100 dry air samplers (Research International). The air samplers were operated at a high flow rate of 300L/min. Prior to installing a new filter, the air samplers were decontaminated by placing a Kimwipe wetted with lab cleaner (10% (v/v) bleach + 1% (v/v) NaOH + 0.1% (v/v) SDS + 80mM $NaHCO_3$) onto the filter installation mount for one minute, followed by an additional round of decontamination with a Kimwipe wetted with 70% EtOH for another minute.

Upon completion of each sampling cycle, the SASS filters were returned to their original pouches, packed in Ziploc bags and transported back to the laboratory at room temperature where they were stored at −20°C until further processing.

2) Filter Processing & DNA Extraction

Biomass removal from the filters and subsequent DNA extraction were performed as previously described. In short, sterile forceps were used to remove each filter membrane from its plastic ring and transferred to a sterile 5mL tube. A wash buffer consisting of PBS and Triton X-100 (PBS-T) was then added to the tube and the filter membrane moved up and down inside the tube to dislodge the biomass. The wash buffer was subsequently squeezed out of the filter and the biomass removal repeated for two additional times with fresh aliquots of the wash buffer. For air samples collected at the meteorological tower, the combined buffer volume from the 3 wash steps for each individual filter was then filtered through a 0.02-µm Anodisc (Whatman) using a vacuum manifold (DHI), followed by DNA extraction with the DNeasy PowerWater Kit (Qiagen). Obtained DNA yields were determined with the Qubit dsDNA HS Assay kit (Thermo Fisher Scientific).

For samples collected with the research aircraft during flight 1, 6 filters collected at each height were used for DNA extraction testing to determine approximate DNA yield per filter and to inform the sampling regime for subsequent flights. As the test extractions were conducted in the field with slight variations from our standard extraction procedure, these test samples were not further processed for sequencing.
For the remaining filters from flight 1 as well as all filters from flights 2-5 and the corresponding ground samples, the wash volumes from 3 replicate filters were combined and filtered through one Anodisc to ensure sufficient DNA yield for subsequent library preparation and sequencing.

3) Library Preparation & Metagenomic Sequencing

Quantification of the extracted DNA samples was repeated with Promega's QuantiFluor DNA assay on a Promega plate-reader fluorometer immediately prior to library preparation. Whenever possible, DNA input for library preparation was normalized to 5ng based on the QuantiFluor DNA concentration and the final libraries were amplified with 8 PCR cycles. For

samples that did not meet the 5ng input DNA requirement, the PCR amplification was adjusted as follows to ensure sufficient library yield for sequencing:

DNA Input Amount [ng]	PCR Cycles
4.1-5.0	8
3.6-4.0	9
3.1-3.5	10
2.6-3.0	11
2.1-2.5	12
1.6-2.0	13
1.1-1.5	14
<1	15
all blanks	8

Libraries were constructed with the Accel-NGS 2S Plus DNA Kit (Swift Biosciences) as previously described. All libraries were dual-barcoded with 2S dual indices (Swift Biosciences). Average library size was determined on an Agilent Tapestation 4200 and library quantitation was performed using Promega's QuantiFluor DNA assay on a Promega plate-reader fluorometer. Libraries were then diluted to 4nM and the concentrations were validated by qPCR on a QuantStudio-3 real-time thermocycler (Applied Biosystems) using the Library Quantification Kit for Illumina Platforms (Roche). Sequencing was subsequently performed on the Illumina HiSeq2500 platform in rapid mode at a read length of 251-bp paired end (Illumina HiSeq 2500 V2 rapid sequencing chemistry).

Filter and reagent blanks were processed with the same protocols as the air samples. All blank libraries were amplified with 8 PCR cycles.

4) Raw-Read Processing & Human Genome Reads Removal Analysis Pipeline

Adapter sequences and bases with Phred quality score less than 20 (Q < 20) were removed from the metagenomic reads during the quality trimming process using Cutadapt. The trimmed reads were then used for downstream analysis. In order to screen the obtained metagenomic datasets for potential human contamination, the following pipeline was implemented: Quality-trimmed sequencing data were mapped against the human genome reference (version GRCh38) using the ultrafast and memory efficient aligner Bowtie2 (version 2.4.1) with default parameters. The reference human genome was downloaded from NCBI on 9 September 2020. The Sequence Alignment Map (SAM)-formatted file generated by Bowtie2 was then converted to Binary Alignment Map (BAM) format before further analysis with Samtools, version 1.10. Mapped (human genome-associated) and unmapped reads were separated from the BAM file and the unmapped reads were further analyzed for the presence of correctly paired sequencing reads (both, paired forward and reverse reads are present) using Samtools. Singletons (only one of the paired reads is present, i.e., either forward or reverse) were discarded. Overall statistics showing the total read count for each sample and the number of unmapped reads is given below in Table S2. Unmapped, paired reads (after removal of human and human-associated microbial contamination) were aligned against the NCBI non-redundant (nr) protein database downloaded on 22 November 2019 using the

taxonomic classification tool Kaiju, version 1.7.2. An overview of the entire analysis pipeline is shown below in Figure S1.

Resulting alignments were imported into MEGAN v6.18, which assigns taxon IDs based on NCBI taxonomy. To achieve the desired taxonomic specificity, we used the following filtering parameters: min score 100 (bit score), max expected 0.01 (e value), top percent 10 (top 10% of the highest bit score), min support 25 (minimum number of reads required for taxonomic assignment), lowest common ancestry (LCA) percent 100 (naive), and min complexity 0.33 (sequence complexity). LCA for each read on the NCBI taxonomy is assigned using MEGAN's LCA algorithm. In instances where all of the filtering criteria were fulfilled, reads were assigned to various levels of taxonomic classification ranging from domain to species. In our study, species-level classification was only reached if at least 25 reads uniquely aligned to a single species in the database with a bit score equivalent to a 100% match on the protein level over at least 50% of the 250-bp read. Due to limits of existing public sequence databases, some sequencing reads did not result in meaningful alignments and were classified in the no-hits category. Unassigned reads were categorized in instances when alignments were made but did not fulfill one of the filtering parameters, namely the bit score.

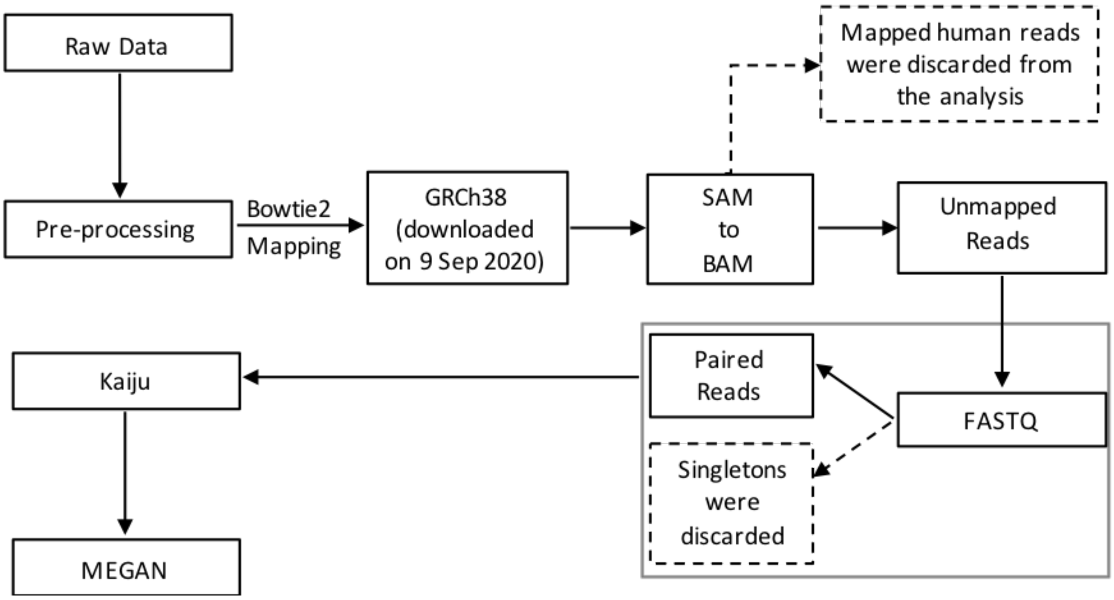

Figure S1. Human reads removal and overall analysis pipeline**.**

5) <u>Human Microbiome Co-Occurrence Analysis</u>

In addition to human genome reads removal, datasets that revealed significant human contamination were subjected to additional filtering prior to metagenome analysis (see Table S2). For these datasets, a co-occurrence analysis was conducted to identify microbial species that could be strongly associated with *Homo sapiens* (Spearman R>0.8). A list of all human-associated species identified by the co-occurrence analysis is provided in Table S1.

Table S1. List of microbial species that were identified as "human-associated" by co-occurrence analysis and removed from the RA dataset prior to metagenome analysis.

Plasmodium ovale	*Corynebacterium tuberculostearicum*	*Bacillus cereus*
Enterococcus faecalis	*Salmonella enterica*	*Malassezia restricta*
Bacillus thuringiensis	*Vibrio vulnificus*	*Filimonas lacunae*
Mycobacterium tuberculosis	*Cutibacterium acnes*	*Kocuria rhizophila*
Escherichia coli	*Mycobacteroides abscessus*	*Clostridioides difficile*
Streptococcus pneumoniae	*Staphylococcus hominis*	*Finegoldia magna*
Acinetobacter baumannii	*Corynebacterium pseudogenitalium*	*Lawsonella clevelandensis*
Lactobacillus crispatus	*Cronobacter sakazakii*	*Klebsiella pneumoniae*
Chlamydia trachomatis	*Corynebacterium aurimucosum*	*Malassezia globosa*
Burkholderia multivorans		

Reference genomes for the human-associated microbial species were downloaded individually from NCBI. The affected datasets were then mapped to these reference genomes using Bowtie2. Mapped and unmapped reads were separated using Samtools and only paired, unmapped reads (after removal of human and human-associated microbiome reads) were used for alignment to the NCBI non-redundant (nr) protein database and downstream analyses.

Table S2. Overall statistics of total raw sequencing reads and remaining unmapped paired reads for Kaiju analysis after removal of human and human-associated microbial contamination for all samples from the MT and RA experiments.

Sample ID	Raw Reads (R1+R2)	Trimmed Reads (R1+R2)	GRCh38 mapping %	Unmapped Singletons	Unmapped Paired Reads	Human Microbiome Mapping %	Unmapped Singletons	Unmapped Paired Reads	Reads Used for Kaiju
IBUF361	37,151,624	37,099,108	7.24	136,544	34,378,884	1.06%	22,994	33,990,336	5,000,000
IBUF362	37,781,046	37,682,274	14.87	235,656	32,021,574	1.54%	26,460	31,501,840	5,000,000
IBUF363	36,241,292	35,924,538	11.58	212,836	31,712,930	1.52%	32,493	31,197,700	5,000,000
IBUF364	35,332,532	35,280,664	8.41	174,728	32,268,778	1.49%	51,580	31,736,070	5,000,000
IBUF365	34,485,764	34,131,000	0.18	31,008	34,062,772	0.54%	36,539	33,843,828	5,000,000
IBUF366	32,918,850	32,872,984	0.28	31,960	32,772,840	0.16%	14,757	32,705,674	5,000,000
IBUF367	31,684,086	31,627,844	0.10	20,184	31,589,678	0.27%	17,784	31,487,374	5,000,000
IBUF368	31,000,152	30,956,362	0.13	19,548	30,911,818	0.67%	40,063	30,663,246	5,000,000
IBUF369	5,445,108	5,435,830	7.56	16,176	5,021,014	1.10%	4,263	4,961,652	4,961,652
IBUF370	5,344,250	5,336,900	9.26	22,852	4,836,854	1.50%	6,699	4,757,584	4,757,584
IBUF371	5,301,660	5,293,076	7.74	20,140	4,878,248	1.33%	4,758	4,808,418	4,808,418
IBUF372	4,856,270	4,848,248	8.78	17,672	4,418,284	1.16%	2,523	4,364,526	4,364,526
IBUF373	5,024,030	5,017,022	8.36	15,984	4,593,738	1.53%	2,980	4,520,376	4,520,376
IBUF374	4,949,586	4,943,084	12.25	21,376	4,332,008	1.67%	3,193	4,256,308	4,256,308
IBUF375	5,050,158	5,043,348	6.74	16,384	4,699,482	1.17%	2,642	4,641,690	4,641,690
IBUF376	5,462,990	5,453,640	7.87	19,076	5,019,824	1.15%	2,875	4,959,064	4,959,064
IBUF377	6,377,274	6,366,554	0.15	4,700	6,355,786	0.16%	2,005	6,343,474	6,343,474
IBUF378	5,502,002	5,494,262	0.10	3,780	5,487,896	0.19%	1,999	5,475,440	5,475,440
IBUF379	4,692,950	4,686,846	0.25	2,940	4,674,252	0.16%	1,437	4,665,458	4,665,458
IBUF380	5,069,568	5,063,088	0.08	3,020	5,058,070	0.17%	1,672	5,047,674	5,047,674
IBUF381	5,492,724	5,470,868	13.12	25,148	4,746,558	1.41%	2,503	4,677,364	4,677,364
IBUF382	5,033,800	4,998,802	11.98	21,716	4,394,568	1.37%	2,336	4,332,088	4,332,088
IBUF383	5,325,516	5,318,696	9.25	20,968	4,821,490	1.59%	3,027	4,741,728	4,741,728
IBUF384	4,503,528	4,496,802	10.13	21,500	4,035,900	1.61%	2,486	3,968,636	3,968,636
IBUF385	5,776,900	5,737,750	9.74	20,356	5,173,736	1.53%	3,027	5,091,388	5,091,388
IBUF386	6,111,676	6,093,910	12.21	25,348	5,343,386	1.57%	3,086	5,256,390	5,256,390
IBUF387	5,553,302	5,545,402	11.80	28,876	4,883,668	1.64%	3,182	4,800,406	4,800,406
IBUF388	6,502,648	6,463,464	14.20	45,304	5,534,556	1.59%	3,511	5,442,884	5,442,884
IBUF389	4,833,004	4,826,034	12.11	21,484	4,236,238	1.50%	2,466	4,170,246	4,170,246
IBUF390	5,656,406	5,648,864	9.85	24,028	5,086,236	1.50%	3,281	5,006,840	5,006,840
IBUF391	5,462,884	5,455,928	11.22	26,928	4,837,002	1.48%	2,885	4,762,554	4,762,554

Sample ID	Raw Reads (R1+R2)	Trimmed Reads (R1+R2)	GRCh38 mapping %	Unmapped Singletons	Unmapped Paired Reads	Human Microbiome Mapping %	Unmapped Singletons	Unmapped Paired Reads	Reads Used for Kaiju
IBUF392	5,334,816	5,323,958	12.66	23,684	4,643,894	1.43%	2,645	4,574,840	4,574,840
IBUF393	6,832,584	6,824,330	6.87	26,964	6,348,654	1.03%	3,790	6,279,754	6,279,754
IBUF394	5,704,846	5,697,928	6.21	17,284	5,339,982	1.02%	2,907	5,282,656	5,282,656
IBUF395	6,395,454	6,388,134	7.70	21,456	5,890,864	1.01%	3,398	5,827,790	5,827,790
IBUF396	5,642,618	5,636,406	7.50	18,596	5,209,286	0.92%	2,845	5,158,716	5,158,716
IBUF397	5,612,496	5,605,766	0.09	7,688	5,598,550	0.39%	4,192	5,572,742	5,572,742
IBUF398	5,649,990	5,642,852	0.20	7,992	5,629,610	0.37%	3,899	5,604,916	5,604,916
IBUF399	5,058,674	5,052,080	0.70	7,144	5,015,092	0.35%	3,660	4,993,964	4,993,964
IBUF400	5,050,390	5,044,098	0.13	6,724	5,035,892	0.36%	3,604	5,014,388	5,014,388
IBUF401	5,607,762	5,599,656	0.10	5,788	5,592,462	0.31%	3,377	5,571,550	5,571,550
IBUF402	6,534,944	6,524,448	3.21	17,680	6,310,378	0.31%	4,010	6,287,024	6,287,024
IBUF403	5,543,350	5,535,244	0.16	5,532	5,524,976	0.29%	3,042	5,505,828	5,505,828
IBUF404	4,919,974	4,911,834	0.10	6,252	4,905,210	0.33%	3,442	4,885,746	4,885,746
IBUF405	5,134,572	5,125,184	8.65	15,748	4,677,764	1.34%	2,404	4,612,626	4,612,626
IBUF406	4,981,600	4,973,930	10.14	18,376	4,465,164	1.62%	2,718	4,389,992	4,389,992
IBUF407	5,144,480	5,137,384	11.77	23,472	4,526,910	1.53%	2,978	4,454,838	4,454,838
IBUF408	4,357,942	4,350,276	7.02	16,880	4,040,700	1.51%	2,599	3,976,966	3,976,966
IBUF409	5,183,492	5,174,624	10.62	19,332	4,620,424	1.76%	3,057	4,535,942	4,535,942
IBUF410	6,190,470	6,170,938	9.59	30,144	5,571,312	1.69%	3,825	5,473,122	5,473,122
IBUF411	6,271,900	6,232,744	11.07	24,712	5,536,388	3.47%	17,850	5,326,294	5,326,294
IBUF412	5,304,368	5,289,384	10.87	22,116	4,709,118	2.61%	10,460	4,575,684	4,575,684
IBUF413	5,509,286	5,493,430	12.09	24,768	4,822,970	1.52%	2,938	4,746,802	4,746,802
IBUF414	5,928,642	5,912,710	10.02	22,720	5,314,332	1.60%	2,971	5,226,192	5,226,192
IBUF415	5,888,976	5,879,644	8.50	19,144	5,375,142	1.65%	3,448	5,283,184	5,283,184
IBUF416	5,206,232	5,198,642	13.49	27,904	4,490,454	1.48%	2,906	4,421,114	4,421,114
IBUF417	5,653,478	5,639,076	7.70	17,264	5,200,620	1.08%	2,695	5,142,004	5,142,004
IBUF418	5,833,372	5,816,982	7.73	18,472	5,362,734	0.87%	2,739	5,313,136	5,313,136
IBUF419	5,770,632	5,759,134	13.29	33,292	4,985,240	0.98%	2,892	4,933,394	4,933,394
IBUF420	5,458,910	5,450,596	9.07	20,828	4,951,114	1.03%	2,820	4,897,382	4,897,382
IBUF421	5,787,950	5,781,072	0.06	7,240	5,775,516	0.30%	4,629	5,753,348	5,753,348
IBUF422	4,973,074	4,964,158	0.07	4,624	4,959,588	0.29%	2,681	4,942,522	4,942,522
IBUF423	4,389,966	4,374,162	0.10	3,980	4,368,592	0.29%	2,235	4,353,762	4,353,762
IBUF424	5,630,918	5,621,708	0.09	4,888	5,615,154	0.29%	2,898	5,596,098	5,596,098
IBUF425	5,559,202	5,549,604	0.06	4,584	5,545,010	0.28%	2,866	5,526,486	5,526,486
IBUF426	4,906,806	4,900,076	0.20	5,916	4,889,002	0.30%	3,897	4,870,256	4,870,256
IBUF427	5,907,870	5,897,188	0.08	6,116	5,890,984	0.35%	3,549	5,867,050	5,867,050
IBUF428	5,027,516	5,019,190	0.50	5,464	4,992,768	0.31%	2,804	4,974,638	4,974,638
IBUF429	4,701,108	4,694,248	19.77	34,652	3,757,494	2.44%	3,060	3,662,626	3,662,626
IBUF430	4,773,580	4,766,422	21.24	37,976	3,744,540	2.56%	3,732	3,644,916	3,644,916
IBUF431	5,055,672	5,048,378	22.57	46,528	3,897,104	2.45%	3,678	3,798,024	3,798,024
IBUF432	4,081,684	4,064,306	20.31	29,340	3,231,622	2.46%	2,852	3,149,344	3,149,344
IBUF433	4,984,660	4,939,474	16.06	34,120	4,137,462	2.33%	3,341	4,037,916	4,037,916
IBUF436	5,297,274	5,261,816	16.29	29,724	4,397,354	2.35%	3,143	4,290,806	4,290,806
IBUF437	5,508,074	5,499,926	12.94	30,168	4,780,434	2.14%	4,378	4,673,988	4,673,988
IBUF438	8,970,484	8,950,054	13.82	62,640	7,697,886	2.31%	8,043	7,512,374	7,512,374
IBUF439	6,075,630	6,064,406	13.81	32,060	5,218,938	2.04%	4,058	5,108,376	5,108,376
IBUF440	5,485,296	5,476,536	13.32	27,856	4,740,106	2.25%	3,670	4,629,828	4,629,828
IBUF441	4,849,762	4,842,476	13.85	32,240	4,163,934	1.56%	2,963	4,095,822	4,095,822
IBUF442	6,558,236	6,548,148	10.64	41,216	5,841,188	2.39%	12,516	5,689,064	5,689,064
IBUF443	4,934,756	4,926,892	12.04	24,264	4,327,720	1.82%	3,272	4,245,700	4,245,700
IBUF444	6,125,588	6,116,604	12.19	28,584	5,363,948	1.55%	3,615	5,277,032	5,277,032
IBUF445	4,580,940	4,574,064	0.16	4,108	4,565,850	0.29%	2,369	4,550,222	4,550,222
IBUF446	4,782,566	4,772,444	0.20	4,184	4,761,736	0.27%	2,245	4,746,802	4,746,802
IBUF447	5,971,806	5,961,966	1.50	8,504	5,870,480	0.32%	3,815	5,847,850	5,847,850
IBUF448	6,572,600	6,562,330	0.31	6,428	6,540,088	0.53%	5,935	6,499,210	6,499,210
IBUF449	5,605,634	5,597,044	0.18	4,144	5,586,066	0.30%	3,006	5,566,188	5,566,188
IBUF450	4,484,386	4,477,710	0.24	3,368	4,465,920	0.31%	2,523	4,449,504	4,449,504

Sample ID	Raw Reads (R1+R2)	Trimmed Reads (R1+R2)	GRCh38 mapping %	Unmapped Singletons	Unmapped Paired Reads	Human Microbiome Mapping %	Unmapped Singletons	Unmapped Paired Reads	Reads Used for Kaiju
IBUF451	5,141,562	5,133,496	0.19	3,256	5,122,904	0.29%	2,815	5,104,990	5,104,990
IBUF452	4,895,976	4,887,734	0.14	3,228	4,880,236	0.30%	2,902	4,862,856	4,862,856
IBUF453	5,603,876	5,578,876	9.10	17,680	5,066,710	2.18%	6,049	4,950,322	4,950,322
IBUF454	6,116,460	6,046,384	11.62	21,760	5,338,278	2.28%	7,154	5,209,562	5,209,562
IBUF455	5,972,180	5,956,426	13.09	26,564	5,170,224	2.03%	4,320	5,060,856	5,060,856
IBUF456	4,877,102	4,838,986	10.42	18,436	4,329,928	2.47%	7,893	4,214,954	4,214,954
IBUF457	4,912,764	4,904,262	8.44	15,508	4,486,618	1.44%	3,160	4,418,764	4,418,764
IBUF458	5,065,534	5,049,504	9.62	20,016	4,558,680	1.54%	3,067	4,485,354	4,485,354
IBUF459	5,409,254	5,389,412	11.75	23,948	4,750,212	2.53%	5,395	4,624,546	4,624,546
IBUF460	5,691,016	5,673,058	10.80	22,044	5,055,082	1.56%	3,448	4,972,588	4,972,588
IBUF461	5,594,564	5,583,300	8.12	19,084	5,125,292	1.92%	8,846	5,018,034	5,018,034
IBUF462	7,564,244	7,552,754	7.93	26,500	6,947,094	1.48%	6,723	6,837,760	6,837,760
IBUF463	6,128,388	6,118,526	9.02	20,828	5,561,370	1.39%	3,513	5,480,604	5,480,604
IBUF464	6,616,504	6,606,356	9.68	29,464	5,959,400	1.29%	4,057	5,878,248	5,878,248
IBUF465	6,464,086	6,454,208	10.75	23,380	5,754,546	1.24%	4,134	5,679,092	5,679,092
IBUF466	6,657,046	6,646,658	7.11	18,976	6,169,116	1.27%	3,785	6,086,724	6,086,724
IBUF467	5,951,994	5,942,058	7.21	18,996	5,508,850	1.28%	4,629	5,433,658	5,433,658
IBUF468	7,818,196	7,805,292	8.42	29,116	7,141,194	1.47%	6,148	7,030,238	7,030,238
IBUF469	7,904,778	7,889,942	0.15	5,988	7,876,824	0.64%	8,459	7,817,798	7,817,798
IBUF470	6,208,912	6,197,476	0.14	5,572	6,187,532	0.48%	4,865	6,152,968	6,152,968
IBUF471	5,504,294	5,495,390	0.16	4,932	5,485,188	0.36%	3,183	5,462,114	5,462,114
IBUF472	5,724,040	5,714,796	0.17	5,288	5,703,912	0.36%	3,499	5,679,602	5,679,602
IBUF473	4,993,736	4,985,406	0.14	5,588	4,977,152	0.43%	3,566	4,952,118	4,952,118
IBUF474	7,663,826	7,652,058	0.22	7,920	7,633,202	0.41%	4,780	7,597,454	7,597,454
IBUF475	8,423,788	8,409,270	0.18	8,368	8,391,798	0.35%	5,023	8,357,388	8,357,388
IBUF476	4,977,934	4,969,798	0.15	5,452	4,961,110	0.53%	4,717	4,930,282	4,930,282
APG081	5,444,248	5,420,262	0.27%	5,568	5,404,034	-	-	-	5,404,034
APG082	4,401,416	4,360,932	0.25%	4196	4,349,068	-	-	-	4,349,068
APG083	6,236,766	6,227,786	0.08%	5,444	6,221,148	-	-	-	6,221,148
APG084	5,329,934	5,322,134	0.06%	4,564	5,317,768	-	-	-	5,317,768
APG085	5,366,658	5,359,332	0.08%	3,940	5,353,882	-	-	-	5,353,882
APG086	5,040,666	5,032,736	1.19%	5,580	4,971,694	-	-	-	4,971,694
APG087	5,200,810	5,193,068	0.06%	3,640	5,189,074	-	-	-	5,189,074
APG088	5,266,628	5,259,112	0.22%	3,876	5,246,610	-	-	-	5,246,610
APG089	7,772,840	7,685,910	0.92%	9,744	7,612,508	-	-	-	7,612,508
APG090	6,116,854	6,088,276	0.12%	5,124	6,079,418	-	-	-	6,079,418
APG091	4,767,732	4,746,158	0.28%	5,316	4,731,586	-	-	-	4,731,586
APG092	5,267,414	5,249,104	1.02%	6,992	5,193,780	-	-	-	5,193,780
APG093	4,977,856	4,962,270	0.30%	5,268	4,946,000	-	-	-	4,946,000
APG094	4,892,812	4,882,212	0.09%	3,168	4,876,846	-	-	-	4,876,846
APG095	5,401,758	5,390,402	0.17%	5,388	5,379,688	-	-	-	5,379,688
APG096	5,485,242	5,474,236	0.15%	5,688	5,464,662	-	-	-	5,464,662
APG097	6,373,202	6,364,768	0.11%	6,916	6,355,786	-	-	-	6,355,786
APG098	5,590,238	5,583,018	0.10%	5,648	5,575,826	-	-	-	5,575,826
APG099	5,421,028	5,412,294	0.07%	7,124	5,406,550	-	-	-	5,406,550
APG100	4,935,934	4,928,538	0.07%	6,256	4,923,482	-	-	-	4,923,482
APG101	5,066,346	5,058,714	0.09%	6,316	5,052,402	-	-	-	5,052,402
APG102	5,118,034	5,110,084	0.20%	6,288	5,098,282	-	-	-	5,098,282
APG103	5,498,180	5,488,916	0.09%	6,464	5,482,176	-	-	-	5,482,176
APG104	5,834,176	5,824,646	0.10%	6,212	5,817,042	-	-	-	5,817,042
APG107	7,880,200	7,841,410	0.96%	10,560	7,763,194	-	-	-	7,763,194
APG108	6,635,120	6,601,050	1.56%	10,516	6,495,640	-	-	-	6,495,640
APG109	6,635,470	6,626,280	0.10%	5,168	6,618,426	-	-	-	6,618,426
APG110	6,584,420	6,575,324	0.11%	5,948	6,566,694	-	-	-	6,566,694
APG111	5,599,604	5,592,948	0.19%	3,684	5,581,296	-	-	-	5,581,296
APG112	6,485,042	6,475,070	0.09%	4,236	6,468,072	-	-	-	6,468,072
APG113	5,444,346	5,396,276	0.17%	4,616	5,385,810	-	-	-	5,385,810

Sample ID	Raw Reads (R1+R2)	Trimmed Reads (R1+R2)	GRCh38 mapping %	Unmapped Singletons	Unmapped Paired Reads	Human Microbiome Mapping %	Unmapped Singletons	Unmapped Paired Reads	Reads Used for Kaiju
APG114	5,741,778	5,709,462	0.19%	5,364	5,697,510	-	-	-	5,697,510
APG115	5,847,566	5,827,592	0.32%	6,424	5,807,416	-	-	-	5,807,416
APG116	5,421,512	5,393,578	1.96%	10,192	5,285,340	-	-	-	5,285,340
APG117	5,703,432	5,668,172	0.83%	8,352	5,619,172	-	-	-	5,619,172
APG118	5,690,706	5,670,818	0.52%	6,620	5,639,588	-	-	-	5,639,588
APG119	5,383,158	5,349,680	0.60%	5,416	5,316,114	-	-	-	5,316,114
APG120	5,132,462	5,087,414	0.80%	4,324	5,045,806	-	-	-	5,045,806
APG121	4,761,260	4,750,604	0.06%	3,272	4,746,880	-	-	-	4,746,880
APG122	5,370,972	5,360,102	0.07%	4,492	5,355,476	-	-	-	5,355,476
APG123	6,383,082	6,373,230	0.09%	6,664	6,365,720	-	-	-	6,365,720
APG124	5,938,096	5,930,362	0.59%	5,936	5,893,942	-	-	-	5,893,942
APG125	4,986,048	4,978,400	0.09%	5,936	4,972,296	-	-	-	4,972,296
APG126	5,391,862	5,383,464	0.16%	6,912	5,373,206	-	-	-	5,373,206
APG127	6,380,200	6,345,536	0.20%	5,408	6,331,230	-	-	-	6,331,230
APG128	5,311,962	5,287,680	0.39%	4,992	5,265,892	-	-	-	5,265,892
APG129	6,124,290	6,073,234	0.87%	4,292	6,019,168	-	-	-	6,019,168
APG130	4,401,070	4,376,472	0.23%	2,816	4,365,582	-	-	-	4,365,582
APG133	5,778,222	5,762,424	0.27%	6,000	5,745,380	-	-	-	5,745,380
APG134	6,716,078	6,644,932	0.17%	7,424	6,631,708	-	-	-	6,631,708
APG135	7,368,504	7,355,116	0.09%	8,524	7,346,088	-	-	-	7,346,088
APG136	5,721,116	5,713,480	0.06%	6,476	5,708,248	-	-	-	5,708,248
APG137	5,774,556	5,766,704	0.06%	4,912	5,762,142	-	-	-	5,762,142
APG138	6,717,138	6,706,862	0.09%	7,932	6,698,664	-	-	-	6,698,664
APG139	6,686,664	6,677,892	0.09%	9,112	6,669,274	-	-	-	6,669,274
APG140	5,892,604	5,885,374	0.09%	6,904	5,878,228	-	-	-	5,878,228
APG141	6,138,424	6,129,362	0.09%	7,040	6,121,810	-	-	-	6,121,810
APG142	6,662,626	6,653,440	0.10%	9,280	6,644,578	-	-	-	6,644,578
APG143	6,359,796	6,349,178	0.20%	7,092	6,334,796	-	-	-	6,334,796
APG144	7,088,516	7,077,226	0.24%	7,668	7,058,062	-	-	-	7,058,062
APG145	5,186,872	5,178,836	0.12%	5,264	5,171,348	-	-	-	5,171,348
APG146	4,492,638	4,486,586	0.20%	5,980	4,476,110	-	-	-	4,476,110
APG147	4,631,268	4,625,254	0.11%	4,732	4,619,148	-	-	-	4,619,148
APG148	5,424,232	5,417,292	0.06%	5,412	5,412,788	-	-	-	5,412,788
APG149	5,877,550	5,863,022	0.06%	5,200	5,858,180	-	-	-	5,858,180
APG150	5,582,624	5,568,948	0.10%	6,732	5,561,432	-	-	-	5,561,432
APG151	5,610,360	5,597,476	0.09%	8,580	5,590,446	-	-	-	5,590,446
APG152	4,813,566	4,802,880	0.08%	7,052	4,797,104	-	-	-	4,797,104
APG153	4,959,426	4,948,976	0.08%	7,444	4,943,226	-	-	-	4,943,226
APG154	5,127,604	5,116,644	0.10%	7,264	5,109,726	-	-	-	5,109,726
APG155	5,002,424	4,992,008	0.08%	6,908	4,986,148	-	-	-	4,986,148
APG156	4,230,508	4,221,736	0.07%	5,200	4,217,276	-	-	-	4,217,276
APG159	4,299,204	4,273,208	0.24%	3,304	4,262,310	-	-	-	4,262,310
APG160	5,971,676	5,942,092	1.74%	6,328	5,837,178	-	-	-	5,837,178
APG161	5,577,242	5,566,564	0.74%	6,628	5,523,760	-	-	-	5,523,760
APG162	5,937,914	5,930,652	0.07%	6,056	5,925,006	-	-	-	5,925,006
APG163	5,162,006	5,155,730	0.08%	3,456	5,150,578	-	-	-	5,150,578
APG164	5,523,280	5,514,808	0.15%	4,296	5,505,718	-	-	-	5,505,718
APG165	5,344,588	5,337,846	0.12%	5,512	5,330,258	-	-	-	5,330,258
APG166	4,757,306	4,751,002	0.55%	6,456	4,723,362	-	-	-	4,723,362
APG167	5,399,044	5,391,566	0.68%	7,340	5,352,980	-	-	-	5,352,980
APG168	5,286,526	5,276,756	0.20%	6,640	5,264,512	-	-	-	5,264,512
APG169	5,601,768	5,591,582	0.27%	7,840	5,574,408	-	-	-	5,574,408
APG170	5,582,836	5,571,670	0.16%	7,308	5,560,744	-	-	-	5,560,744
APG171	5,326,284	5,318,514	0.30%	3,396	5,301,550	-	-	-	5,301,550
APG172	5,103,656	5,097,530	0.33%	3,524	5,079,908	-	-	-	5,079,908
APG173	5,096,268	5,090,102	0.08%	5,908	5,084,464	-	-	-	5,084,464
APG174	5,260,944	5,253,966	0.10%	5,968	5,247,158	-	-	-	5,247,158

Sample ID	Raw Reads (R1+R2)	Trimmed Reads (R1+R2)	GRCh38 mapping %	Unmapped Singletons	Unmapped Paired Reads	Human Microbiome Mapping %	Unmapped Singletons	Unmapped Paired Reads	Reads Used for Kaiju
APG175	5,851,694	5,844,582	0.08%	8,136	5,837,758	-	-	-	5,837,758
APG176	5,373,526	5,365,970	0.08%	7,600	5,359,664	-	-	-	5,359,664
APG177	5,674,358	5,666,504	0.12%	9,048	5,657,428	-	-	-	5,657,428
APG178	5,581,014	5,573,414	0.09%	8,984	5,566,090	-	-	-	5,566,090
APG179	4,997,140	4,990,732	0.10%	7,380	4,984,086	-	-	-	4,984,086
APG180	4,441,618	4,435,070	0.07%	6,552	4,430,216	-	-	-	4,430,216
APG181	4,899,590	4,892,406	0.08%	6,876	4,887,016	-	-	-	4,887,016
APG182	5,218,462	5,209,096	0.14%	7,244	5,199,994	-	-	-	5,199,994
APG185	5,292,430	5,283,246	0.11%	8,304	5,275,356	-	-	-	5,275,356
APG186	6,114,052	6,101,214	0.14%	9,912	6,090,114	-	-	-	6,090,114
APG187	4,820,688	4,811,984	0.08%	4,816	4,807,056	-	-	-	4,807,056
APG188	5,305,680	5,297,492	0.11%	5,904	5,290,398	-	-	-	5,290,398
APG189	5,311,968	5,302,544	0.07%	5,848	5,297,566	-	-	-	5,297,566
APG190	5,824,014	5,814,100	0.06%	5,736	5,809,188	-	-	-	5,809,188
APG191	5,323,720	5,313,698	0.05%	4,380	5,309,786	-	-	-	5,309,786
APG192	5,832,984	5,824,134	0.05%	4,208	5,820,070	-	-	-	5,820,070
APG193	7,568,054	7,556,422	0.08%	10,024	7,547,978	-	-	-	7,547,978
APG194	5,924,508	5,917,642	0.08%	7,900	5,910,878	-	-	-	5,910,878
APG195	5,085,268	5,079,040	0.08%	6,576	5,073,308	-	-	-	5,073,308
APG196	5,048,032	5,041,968	0.09%	6,948	5,035,650	-	-	-	5,035,650
APG197	5,421,588	5,415,514	0.13%	7,556	5,406,488	-	-	-	5,406,488
APG198	4,906,036	4,899,168	0.09%	6,152	4,893,002	-	-	-	4,893,002
APG199	5,253,268	5,247,116	0.08%	6,228	5,241,552	-	-	-	5,241,552
APG200	6,846,750	6,839,012	0.11%	8,352	6,829,352	-	-	-	6,829,352
APG201	6,673,474	6,667,258	0.08%	8,608	6,659,996	-	-	-	6,659,996
APG202	6,252,870	6,245,966	0.09%	8,476	6,238,526	-	-	-	6,238,526
APG203	5,188,694	5,183,160	0.19%	7,896	5,171,324	-	-	-	5,171,324
APG204	5,082,878	5,077,434	0.07%	7,316	5,071,892	-	-	-	5,071,892
APG205	6,440,560	6,432,502	0.08%	9,216	6,425,080	-	-	-	6,425,080
APG206	6,445,350	6,439,324	0.08%	8,936	6,431,864	-	-	-	6,431,864
APG207	5,323,994	5,317,728	0.07%	7,168	5,311,998	-	-	-	5,311,998
APG208	6,528,166	6,516,918	0.08%	9,084	6,509,236	-	-	-	6,509,236

6) Collection and Processing of Filter Blanks

Filter blanks were collected daily immediately prior to sampling. The air samplers were first decontaminated as described above. After decontamination, a brand-new, clean filter was removed from its pouch and installed on the metal grid of a randomly selected air sampler. Without running the air sampler, the filter was then collected back and placed into its original pouch for transportation to the laboratory. Two filter blanks were collected per sampling dat. All filter blanks were processed alongside the air samples with the same protocols as described above.

The analyses of the filter blanks did not reveal any significant contamination introduced by the air sampling equipment and downstream laboratory processing. Differences in DNA yields as well as taxonomic composition between the blanks and the MT and RA air samples are shown in Figure S2. After filter processing, the DNA concentrations of 6 of the 10 filter blank samples were below the detection limit of the Qubit 2.0 fluorometer. The DNA yields of the remaining four filter blanks were on average 29- and 20-fold lower than those of the MT and RA air samples, respectively (Fig. S2).

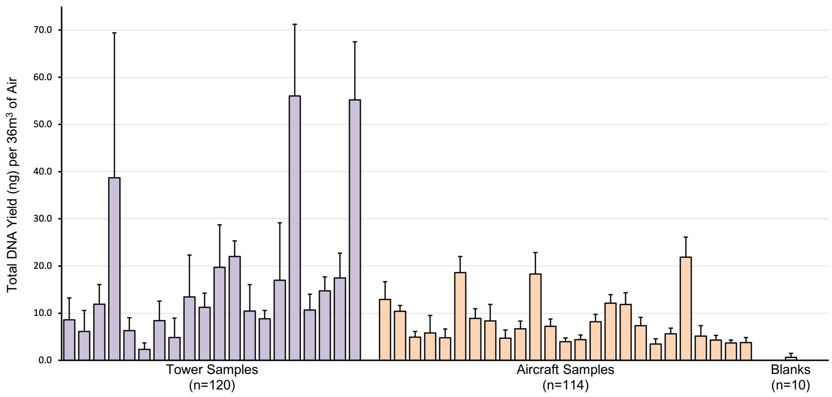

Figure S2. Comparison of total DNA yield (ng) obtained for air samples from the MT and RA experiments and filter blanks. Columns represent the mean values of each dataset Average DNA yields were calculated for day (10am – 8pm) and night (10pm – 8am) samples, both at the tower top and bottom for each sampling day (MT experiment). For the RA experiment, for each flight, averages were calculated for the samples of each sampling height.

Extracted DNA from the filter blanks was subsequently processed with the default library enrichment conditions as for the air samples (8 PCR cycles). The number of sequencing reads generated for the filter blanks was very low (average of 7,524 paired reads per filter blank), compared to the MT (average of 2,804,028 paired reads per sample) and RA (average of 3,810,256 paired reads per sample) air samples. Further, only a small number of reads from the filter blank samples resulted in meaningful assignments at phylum level (332 – 1,474 assigned reads) with the majority of them belonging to human-associated Proteobacteria and Actinobacteria taxa (Fig. S3). As described in detail in the method section, reads from our air samples that belong to these human associated taxa were removed prior to taxonomic assignment and subsequent downstream analyses.

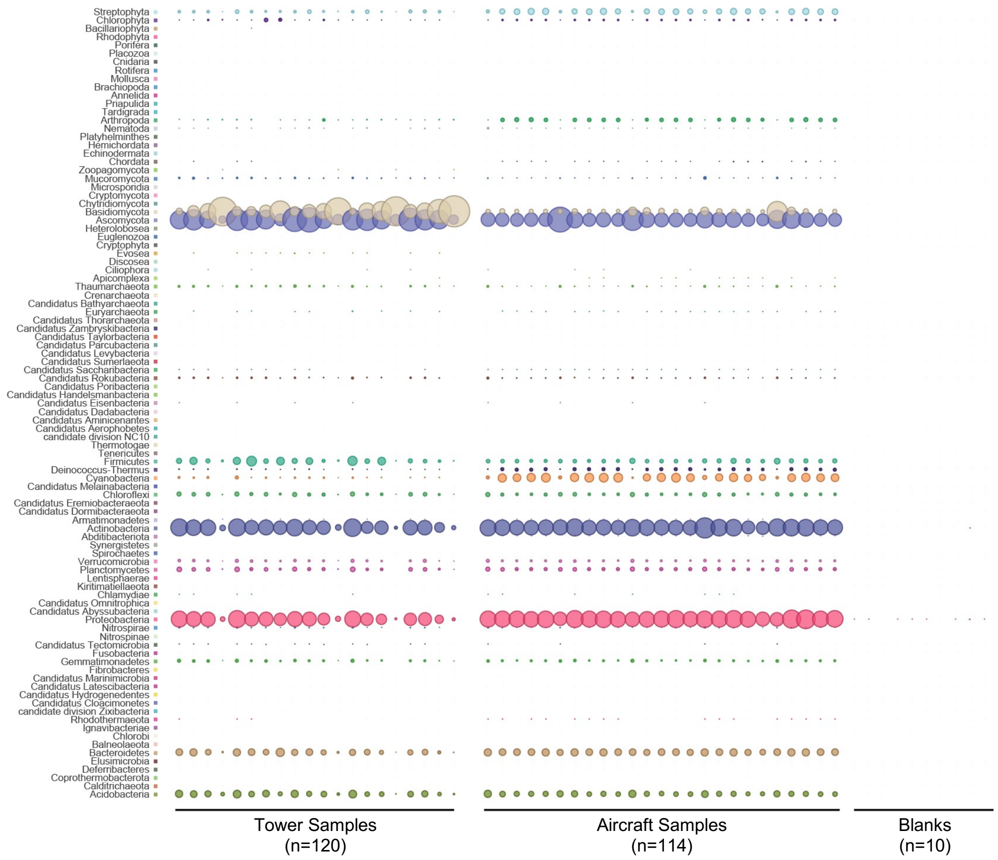

Figure S3. Comparison of taxonomic profile of the air samples from the MT and RA experiments as well as the filter blanks at phylum level. Averages were calculated for day (10am – 8pm) and night (10pm – 8am) samples both at the tower top and bottom for each sampling day (MT experiment). For the RA experiment, for each flight, averages were calculated for the samples of each sampling height.

Overview of Sampling Sites, Study Design & Outcomes

7) Vertical Air Sampling in the Near-Ground Atmosphere (Meteorological Tower)

Near-ground air samples (200m height) were collected at the top of a meteorological tower at the Karlsruhe Institute for Technology (KIT) in Karlsruhe, Germany (Coordinates 49° 5' 33'' N, 8° 25' 33'' E, 110,4m a.s.l). The tower, which has been in operation since 1972, is located in a small forest clearing, surrounded by pine trees, and operated by the Department of Tropospheric Research of the Institute for Meteorology & Climate Research at KIT Campus North (Fig. S4). Sensors at different altitudes of the tower measure a variety of meteorological parameters, including:

- Wind Speed (2, 20, 30, 40, 50, 60, 80, 100, 130, 160, 200m height)
- Wind Direction (40, 60, 80, 100, 160, 200m height)
- Wind Vector (4, 40, 100, 200m height)
- Air Temperature (2, 10, 30, 60, 100, 130, 160, 200m height)
- Dew Point (2, 10, 30, 100, 200m height)
- Short and long wave radiation components
- Soil heat flux
- Precipitation
- Air pressure

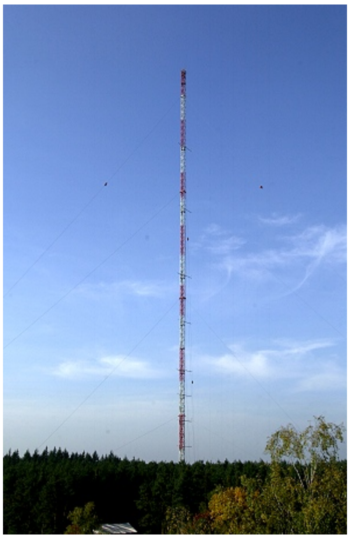

Figure S4. Overview of meteorological tower sampling site. Source: www.imk-tro.kit.edu

7.1) Pilot 1: Air sampling at the meteorological tower in February 2018

An initial pilot experiment was conducted on 27 February 2018 to evaluate the feasibility of using the meteorological tower in Karlsruhe for a future, large-scale sampling campaign. For this pilot, SASS3100 air samplers were placed on the uppermost platform of the tower (200m) as well as at ground level at the base of the tower (~0.75m). Air samples were collected in duplicates at noon and midnight for a sampling duration of 2 hours with a flowrate of 300L/min at both sampling locations (tower top and bottom). For the 200m noon samples as well as for all midnight samples, low DNA amounts were obtained from single filters and the extracted DNA from the duplicates was subsequently pooled for metagenomic sequencing (Figs. S5 – S9).

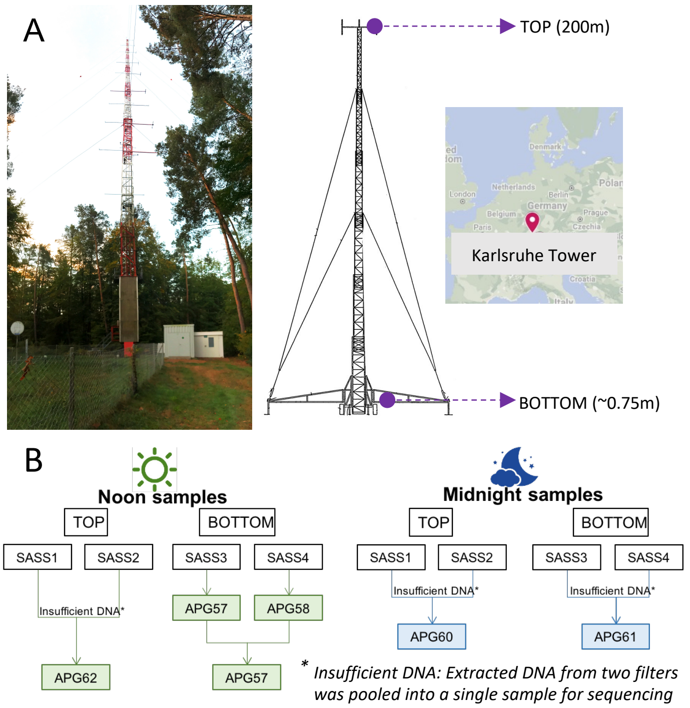

Figure S5. Pilot experiment at the meteorological tower in February 2018.
A) Overview of sampling site.
B) Overview of experimental set-up and sample processing.

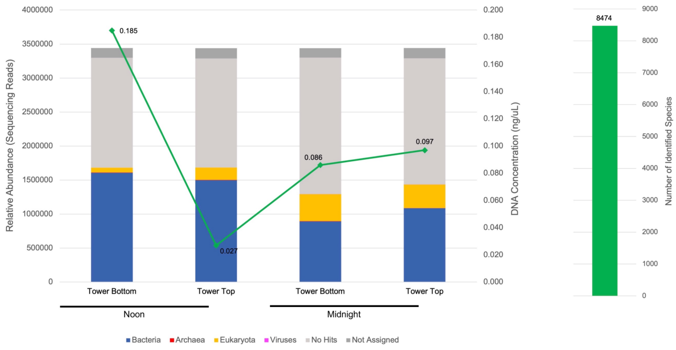

Figure S6. Taxonomic overview for samples collected at the meteorological tower in February 2018. Relative abundances at superkingdom-level are based on number of assigned sequencing reads; DNA concentrations of analyzed samples and total number of identified species (averages per time point and sampling location).

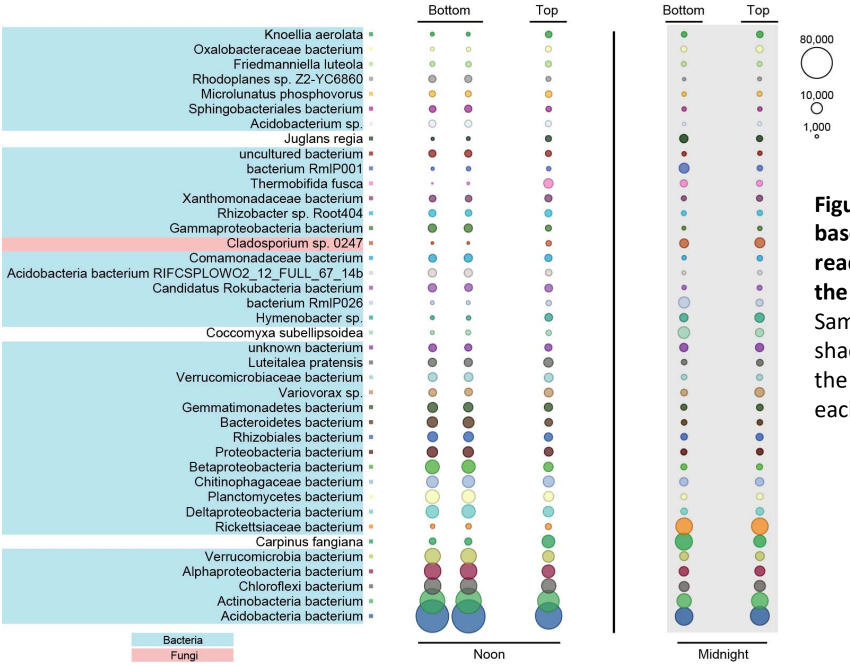

Figure S7. Top 40 most abundant species based on number of assigned sequencing reads identified in air samples collected at the meteorological tower in February 2018. Samples collected during night hours are shaded in grey. Bubble size is proportional to the number of sequencing reads assigned to each taxon.

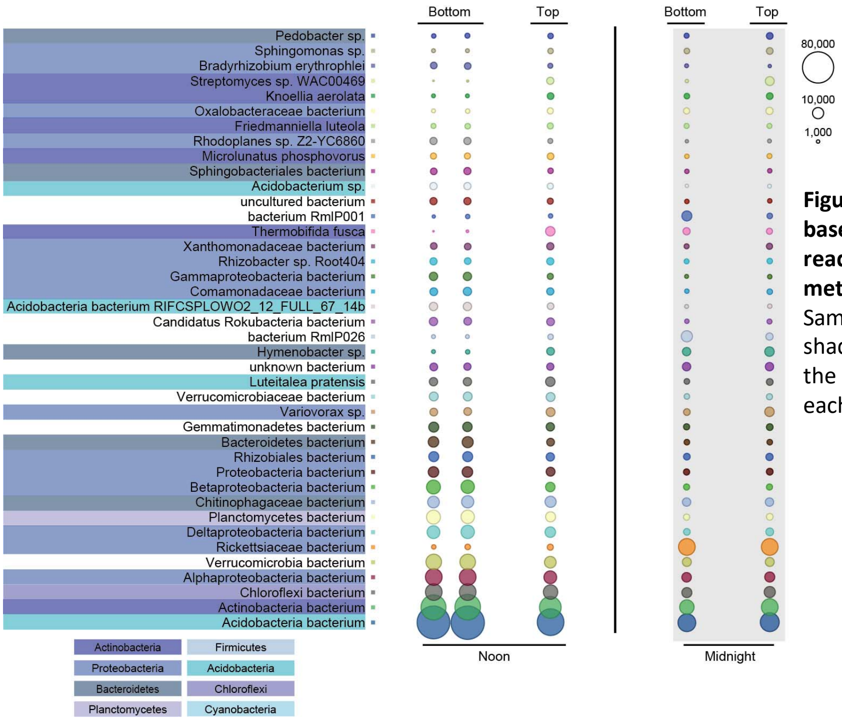

Figure S8. Top 40 most abundant bacteria based on number of assigned sequencing reads identified in air samples collected at the meteorological tower in February 2018. Samples collected during night hours are shaded in grey. Bubble size is proportional to the number of sequencing reads assigned to each taxon.

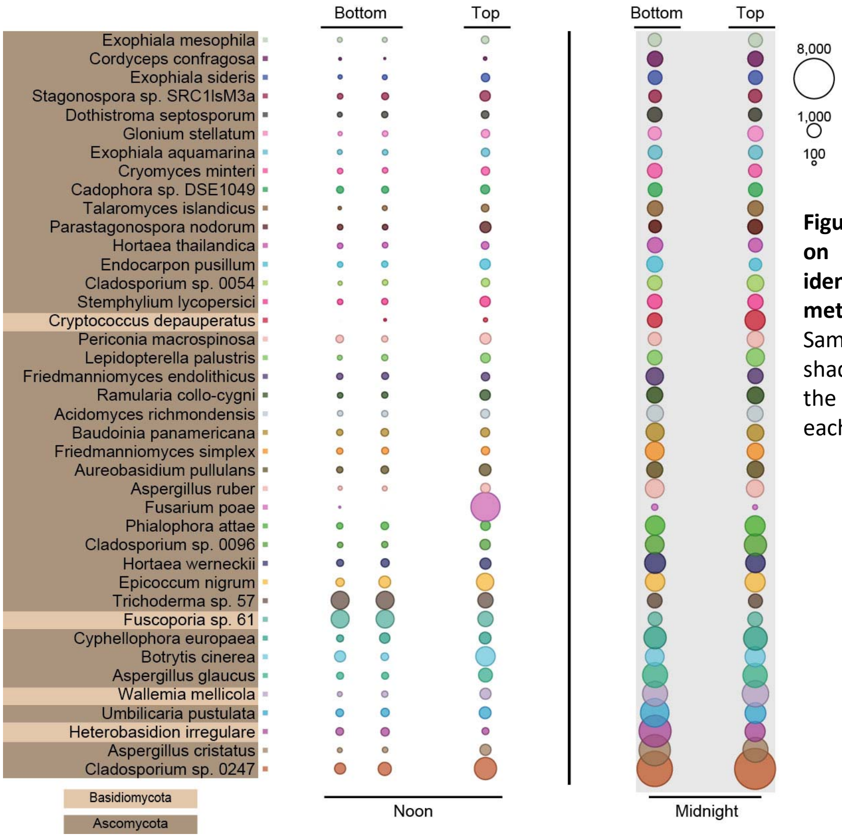

Figure S9. Top 40 most abundant fungi based on number of assigned sequencing reads identified in air samples collected at the meteorological tower in February 2018. Samples collected during night hours are shaded in grey. Bubble size is proportional to the number of sequencing reads assigned to each taxon.

7.2) Pilot 2: Air sampling at the meteorological tower in July 2018

A second pilot experiment was conducted in July 2018 during the summer period in Germany to investigate seasonal influences on the air microbiome composition. For this study, air samples were also collected at the top of the tower (200m) and near its base (~0.75m) at noon and midnight for a sampling duration of 2 hours with a flowrate of 300L/min. Samples were collected for two consecutive days on July 9 and 10 (Figs. S10 – S13).

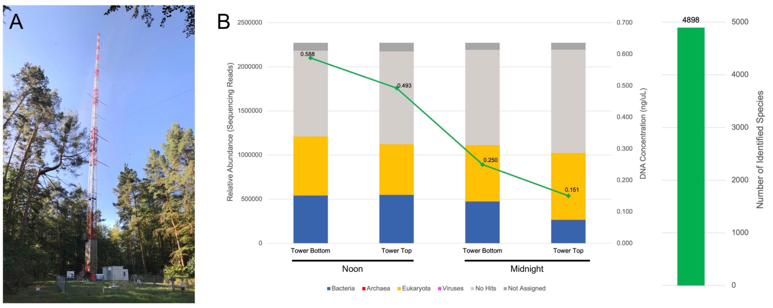

Figure S10. Taxonomic overview for samples collected at the meteorological tower in June 2018.
Relative abundances at superkingdom-level are based on number of assigned sequencing reads; DNA concentrations of analyzed samples and total number of identified species (averages per time point and sampling location).

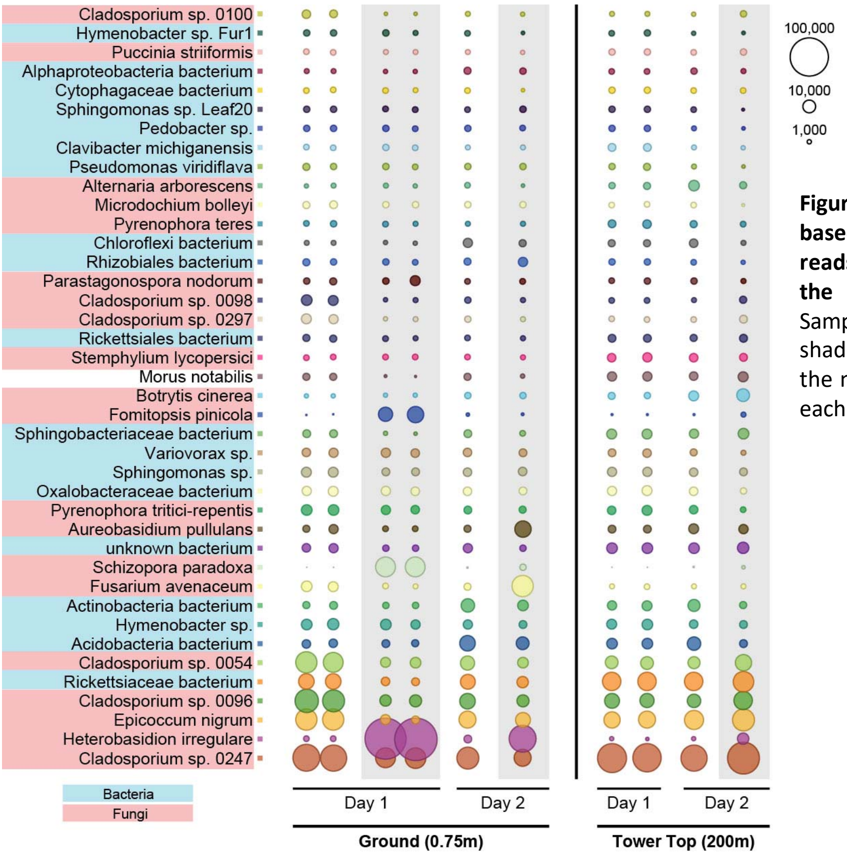

Figure S11. Top 40 most abundant species based on number of assigned sequencing reads identified in air samples collected at the meteorological tower in July 2018. Samples collected during night hours are shaded in grey. Bubble size is proportional to the number of sequencing reads assigned to each taxon.

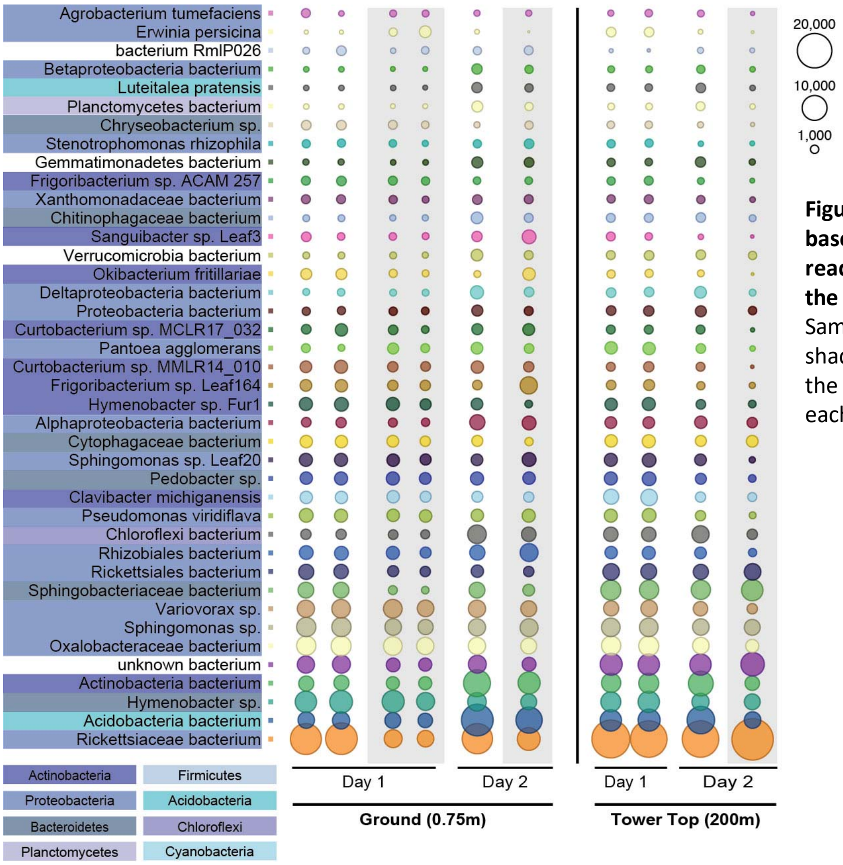

Figure S12. Top 40 most abundant bacteria based on number of assigned sequencing reads identified in air samples collected at the meteorological tower in July 2018. Samples collected during night hours are shaded in grey. Bubble size is proportional to the number of sequencing reads assigned to each taxon.

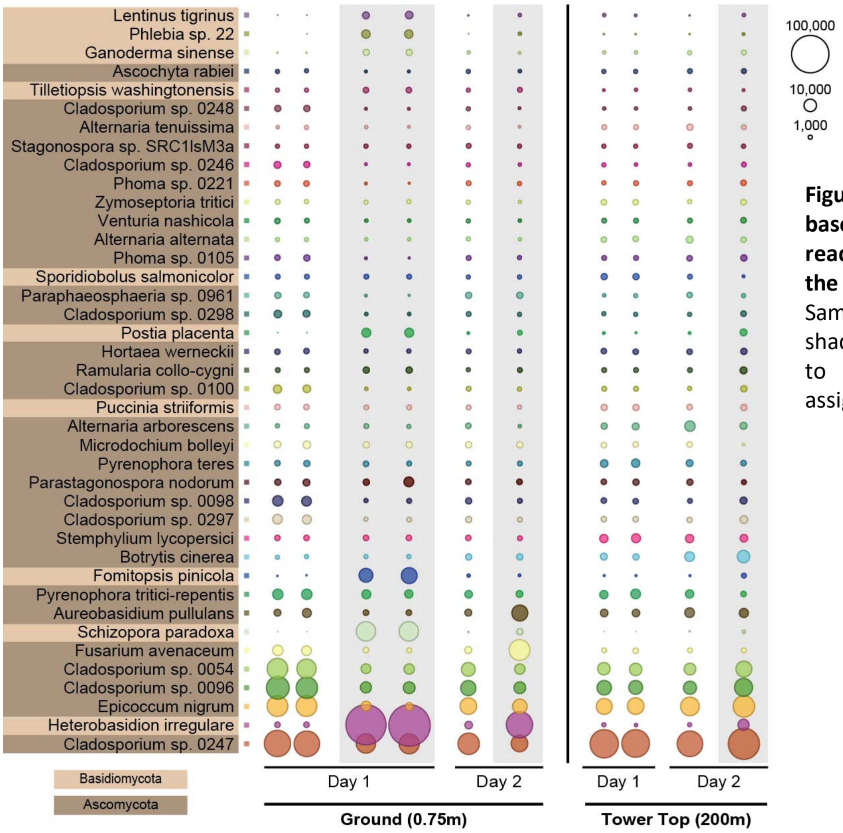

Figure S13. Top 40 most abundant fungi based on number of assigned sequencing reads identified in air samples collected at the meteorological tower in July 2018. Samples collected during night hours are shaded in grey. Bubble size is proportional to the number of sequencing reads assigned to each taxon.

7.3) Summer/winter seasonal comparison between samples collected at the meteorological tower in Karlsruhe, Germany, in February and July 2018

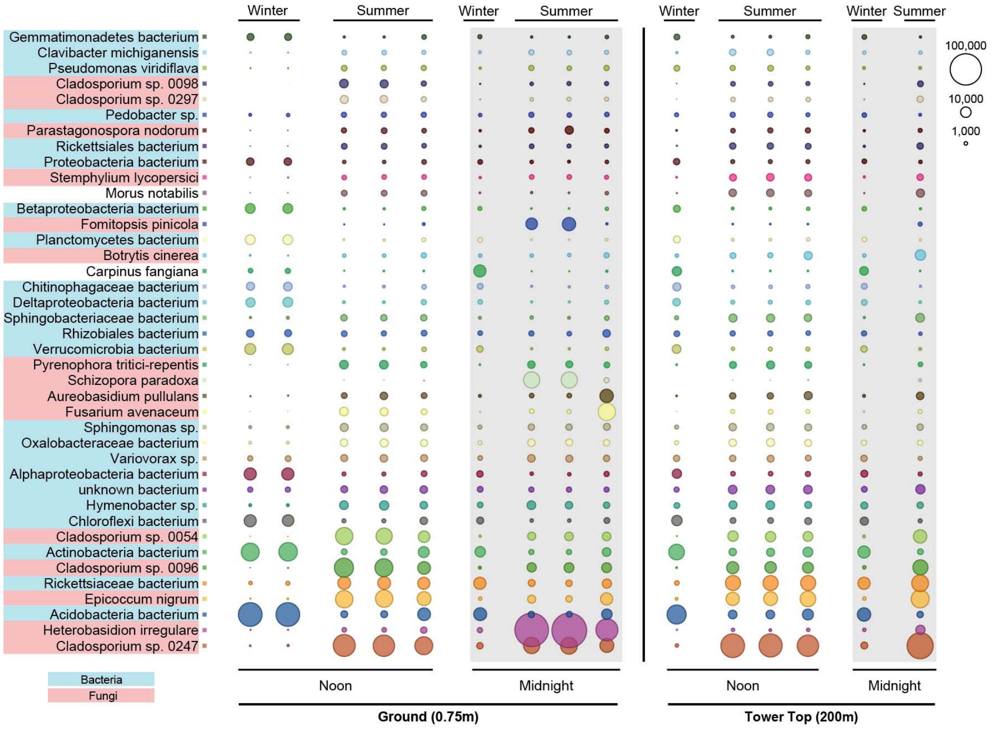

Figure S14. Summer/winter seasonal comparison. Top 40 most abundant species based on number of assigned sequencing reads identified in air samples collected at the meteorological tower in February and July 2018, respectively. Samples collected during night hours are shaded in grey. Bubble size is proportional to the number of sequencing reads assigned to each taxon.

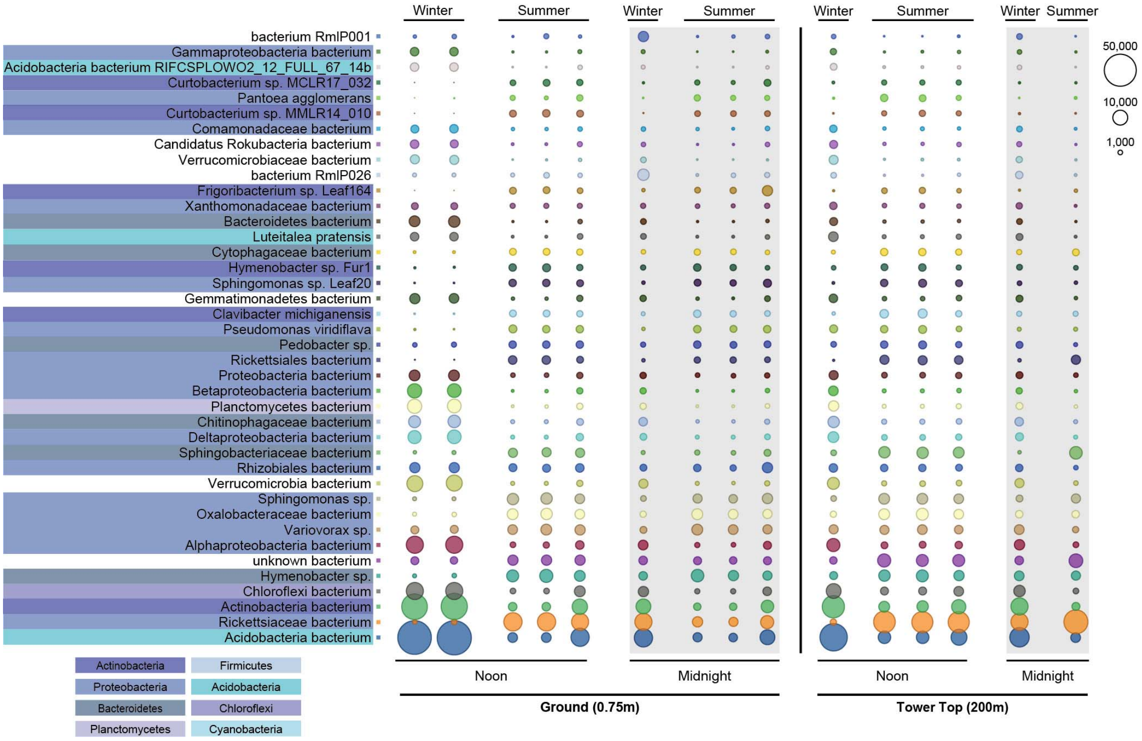

Figure S15. Summer/winter seasonal comparison. Top 40 most abundant bacterial species based on number of assigned sequencing reads identified in air samples collected at the meteorological tower in February and July 2018, respectively. Samples collected during night hours are shaded in grey. Bubble size is proportional to the number of sequencing reads assigned to each taxon.

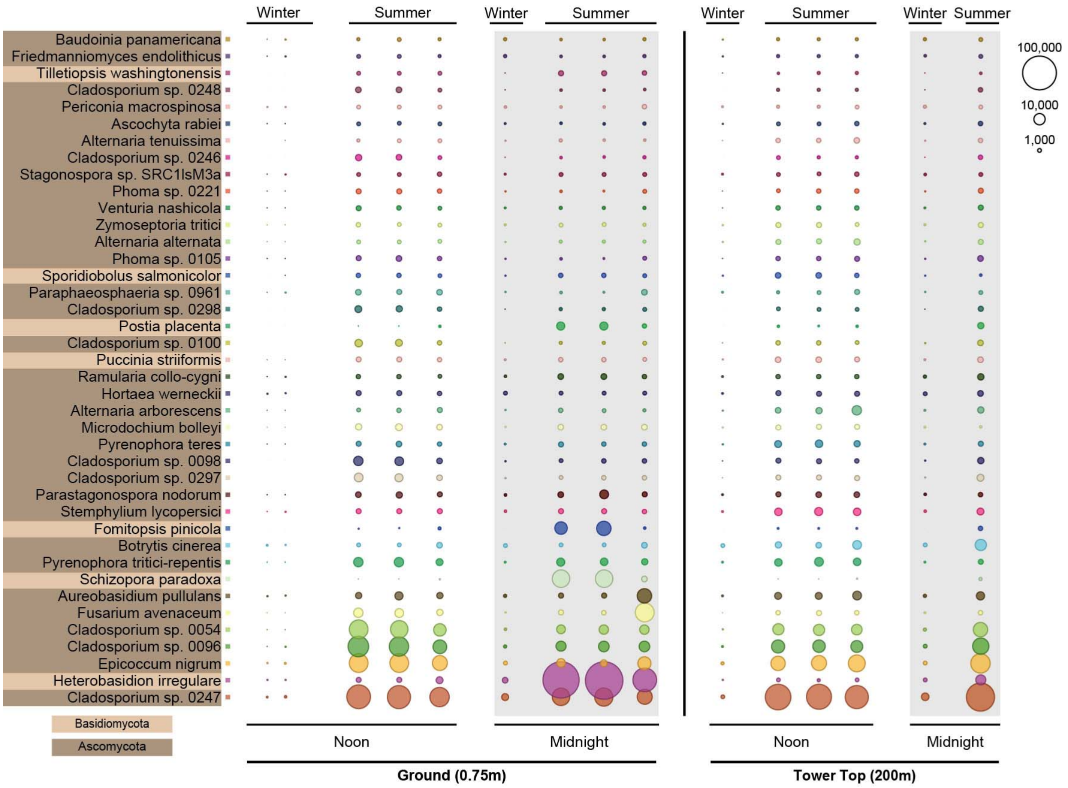

Figure S16. Summer/winter seasonal comparison. Top 40 most abundant fungal species based on number of assigned sequencing reads identified in air samples collected at the meteorological tower in February and July 2018, respectively. Samples collected during night hours are shaded in grey. Bubble size is proportional to the number of sequencing reads assigned to each taxon.

7.4) Large-scale study to investigate temporal and vertical distribution of airborne microbial organisms in the near-surface atmosphere (October 2018)

A total of 16 SASS air samplers (8 air samplers per location) were placed on the uppermost platform of the tower at 200m height as well as near the base of the tower, approx. 0.75m above the ground. The instruments were programmed with delays so that always 2 instruments at the top and 2 instruments at the bottom were running simultaneously at the following time points without the need for human supervision: 10am-12pm, 2-4pm, 6-8pm 10pm-12am, 2-4am, 6-8am. Sample collection therefore occurred in duplicates for a duration of 2 hours per time point every 4 hours (6 time points per day) and was repeated for 5 consecutive days (8 Oct 2018, 10am until 13 Oct 2018, 8am). The sampling days in October were chosen as large high pressure system with stable weather conditions was predicted for most of Central Europe for the entire week, enabling sampling conditions as consistent as possible across all sampling days (Figs. S17 – S31).

In addition to the air samples, one blank sample from a randomly selected SASS instrument was collected at the tower top and at the ground every day, resulting in a total of 130 collected filters for the entire experiment (120 samples and 10 blanks).

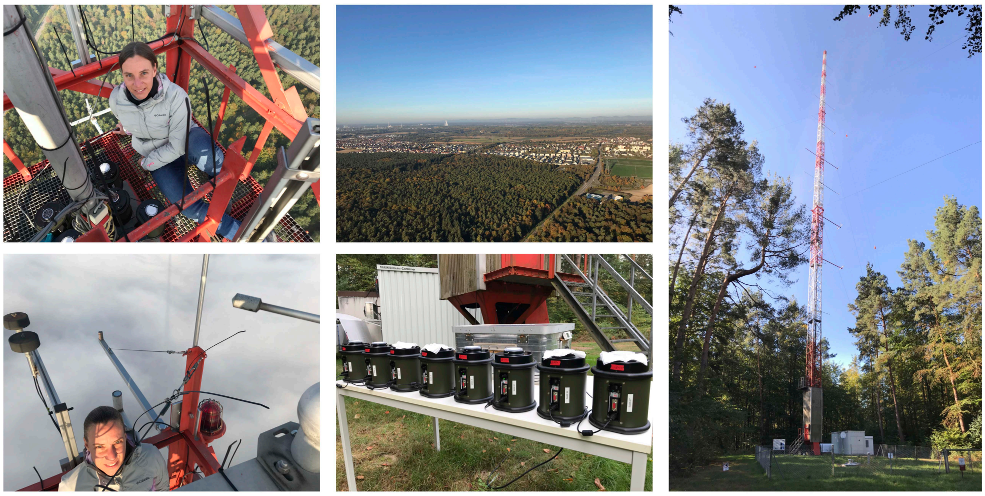

Figure S17. Temporal and vertical distribution of airborne microbial organisms in the near-surface atmosphere. Experimental set-up. SASS air samplers were placed on the uppermost platform of the tower (200m), as well as near the base of the tower (~0.75m). Air samples were collected at the top and bottom for 5 consecutive days, in 4-hour increments, and a sampling duration of 2 hours. For each time point, samples were collected in duplicates.

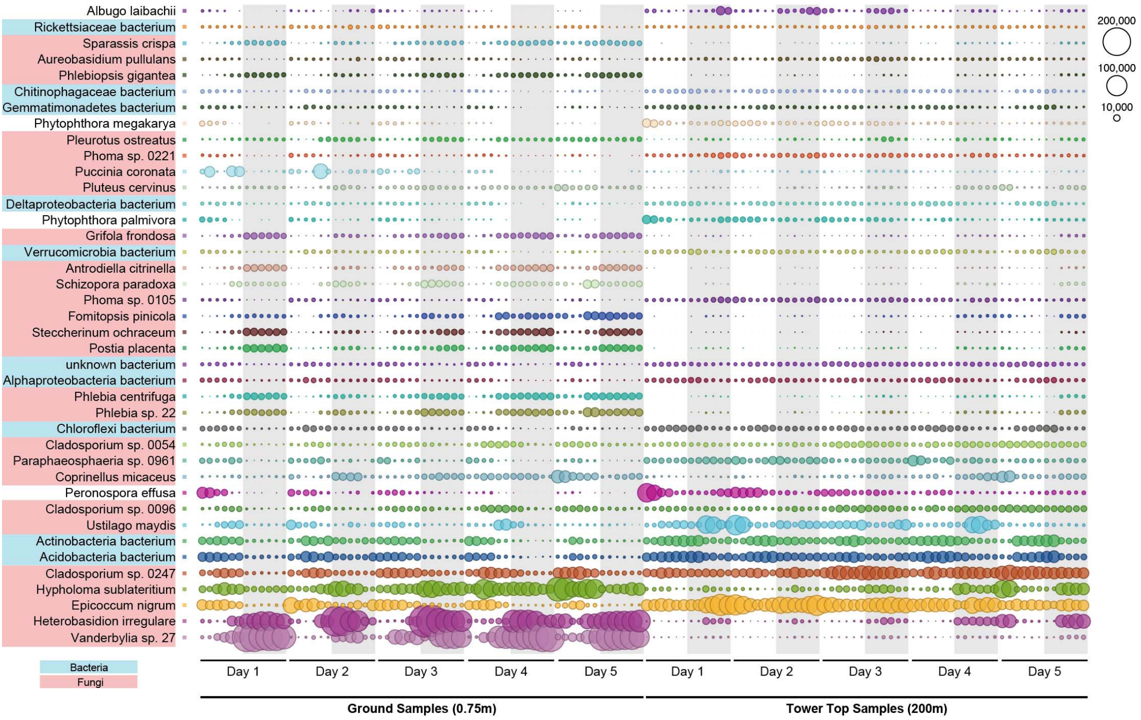

Figure S18. Diel cycle of airborne microbial communities in the temperate climate, Top 40 all species. Temporal (5 consecutive days) and vertical distribution (0-200m) of the top 40 most abundant species based on number of assigned sequencing reads identified in air samples collected at the meteorological tower in October 2018. Ground samples grouped together and sorted by sampling time and day, followed by the 200m (tower top) samples. Samples collected during night hours are shaded in grey. Bubble size is proportional to the number of sequencing reads assigned to each taxon.

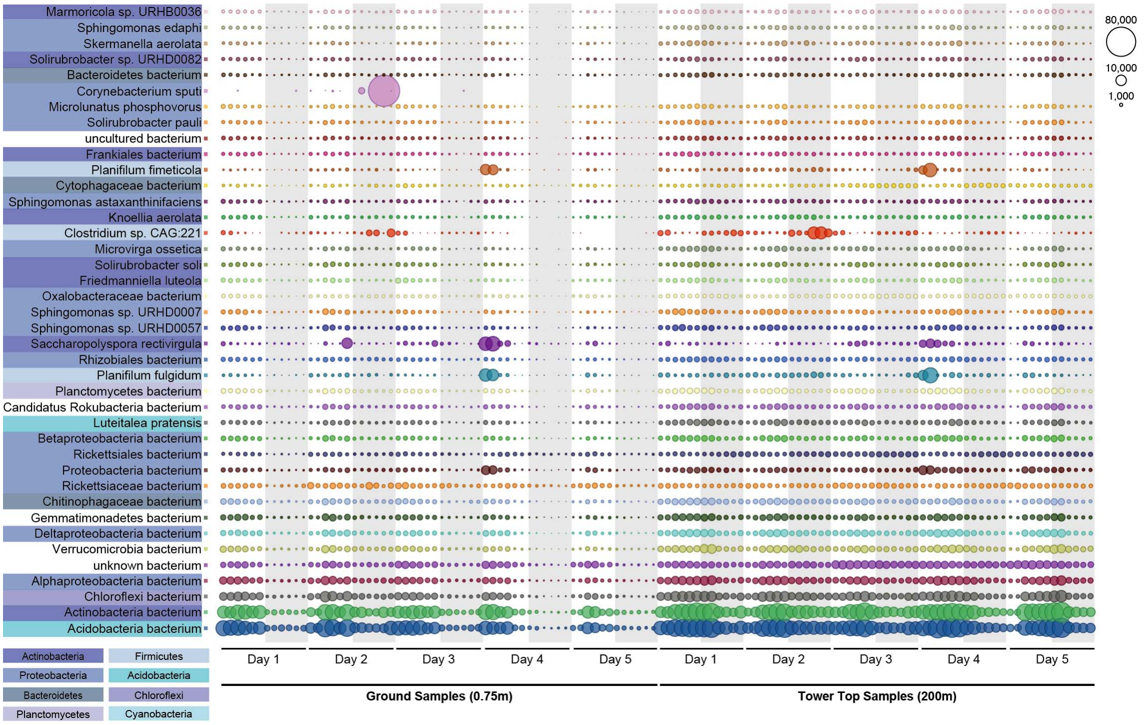

Figure S19. Diel cycle of airborne microbial communities in the temperate climate; Top 40 bacteria. Temporal (5 consecutive days) and vertical distribution (0-200m) of the top 40 most abundant bacteria based on number of assigned sequencing reads identified in air samples collected at the meteorological tower in October 2018. Ground samples grouped together and sorted by sampling time and day, followed by the 200m (tower top) samples. Samples collected during night hours are shaded in grey. Bubble size is proportional to the number of sequencing reads assigned to each taxon.

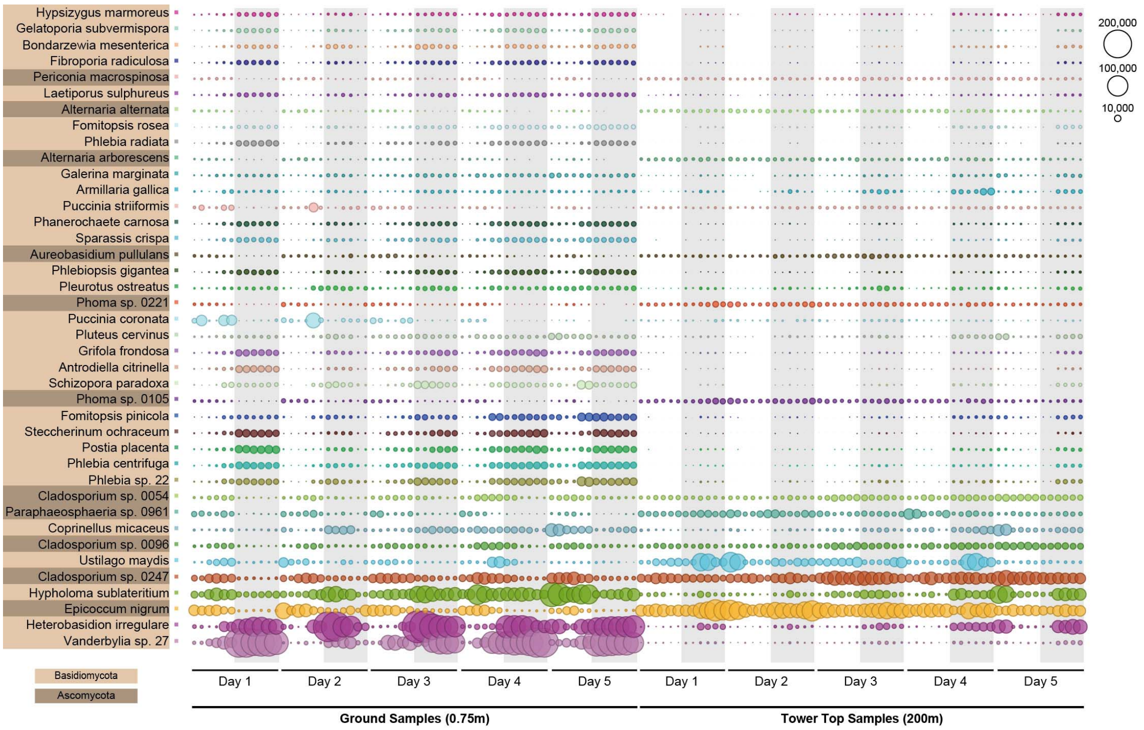

Figure S20. Diel cycle of airborne microbial communities in the temperate climate; Top 40 fungi. Temporal (5 consecutive days) and vertical distribution (0-200m) of the top 40 most abundant fungi based on number of assigned sequencing reads identified in air samples collected at the meteorological tower in October 2018. Ground samples grouped together and sorted by sampling time and day, followed by the 200m (tower top) samples. Samples collected during night hours are shaded in grey. Bubble size is proportional to the number of sequencing reads assigned to each taxon.

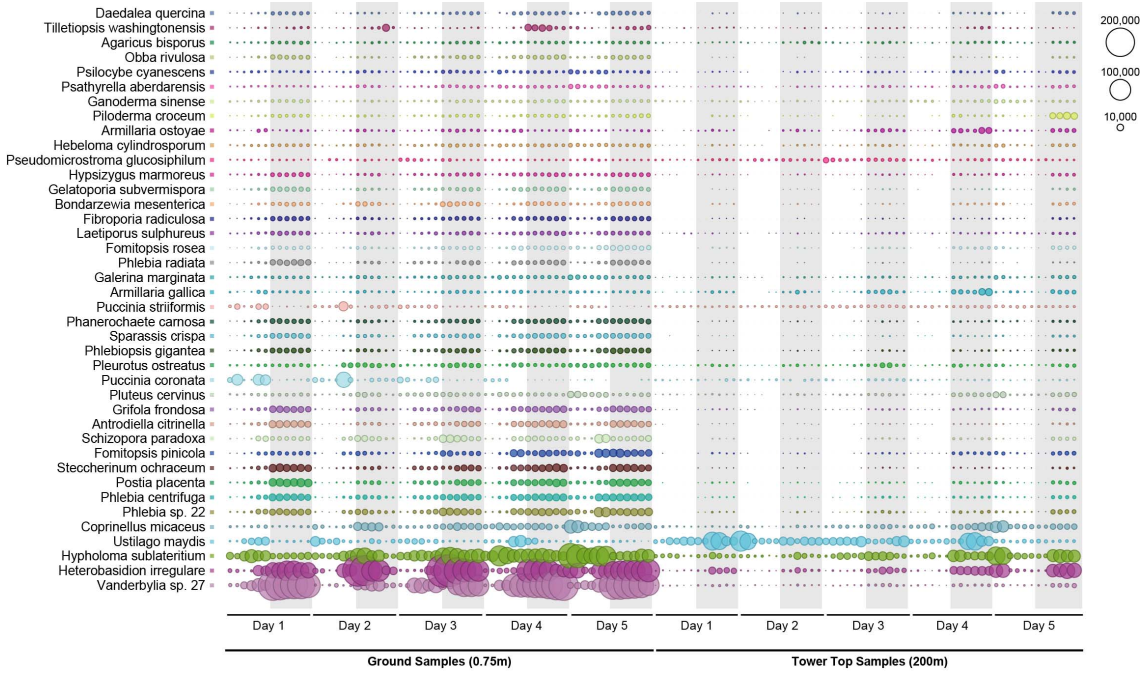

Figure S21. Diel cycle of airborne microbial communities in the temperate climate; Top 40 basidiomycota. Temporal (5 consecutive days) and vertical distribution (0-200m) of the top 40 most abundant basidiomycota fungi based on number of assigned sequencing reads identified in air samples collected at the meteorological tower in October 2018. Ground samples grouped together and sorted by sampling time and day, followed by the 200m (tower top) samples. Samples collected during night hours are shaded in grey. Bubble size is proportional to the number of sequencing reads assigned to each taxon.

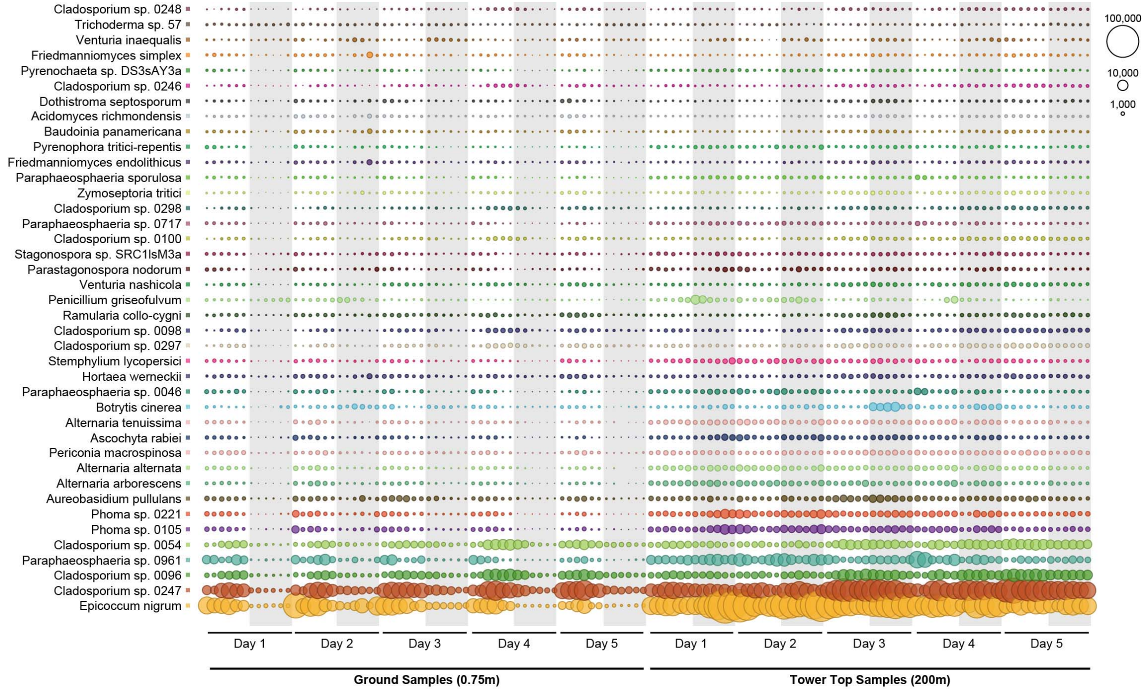

Figure S22. Diel cycle of airborne microbial communities in the temperate climate; Top 40 ascomycota. Temporal (5 consecutive days) and vertical distribution (0-200m) of the top 40 most abundant ascomycota fungi based on number of assigned sequencing reads identified in air samples collected at the meteorological tower in October 2018. Ground samples grouped together and sorted by sampling time and day, followed by the 200m (tower top) samples. Samples collected during night hours are shaded in grey. Bubble size is proportional to the number of sequencing reads assigned to each taxon.

7.5) Meteorological tower, October 2018 study; comparison of 200m samples only

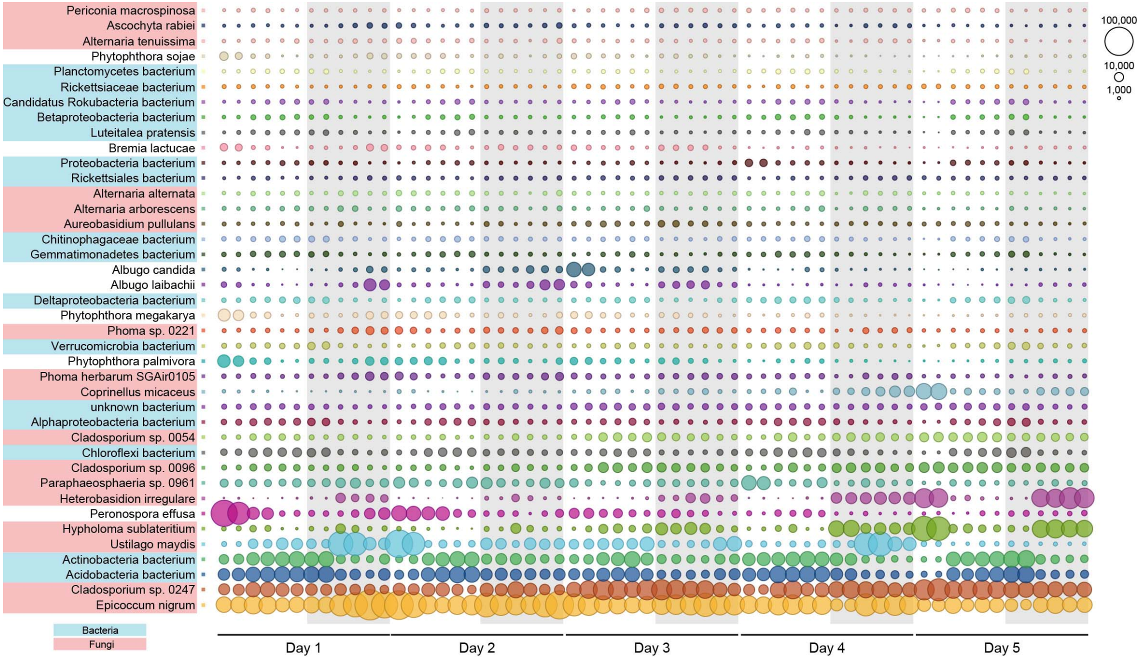

Figure S23. Comparison of 200m samples only; Top 40 all species. Temporal (5 consecutive days) distribution of the top 40 most abundant species based on number of assigned sequencing reads identified in the 200m tower top only samples collected at the meteorological tower in October 2018. The samples are grouped by sampling day and sorted by sampling time. Samples collected during night hours are shaded in grey. Bubble size is proportional to the number of sequencing reads assigned to each taxon.

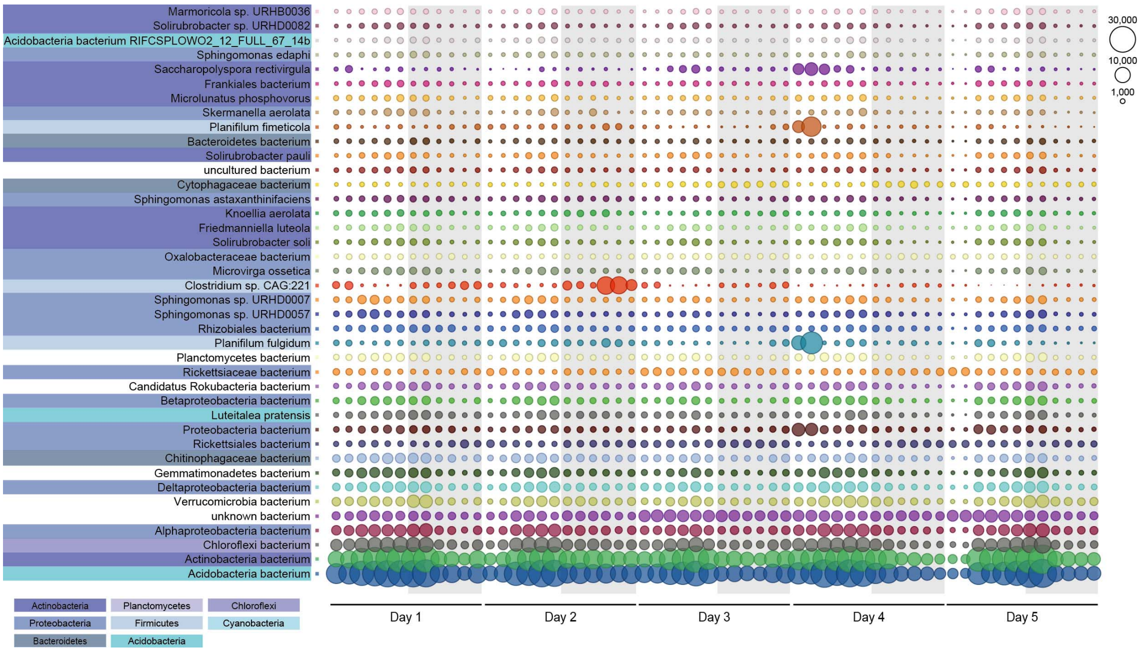

Figure S24. Comparison of 200m samples only; Top 40 bacteria. Temporal (5 consecutive days) distribution of the top 40 most abundant bacteria based on number of assigned sequencing reads identified in the 200m tower top only samples collected at the meteorological tower in October 2018. The samples are grouped by sampling day and sorted by sampling time. Samples collected during night hours are shaded in grey. Bubble size is proportional to the number of sequencing reads assigned to each taxon.

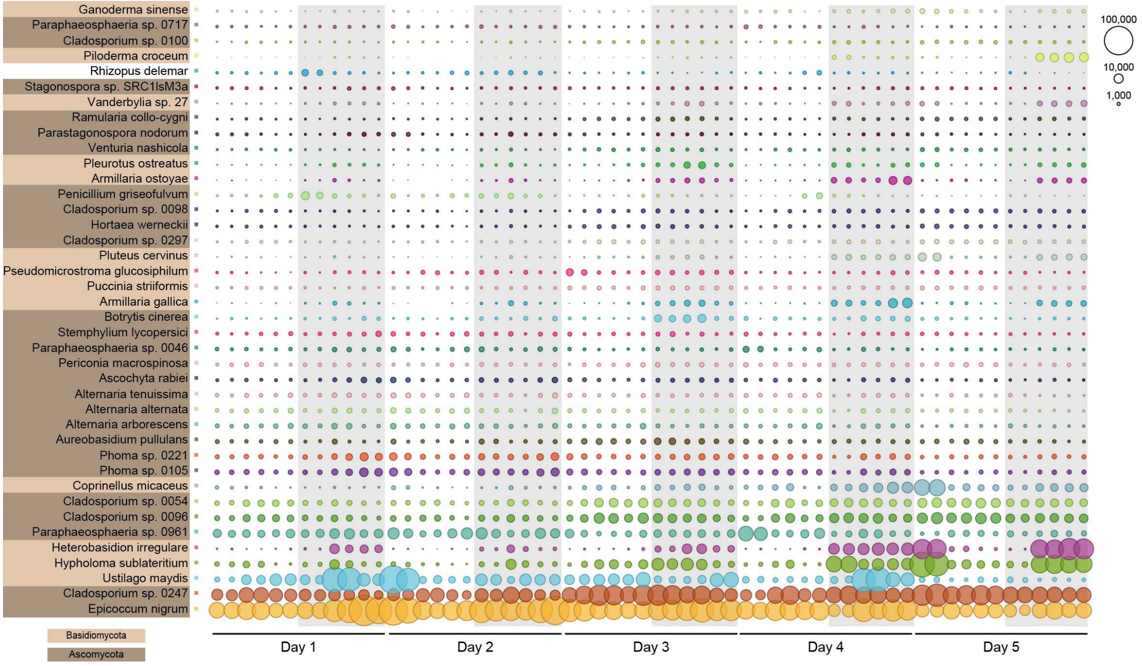

Figure S25. Comparison of 200m samples only; Top 40 fungi. Temporal (5 consecutive days) distribution of the top 40 most abundant fungi based on number of assigned sequencing reads identified in the 200m tower top only samples collected at the meteorological tower in October 2018. The samples are grouped by sampling day and sorted by sampling time. Samples collected during night hours are shaded in grey. Bubble size is proportional to the number of sequencing reads assigned to each taxon.

7.6) Meteorological tower, October 2018 study; comparison of ground samples only

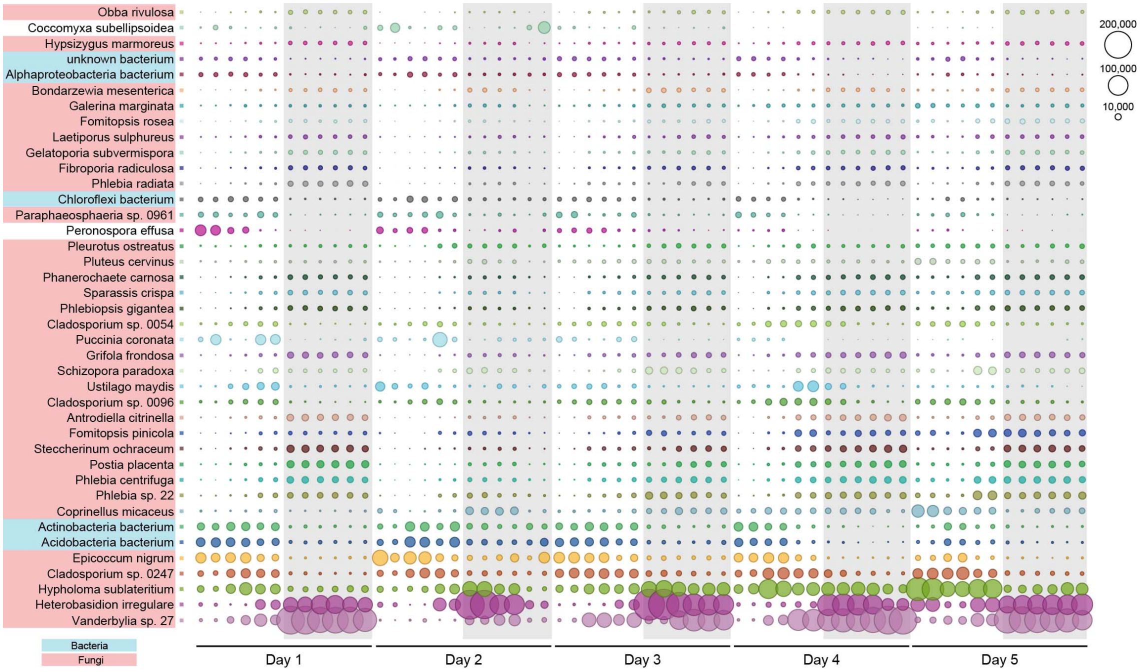

Figure S26. Comparison of ground samples only; Top 40 all species. Temporal (5 consecutive days) distribution of the top 40 most abundant species based on number of assigned sequencing reads identified in the ground only samples collected at the meteorological tower in October 2018. The samples are grouped by sampling day and sorted by sampling time. Samples collected during night hours are shaded in grey. Bubble size is proportional to the number of sequencing reads assigned to each taxon.

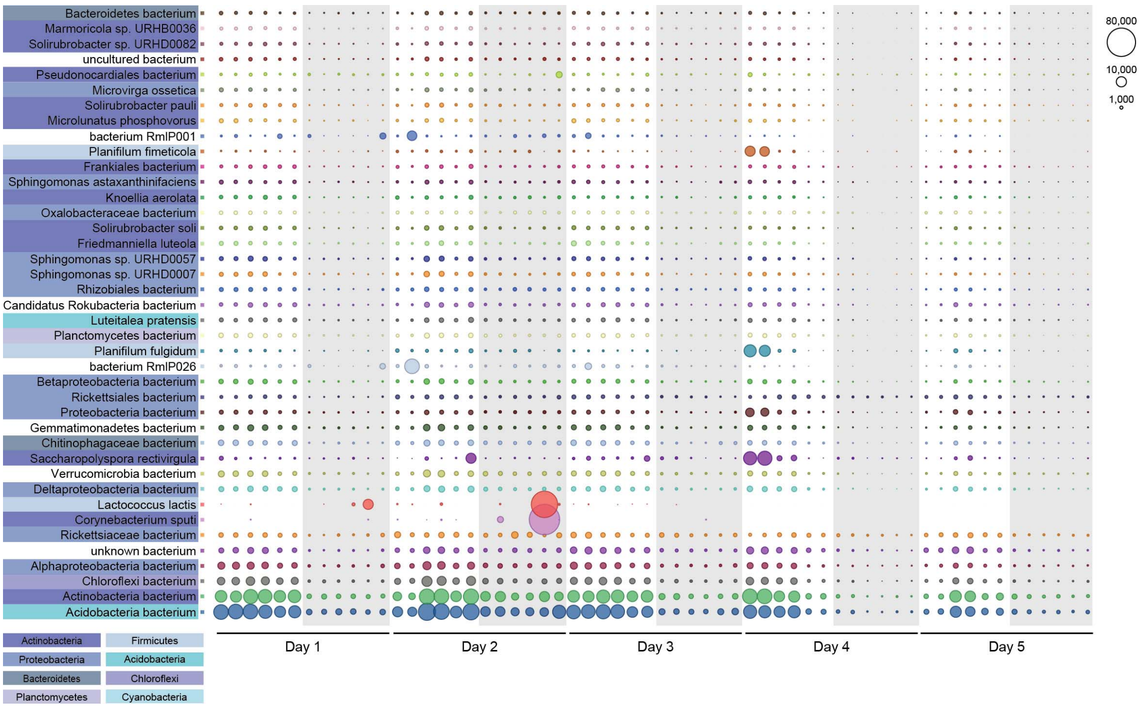

Figure S27. Comparison of ground samples only; Top 40 bacteria. Temporal (5 consecutive days) distribution of the top 40 most abundant bacteria based on number of assigned sequencing reads identified in the ground only samples collected at the meteorological tower in October 2018. The samples are grouped by sampling day and sorted by sampling time. Samples collected during night hours are shaded in grey. Bubble size is proportional to the number of sequencing reads assigned to each taxon.

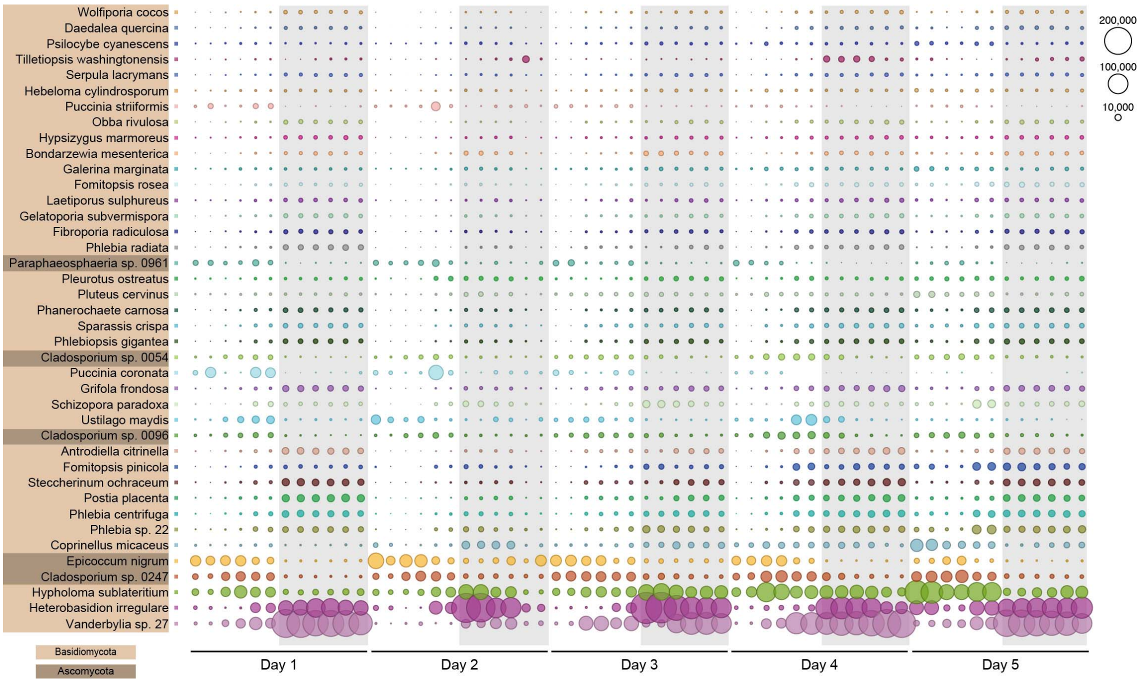

Figure S28. Comparison of ground samples only; Top 40 fungi. Temporal (5 consecutive days) distribution of the top 40 most abundant fungi based on number of assigned sequencing reads identified in the ground only samples collected at the meteorological tower in October 2018. The samples are grouped by sampling day and sorted by sampling time. Samples collected during night hours are shaded in grey. Bubble size is proportional to the number of sequencing reads assigned to each taxon.

7.7) Using horizontal wind speed measurements as proxy for vertical mixing processes within the atmospheric boundary layer at the meteorological tower for the experiment conducted in October 2018

At the meteorological tower the measuring height is restricted to 200m a.g.l. This height limitation prevents the detection of the daytime mixing layer height (MLH), however, the development of the night-time stable layer with suppression on vertical mixing can be observed. Horizontal wind speed measurements at the tower were used to calculate vertical

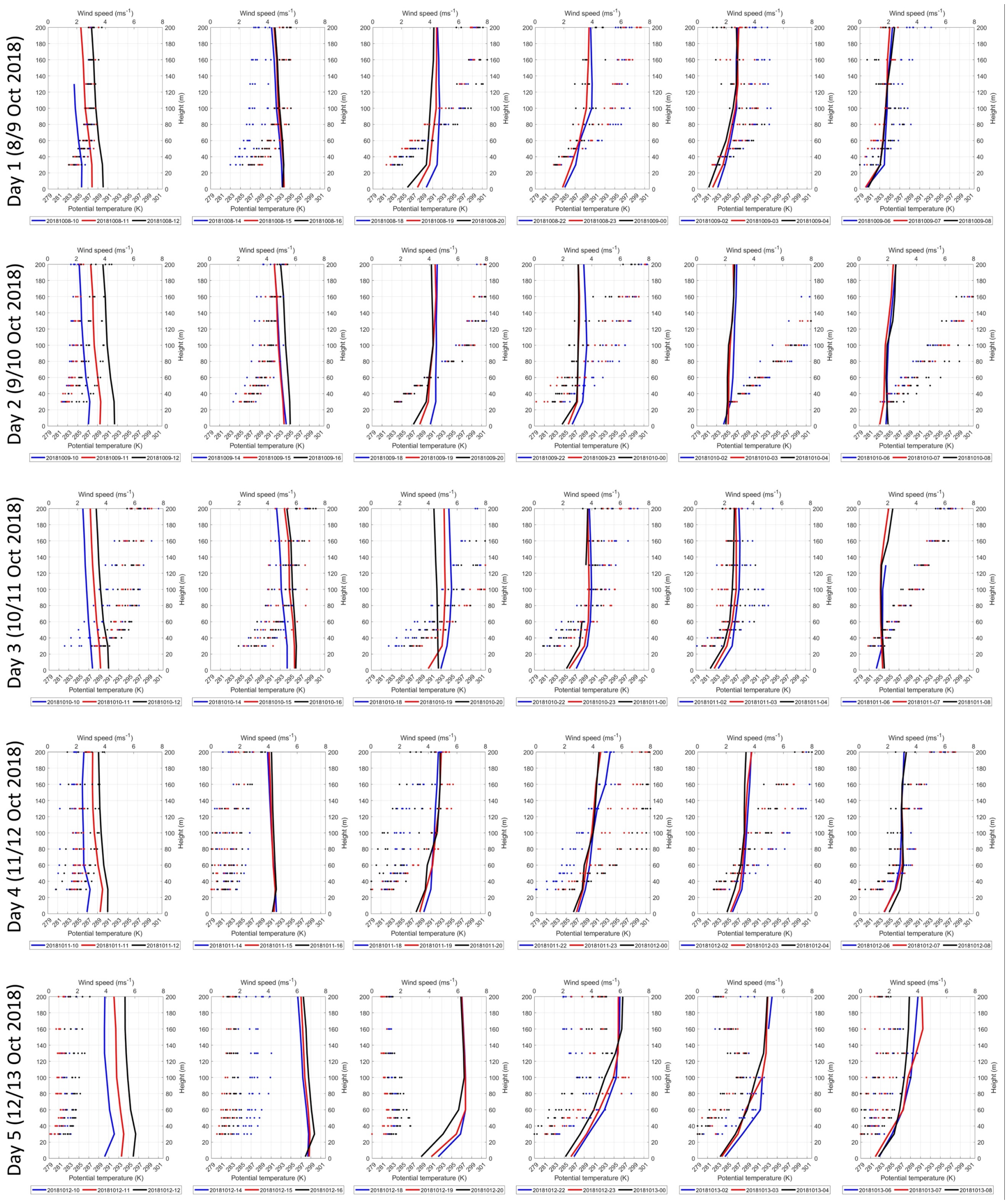

Figure S29. Horizontal wind speed measurements as proxy for vertical mixing processes within the atmospheric boundary layer. Each subfigure represents three profiles of potential temperature (blue, red and black lines) with 10-minute mean values at the full hour as indicated in the legend. Ten-minute mean data of the horizontal wind speed for each hour are depicted as dots for each measured height (30m, 40m, 50m, 60m, 80m, 100m, 130m, 160m, 200m), color-coded with the corresponding profile of the potential temperature.

wind speed to provide better insights into the level of vertical mass transport during the day and night at the tower location.
As vertical wind speed measurements are not recorded at the tower, this lack of information can be overcome by using horizontal wind speed and it's variability throughout the sampling period in the different measuring heights at the tower as a proxy for vertical motion within the atmospheric boundary layer (ABL). The measured 10-minute averages of horizontal wind speed are a valuable indicator for the degree of turbulence within the ABL. The higher the mean horizontal wind speed, the higher the three dimensional turbulence and vertical motion (positive or negative), and as a result, higher vertical mixing within the ABL is expected.

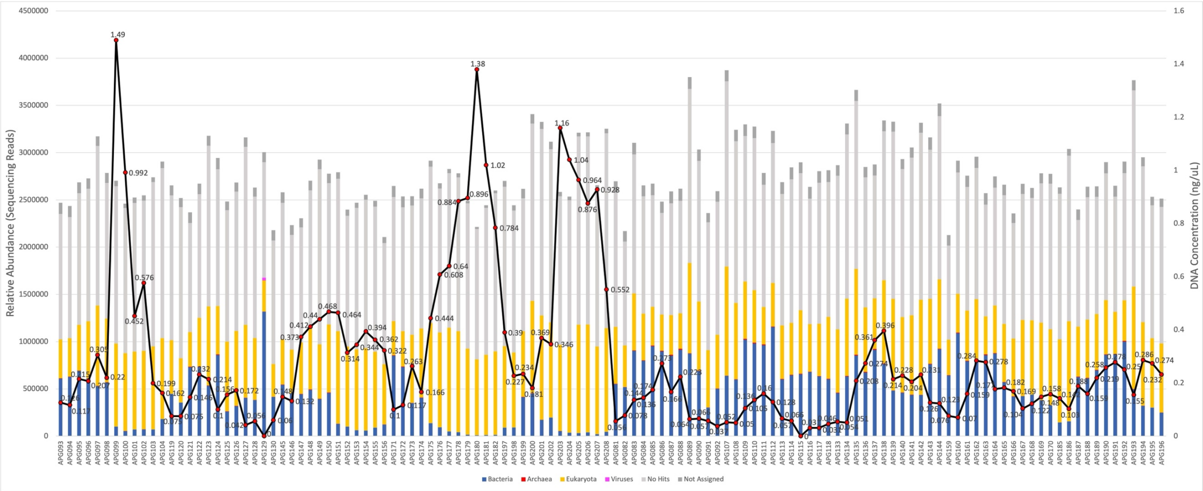

Figure S30. Domain-level comparison between all samples from the October 2018 meteorological tower (MT) experiment (tower top and ground samples). Relative abundances are shown based on absolute number of sequencing reads obtained for each sample. Also shown are the obtained DNA concentrations for each individual sample. Ground samples grouped together and sorted by sampling time and day, followed by the 200m (tower top) samples.

7.8) <u>Distribution of specific fungal species at different heights in the MT experiment</u>

The higher abundance of fungi in MT air samples allowed us to study the distribution of different classes of fungi at different times of the day as well as heights. Basidiomycota abundance, particularly for the listed species in Figure S30 that belong to the polyporales order, is observed to be the highest at ground level, with strong increases during night-time while declining during daytime. A strongly diminished signal was recorded for this taxonomic group at 200m height, indicating the suitability of this group of species to be considered as a proxy for ground-based taxa. This finding is expected as Basidiomycota species are largely known to be wood-rotting fungi (Fig. S30a). Inversely, the listed species from its fungal sister clade Ascomycota peaked during solar noon on ground level and showed a low abundance during the night. At 200m height, a constant high level of Ascomycota taxa was observed, largely independent of the diel cycle (Fig. S30b). This distribution pattern is more similar to that of the bacterial taxa which are mostly associated with higher heights. While airborne Ascomycetes spores are widely regarded to originate from ground sources, this finding indicates that they may possess higher tendency to remain airborne for a longer time than its Basidiomycota counterparts.

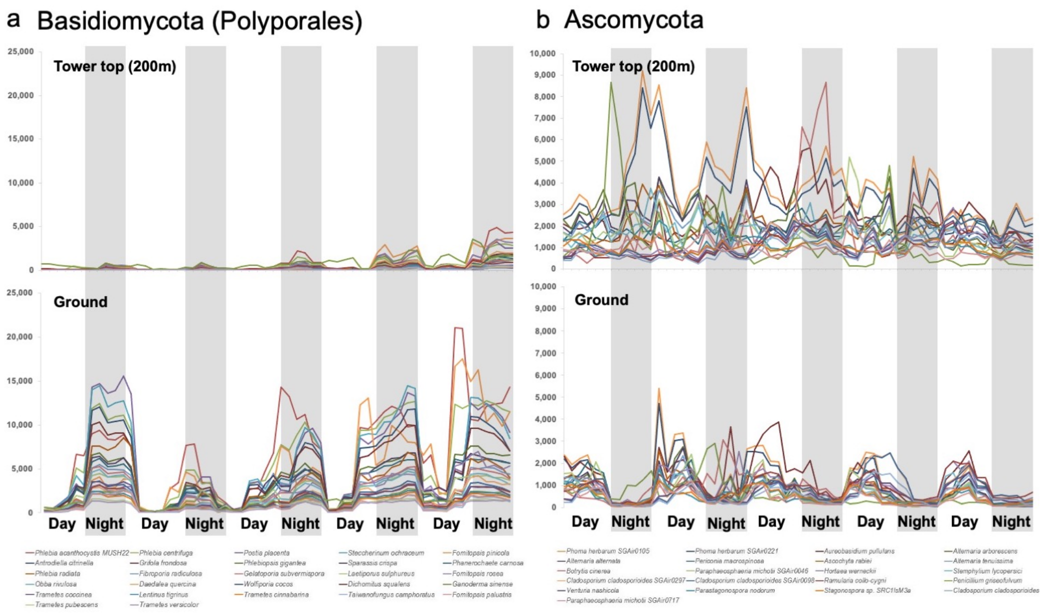

Figure S31. Relative abundance of 24 Basidiomycota and Ascomycota species at different heights of the MT experiment.

7.9) <u>Seasonal variability at the meteorological tower: Winter-summer-fall comparison:</u>

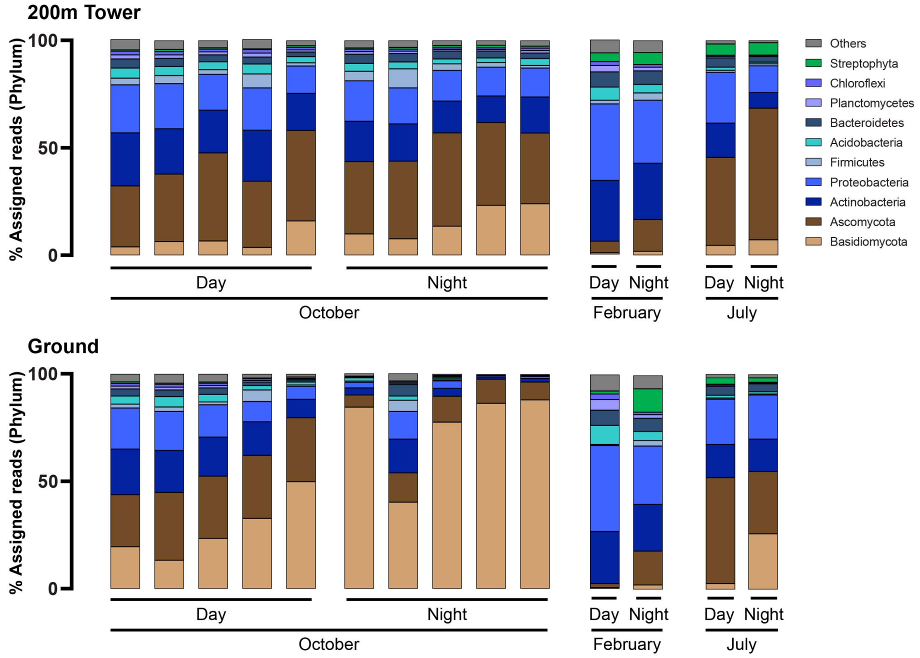

Figure S32. Seasonal variability. Phylum-level comparison between samples collected during winter (February 2018), summer (July 2018) and fall (October 2018) at the tower top (200m) and ground of the meteorological tower in Karlsruhe, Germany.

7.10) <u>Comparison of diel variations in air microbiome composition for samples collected in the temperate climate zone (Karlsruhe, Germany) and in the tropics (Singapore)</u>

Germany samples were collected at the ground and 200m top of the meteorological tower in Karlsruhe whereas Singapore samples were collected at an open balcony at Nanyang Technological University. This dataset was analysed with the same analysis pipeline as described in Gusareva *et al.* 2019 (18) to allow for comparison with the Singapore samples (Figure S33).

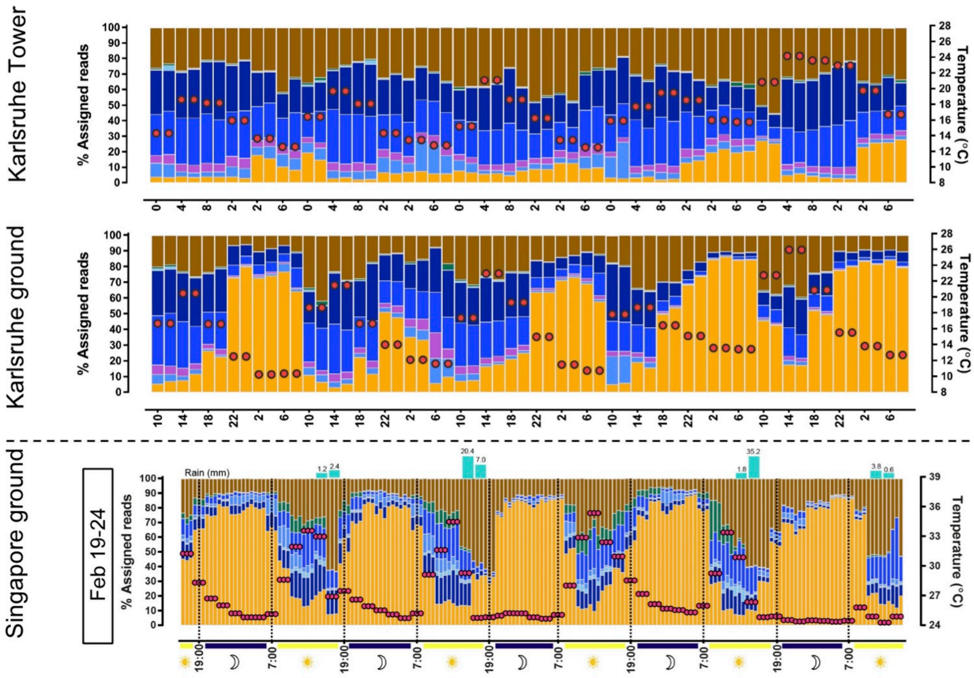

Figure S33. Diel cycle of airborne microbial communities in the temperate climate zone (Karlsruhe, Germany) and in the tropics (Singapore). Samples in Germany were collected for 2 hours every 4 hours for 5 consecutive days whereas samples in Singapore were collected for 2 hours every 2 hours for 5 consecutive days. Replicates for each time point were averaged.

8) <u>Air sampling at higher altitudes with the help of a research aircraft (October 2018)</u>

Air sample collection at altitudes of 300m to 3,500m was conducted in Brunswick, Germany, with the help of a research aircraft. The research aircraft, a Dornier 128-6 high-wing aircraft with two propeller-turbine engines, is owned and operated by the Institute of Flight Guidance at the Technische Universität Braunschweig. The aircraft is equipped with scientific instrumentation for meteorological measurements in the lower atmosphere. In addition, for this study, two interior racks inside the aircraft were modified to accommodate twelve SASS3100 air samplers (Research International). Furthermore, a custom-made air inlet pipe was installed to allow outside air to be funneled into the sampling chambers inside the aircraft cabin where the racks with the SASS air samplers were located. This set-up allowed for outside air to be samples within the sampling chambers inside the aircraft (Fig. S34, Table S3).

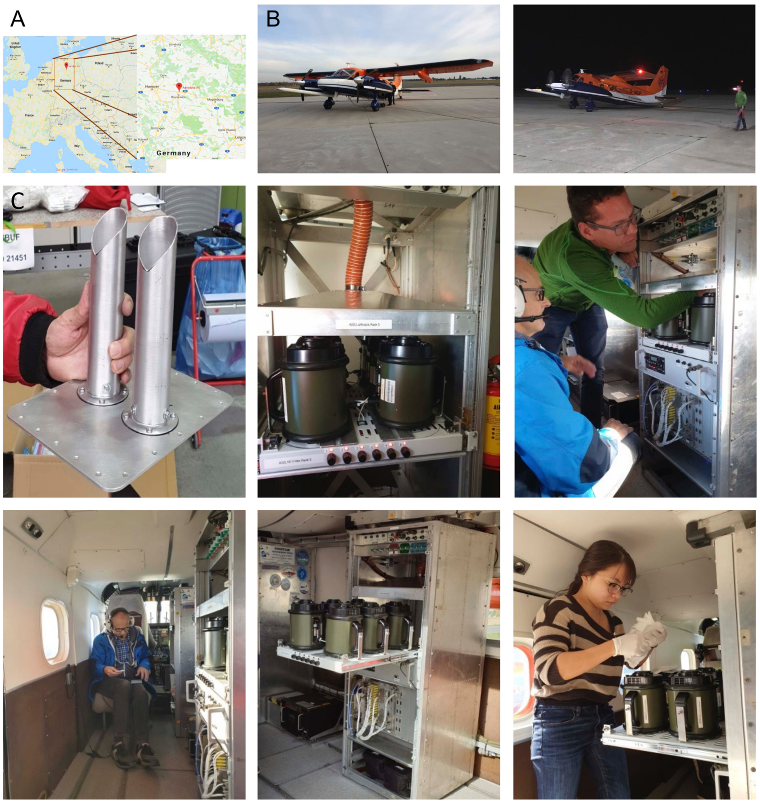

Figure S34: High-altitude air sampling in the vertical air column. A) Air sampling was performed in Brunswick, Germany, on 9, 10 and 12 of October 2018. B) The experiments were carried out with a research aircraft (Dornier 128-6), operated by two pilots, one meteorologist and one air sampling expert. A total of three day flights and two night flights were undertaken. C) Air sampling was performed in two open chambers inside the aircraft cabin, consisting of custom-made tubing and modified hoods, flooded by the dynamic pressure of the outside air that allowed for sampling of external air within the chambers. Each chamber was large enough to accommodate six SASS air samplers for a total of twelve air samplers that could be operated simultaneously on the aircraft. The air samplers were secured on retractable trays which facilitated filter retrieval/change as well as cleaning of the air samplers in between sampling cycles.

8.1) Construction of aerodynamically-shaped custom-made air inlet pipes

A custom-made air inlet was installed on top of the cabin, consisting of two metal pipes with 45-degree bevels. The bevels were placed perpendicular to the outside airflow. Each inlet pipe had a diameter of 2 inches and an approximate cross-sectional area of $20cm^2$. Inside the cabin, the pipes connected to plastic tubing that each led to one of two pull-out rack inserts, each holding six samplers.

Table S3. Approximate calculations of the air exchange rate at different altitudes.

Altitude	Airspeed	Average Volumetric Flow Rate	Average Air Flow Rate	Air Flow Rate Percent Deviation
3,500m	67m/s	$8.04m^3/min$	0.11055kg/s	77,0%
2,100m	67m/s	$8.04m^3/min$	0.12998kg/s	90,5%
1,200m	63m/s	$7.56m^3/min$	0.13482kg/s	93,9%
400m	63m/s	$7.56m^3/min$	0.14364kg/s	100%

For each flight, live in-flight analysis of meteorological data (e.g. temperature and absolute humidity) collected during ascent to 3,500m determined which two additional sampling heights were to be sampled, one below the inversion layer and one above (altitude 1: 3,500m, altitude 2: 2,000-2,300m, altitude 3: 1,300-1,500m and altitude 4: 300-330m; Fig. S35).

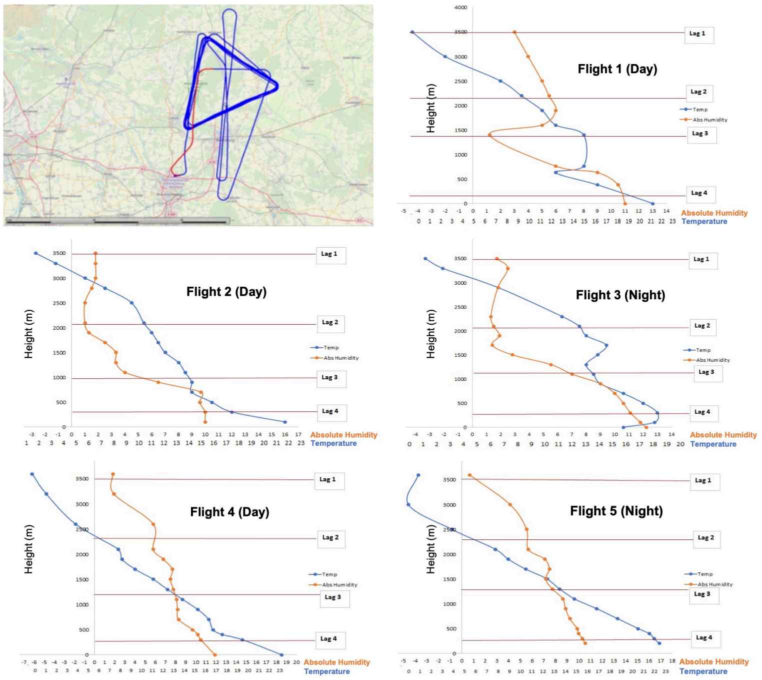

Figure S35: Flight details and live analysis of meteorological parameters. A) Flight path. Each flight lasted for approx. 4 hours and the aircraft was following a repeated triangular pattern of approx. 40km x 40km for all 5 flights to control sampling consistency as much as possible between the different altitudes and also between different flights. B) Air microbiome composition was investigated as a function of meteorological factors such as humidity, temperature, vertical mixing and light intensity as well sampling height. Live in-flight analysis of meteorological data was used to determine optimal sampling altitudes.

Twelve SASS air samplers were operated simultaneously at each height (12 air filters collected per height). Matching ground samples were simultaneously collected at the Brunswick Wolfsburg Airport (BWE). The airport is located approx. 200km SE of the North Sea, 200km SW of the Baltic Sea and 400km NNE of the meteorological tower in Karlsruhe. For the ground samples, 6 SASS air samplers were placed on a trolley (~1m height) outside the hangar for the research aircraft at the airport. The air samplers at the ground were operated simultaneously to the air sampling that was performed on the aircraft at the different heights. At each height, air sampling was conducted for a total duration of 35-50 minutes. In between the sampling cycles at different heights, the air samplers were decontaminated with lab cleaner and 70% ethanol as described above, before installing a new filter. In total, five flights were conducted, three during the day and two at night time:

Flight 1: 9 October 2018: 13:55-17:55 (day flight)
Flight 2: 10 October 2018: 13:10-17:10 (day flight)
Flight 3: 10 October 2018: 20:45-00:45 (night flight)
Flight 4: 12 October 2018: 12:55-16:55 (day flight)
Flight 5: 12 October 2018: 20:45-00:45 (night flight)

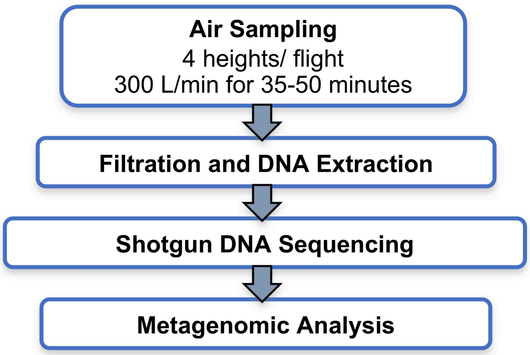

Figure S36: Sample collection and processing pipeline for samples collected in the research aircraft experiment. Air samples were collected at four different altitudes for each flight. The air filters were then subjected to biomass removal, DNA extraction, shotgun DNA sequencing, followed by metagenomic analysis.

For each flight, 12 SASS air samples were simultaneously collected at each of the four altitudes mentioned above. In addition, 6 matching ground samples were collected for each altitude, resulting in 72 air samples being collected per flight or 360 filters in total across the 5 flights (Figure S36). The biomass from multiple filters from the same sampling height was then pooled to obtain sufficient DNA for metagenomic sequencing (see details in the methods section above).

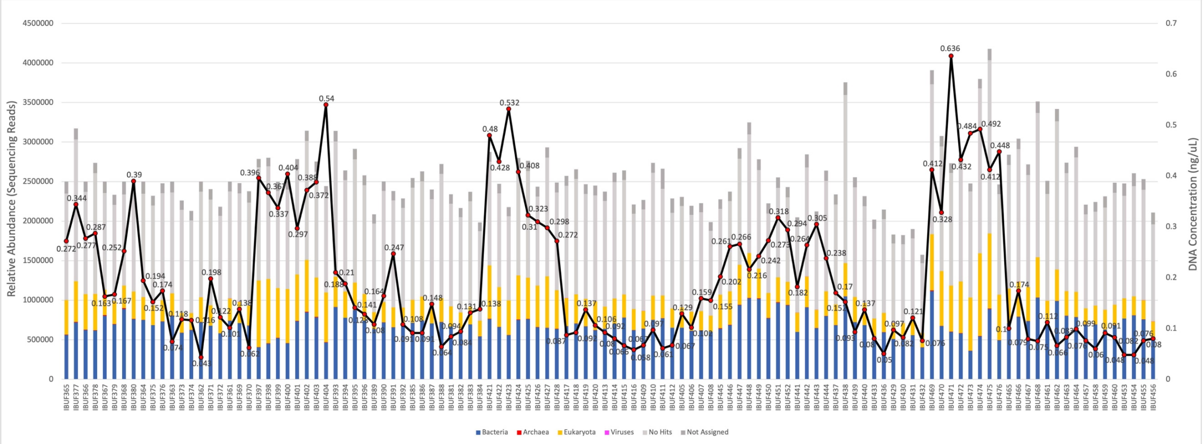

Figure S37. Domain-level comparison between all samples from research aircraft (RA) experiment (flights and ground samples). Relative abundances are shown based on absolute number of sequencing reads obtained for each sample. Also shown are the obtained DNA concentrations for each individual sample. Samples are grouped by flight with corresponding ground samples shown first, followed by the flight samples.

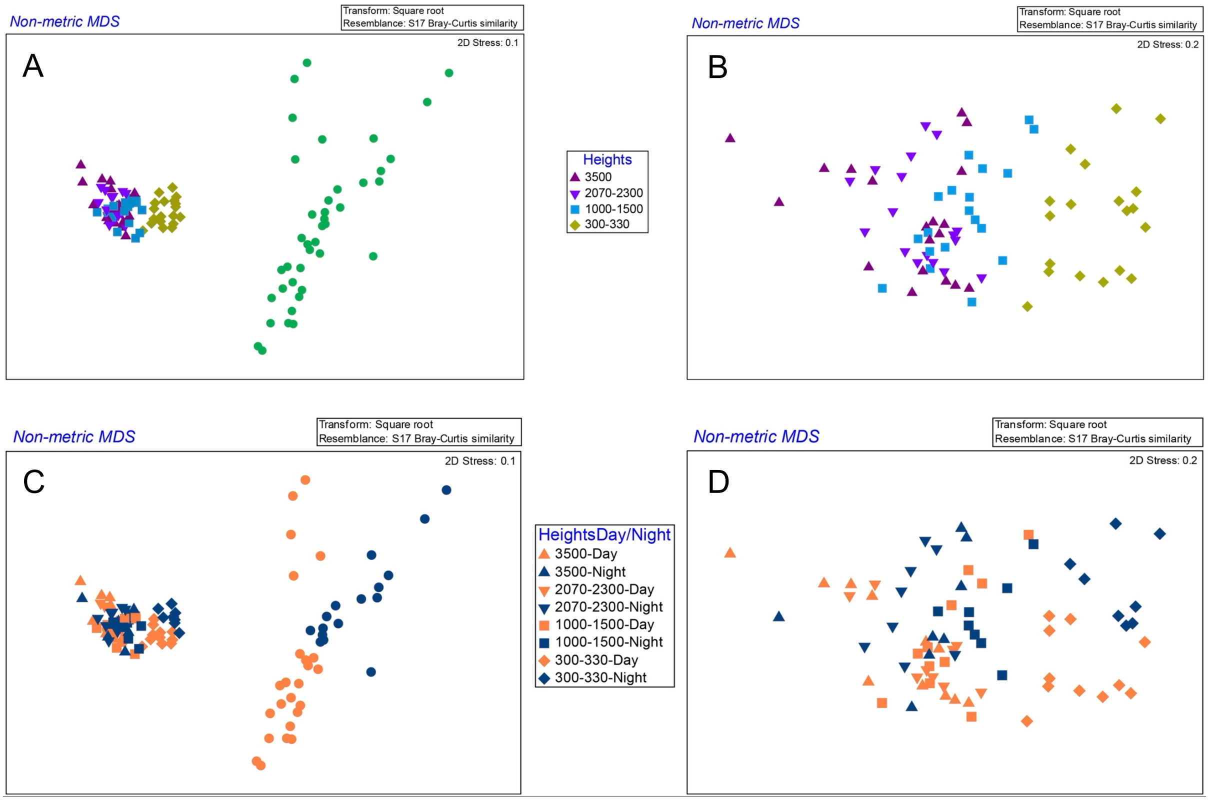

Figure S38. Non-metric MDS plots. A) All flight and corresponding ground samples. B) Flight samples only. C) All flight and corresponding ground samples, colored by sample collection time (day or night). D) Flight samples only, colored by sample collection time (day or night).

8.2) Vertical transport estimations within the atmospheric boundary layer

The atmospheric boundary layer (ABL) is the lowermost layer above the ground which is directly influenced by its contact with the earth's surface. When investigating vertical transport in the atmosphere within the ABL, the state of the ABL is important, e.g., whether it is stable, neutral, or unstable stratified. When the ABL is unstable stratified, vertical motion and transport of gaseous or solid components (e.g., water, ozone, dust, microorganisms) is possible. In case of a stable stratified ABL, these substances remain suspended at the same height as when stability was developed, with the exception of larger particles which may still fall back to the ground very slowly due to gravity. The layer which enables vertical motion is called the mixing layer and its uppermost boundary is the mixing layer height (MLH). After sunrise, the ABL rises and the MLH increases. After noon, the MLH reaches its peak value and vertical transport is most effective within the neutral or slightly unstable layer. Around sunset, a new stable layer, which stops vertical mixing, develops near the ground and the mixing layer of the daytime hours (above the stable layer) is now called the residual layer (RL). Its stability is neutral or slightly stable during the night and vertical motion is suppressed.

To determine the height of the ABL and the state of the atmosphere (stable or unstable stratified), two approaches can be used which are either based on thermal or dynamic parameters. In many instances, both ABL calculation methods result in similar heights:

Thermal-driven ABL calculation approach:

The ABL can be determined using thermal measures, e.g., potential temperature. Here, temperature and pressure measurements are used to calculate vertical profiles of the potential temperature. Increasing potential temperature with height indicates a stable stratified ABL with no vertical motion. Decreasing potential temperature with height, on the other hand, is a sign for an unstable stratified ABL with the possibility of vertical motion.

Dynamics-driven ABL calculation approach:

Alternatively, direct measurements of vertical wind speed can be used to determine the dynamics of the ABL. In this case, the height of the vertical wind speed within the ABL is a direct measure for vertical transport. The effect of vertical transport can be seen when looking at the vertical profiles of trace substances such as water vapor within the mixed layer. For example, mixing is high (effective) when the humidity profile is constant with height, whereas mixing is less effective when humidity decreases with height.

Estimation of the vertical mixing processes within the ABL during the five flights based on thermal- and dynamics-driven ABL calculations (Figure S39; Table S4):

Flight 1, 09 Oct 2018 (daytime)

The MLH estimation using potential temperature and vertical wind speed data results in the same height. At noon on 9 October 2018, the MLH was about 600m a.g.l. Within this layer, the stability was neutral, allowing for vertical transport of microorganisms between the ground and the MLH. Above the MHL, up to 3,500m a.g.l., the stratification was stable, and no vertical mass transport is expected to have occurred.

Flight 2, 10 Oct 2018 (daytime)
For Flight 2, the MLH estimation using potential temperature resulted in a height of 800m whereas the dynamics-driven calculations based on vertical wind speed data indicated that the MLH was at 950m. At noon on 10 October 2018, the uppermost 150m of the ABL showed a wavy structure which may have been caused by high horizontal wind speed of up to 10m/s within that layer. Between the ground and the MHL at 800m/950m, the stability was neutral, enabling vertical transport of biomass between the ground and the MLH. Above the MLH, up to 3,500m a.g.l., the stratification was stable, likely preventing vertical mass transport.

Flight 3, 10 Oct 2018 (nighttime)
During the 7-hour interval between Flight 2 at noon and Flight 3 at night on the same day, a strongly stable layer developed between the ground and 500m a.g.l. Above that layer, up to 1,050m, the stratification based on potential temperature measurements was still stable, but less than in the layer below. Vertical wind speed measurements indicate that vertical motion occurred between the ground and 800m a.g.l. Although stable stratified, this indicates that some vertical transport within the stable layer that is driven by turbulence may have still occurred. In the layer around 500m, the horizontal wind speed was rather high (up to 15m/s). This increases turbulence and results in vertical transport. Above that layer, up to 3,500m a.g.l., the stratification was stable, likely preventing vertical mass transport.

Flight 4, 12 Oct 2018 (daytime)
On this day, MLH estimation based on potential temperature results in a height of 600m whereas vertical wind speed-based calculations predict a height of 700m. At noon on 12 October 2018, the uppermost 150m of the ABL again shows a wavy pattern caused by turbulence and rising air bubbles. Within the layer between the ground and 600m/700m, the stability was neutral, allowing for vertical transport of microorganisms between the ground and the MLH. Above the MLH, up to 3,500m a.g.l., the stratification was stable, and no significant vertical mass transport may have occurred.

Flight 5, 12 Oct 2018 (nighttime)
During the 7-hour interval between Flight 4 at noon and Flight 5 at night on the same day, a very stable layer developed between the ground and 100m a.g.l. (7K/100m). Above that layer, up to 500m, the stratification based on potential temperature was still stable but less pronounced than in the very stable layer below (0.5K/100m). The upper boundary of the residual layer (RL) lied at 1,400m a.g.l., which is the same height as the upper boundary of the mixed layer in the afternoon of the same day (Flight 4). Vertical wind speed measurements also indicate vertical motion between the ground and the RL height at 1,400m a.g.l. Although slightly stable stratification was present, this indicates that some vertical transport within the stable layer occurred, driven by turbulence due to vertical and horizontal motion within this layer. In the layer around 1,400m, the horizontal wind speed was up to 8 m/s. This promotes turbulence and results in vertical transport. Above this layer, up to 3,500m a.g.l., the stratification was stable, likely preventing vertical mass transport.

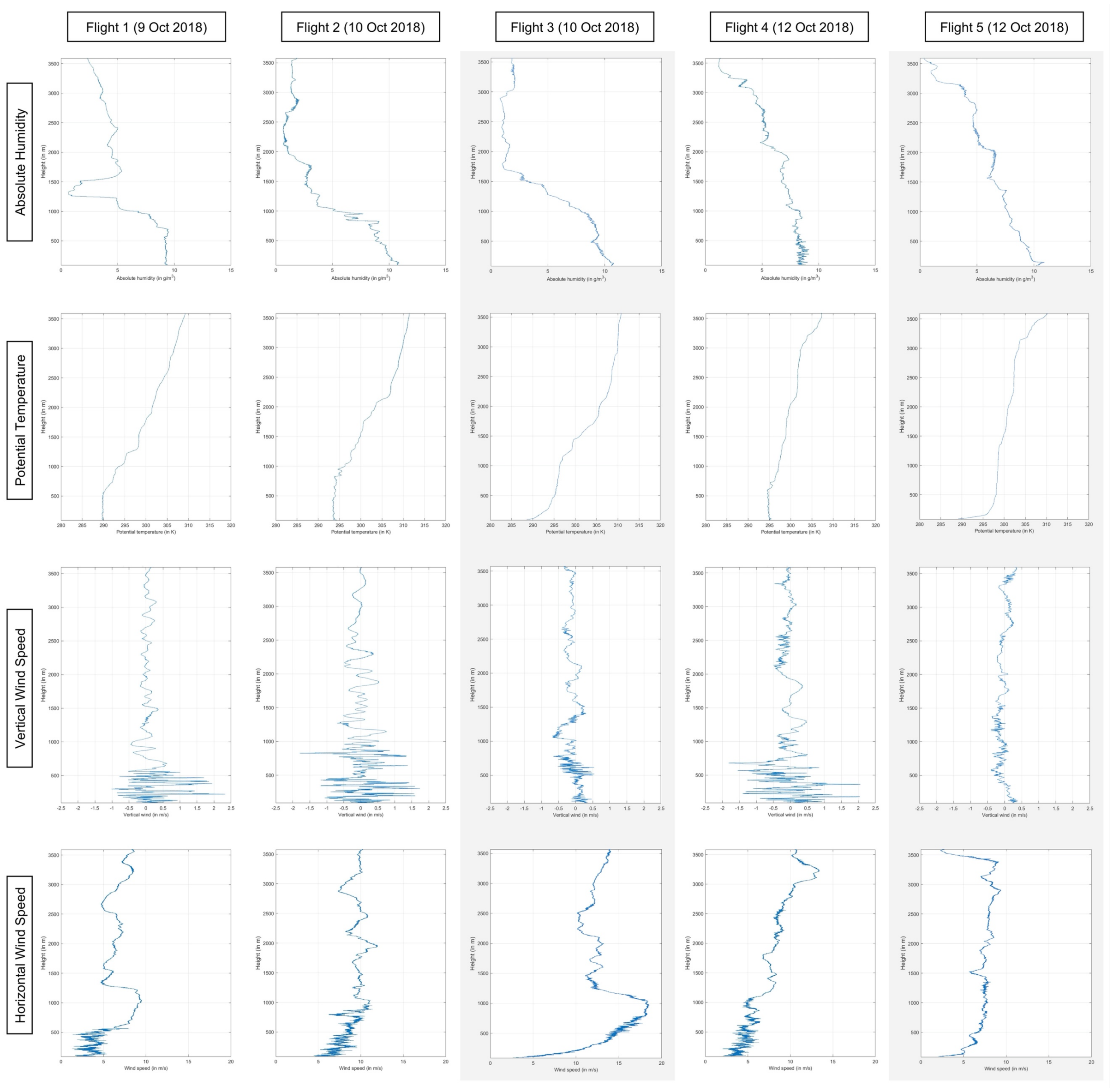

Figure S39. Live in-flight analysis of meteorological parameters. Vertical profiles for potential temperature (T_{pot}), absolute humidity, vertical wind speed (w), and horizontal wind speed (v) are shown between the ground and 3,500m height for each flight. Meteorological parameters were recorded at the beginning of each flight during the initial ascent to 3,500m. Flights conducted during night hours are shaded in grey.

Table S4: Mixing layer height (MLH) estimation using thermal and dynamical atmospheric boundary layer (ABL) definition for flights conducted in October 2018 in Brunswick, Germany.

Flight #	Date	Flight Time (local)	Max. Ascent	Ascent Time (local)	MLH (based on PT)	MLH (based on AH)	MLH (based on vertical wind speed)	MLH (based on horizontal wind speed)	Stability	Wind Speed within ABL
1	9 Oct 2018	13:54 - 17:49	3500m GND	14:05 - 14:17	580m	700m	600m	600m	neutral (up to 600m)	2-5m/s
2	10 Oct 2018	13:00 - 17:03	3500m GND	13:03 - 13:14	800m (max. 950m)	800m	950m	950m	neutral (up to 800m), partly up to 1000m	6-10m/s
3	10 Oct 2018	20:37 - 00:38	3500m GND	20:40 - 20:53	approx. 1050m (residual layer)	approx. 1000m	800m	800m up to 1000m	stable (up to 500m); very stable (up to 100m)	3-15m/s; 2-5m/s
4	12 Oct 2018	13:00 - 16:49	3500m GND	12:54 - 13:05	600m (max. 750m)	600 (max. 800m)	700m	600m	stable (up to 600m)	3-6m/s
5	12 Oct 2018	20:24 - 00:44	3500m GND	20:40 - 20:51	500m or 1300m (two residual layers)	100m (stable surface layer)	1400m (residual layer)	1400m (residual layer)	very stable (up to 100 m); stable (up to 500m); slightly stable (up to 1300m, residual layer)	2-5m/s; 5-7m/s; 7-8m/s

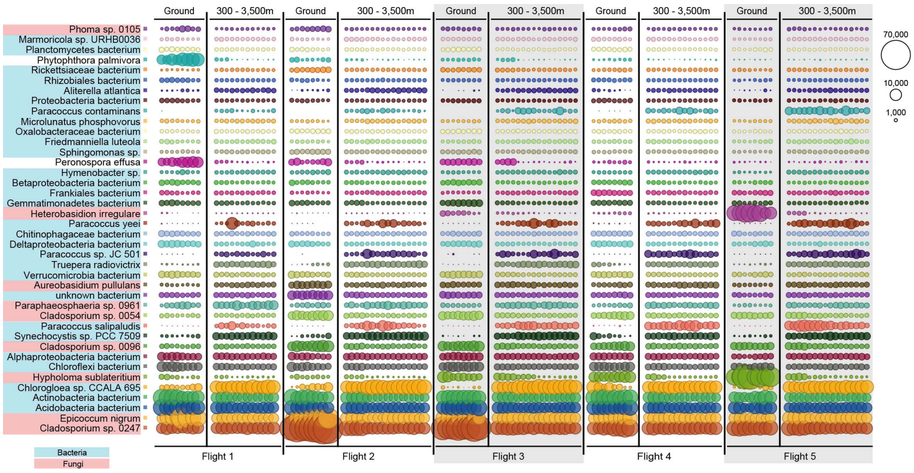

Figure S40. Top 40 all species. Ground samples followed by flight samples, grouped together by flight. Flight samples are ordered by altitude with the lowest altitude (300m) displayed first, followed by the higher altitudes. Ground samples are ordered according to their corresponding flight samples, e.g., ground samples that were taken simultaneously to the 300m flight samples are show first. Flights that were undertaken during night hours are shaded in grey.

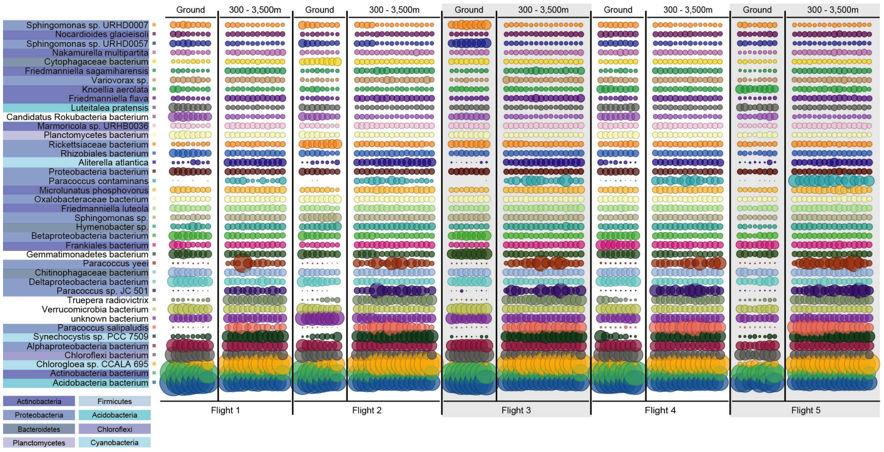

Figure S41. Top 40 bacteria. Ground samples followed by flight samples, grouped together by flight. Flight samples are ordered by altitude with the lowest altitude (300m) displayed first, followed by the higher altitudes. Ground samples are ordered according to their corresponding flight samples, e.g., ground samples that were taken simultaneously to the 300m flight samples are show first. Flights that were undertaken during night hours are shaded in grey.

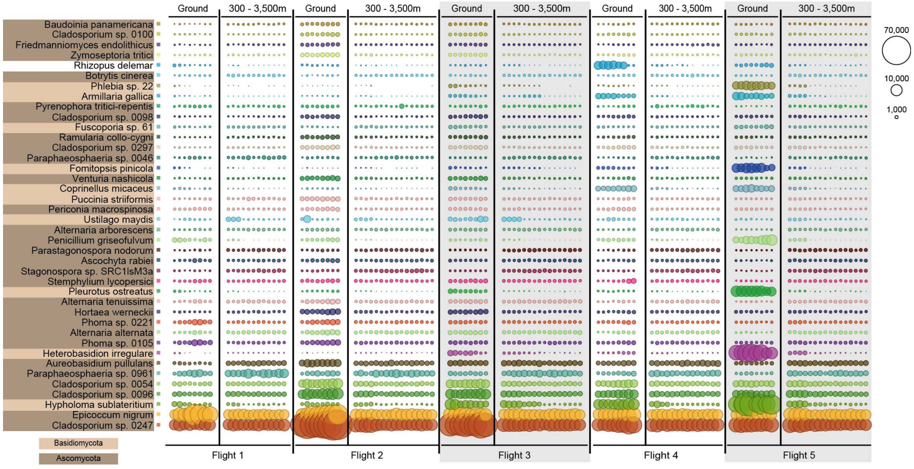

Figure S42. Top 40 fungi. Ground samples followed by flight samples, grouped together by flight. Flight samples are ordered by altitude with the lowest altitude (300m) displayed first, followed by the higher altitudes. Ground samples are ordered according to their corresponding flight samples, e.g., ground samples that were taken simultaneously to the 300m flight samples are show first. Flights that were undertaken during night hours are shaded in grey.

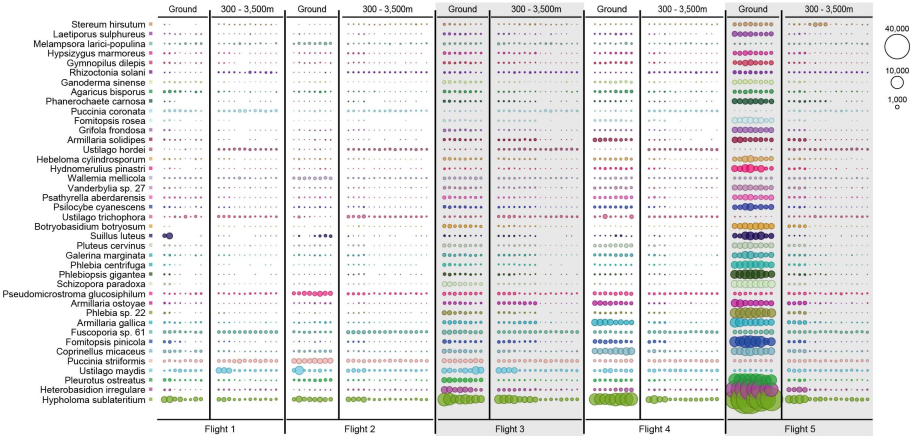

Figure S43. Top 40 basidiomycota. Ground samples followed by flight samples, grouped together by flight. Flight samples are ordered by altitude with the lowest altitude (300m) displayed first, followed by the higher altitudes. Ground samples are ordered according to their corresponding flight samples, e.g., ground samples that were taken simultaneously to the 300m flight samples are show first. Flights that were undertaken during night hours are shaded in grey.

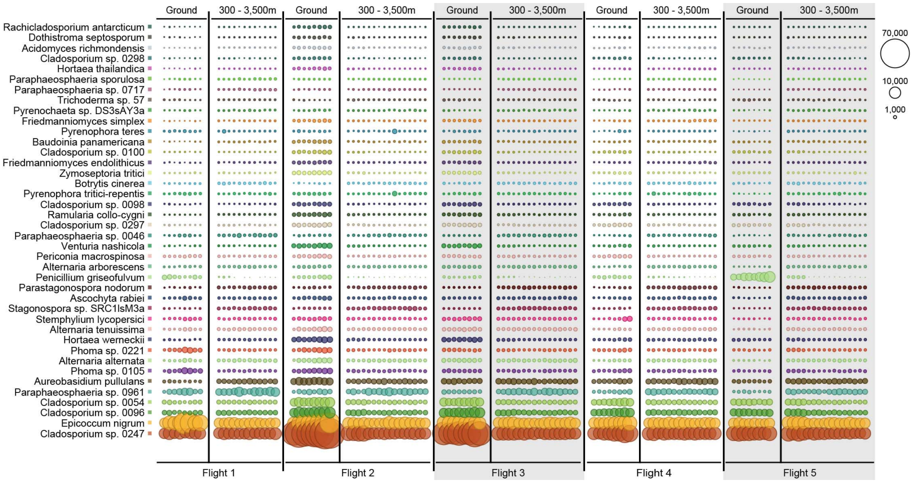

Figure S44. Top 40 ascomycota. Ground samples followed by flight samples, grouped together by flight. Flight samples are ordered by altitude with the lowest altitude (300m) displayed first, followed by the higher altitudes. Ground samples are ordered according to their corresponding flight samples, e.g., ground samples that were taken simultaneously to the 300m flight samples are show first. Flights that were undertaken during night hours are shaded in grey.

8.3) Research aircraft (RA) experiment; ground samples only

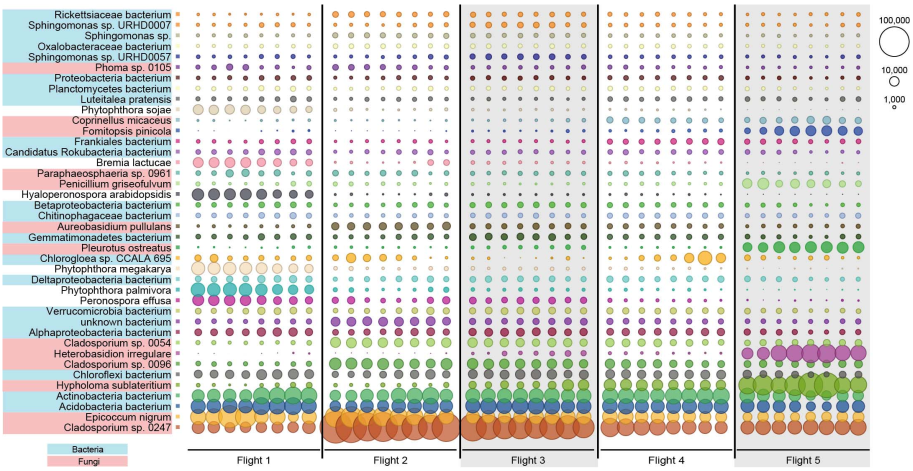

Figure S45. Top 40 all species. Research aircraft (RA) ground samples only, sorted by sampling time and grouped by corresponding flight. Flights conducted during night hours are shaded in grey. Bubble sizes are proportional to the number of sequencing reads assigned to each taxon.

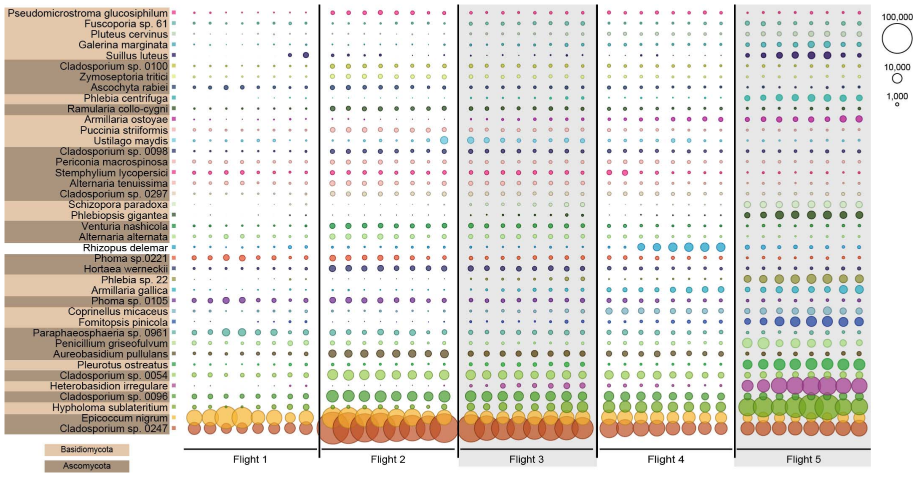

Figure S46. Top 40 fungi. Research aircraft (RA) ground samples only, sorted by sampling time and grouped by corresponding flight. Flights conducted during night hours are shaded in grey. Bubble sizes are proportional to the number of sequencing reads assigned to each taxon.

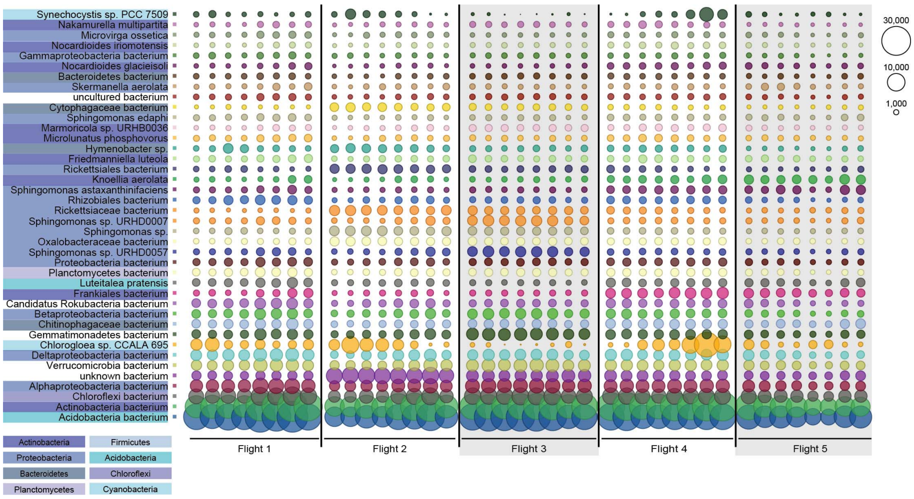

Figure S47. Top 40 bacteria. Research aircraft (RA) ground samples only, sorted by sampling time and grouped by corresponding flight. Flights conducted during night hours are shaded in grey. Bubble sizes are proportional to the number of sequencing reads assigned to each taxon.

8.4) Research aircraft (RA) experiment; flight samples only (samples grouped by flight)

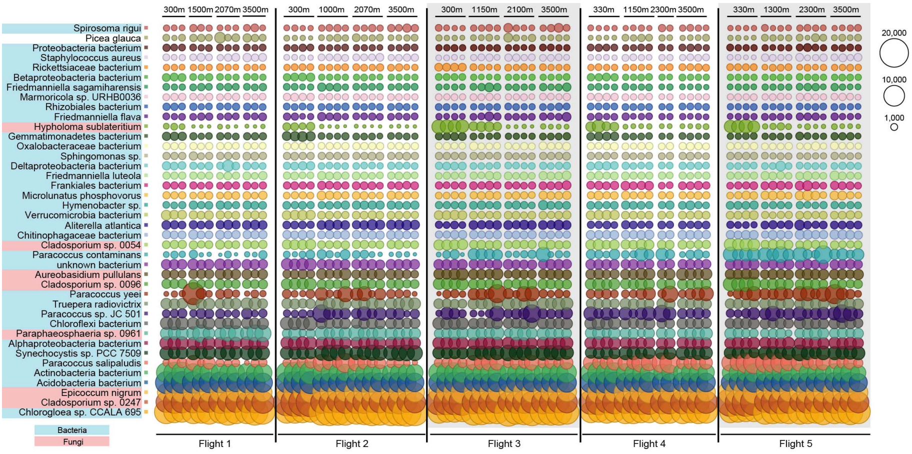

Figure S48. Top 40 all species. Research aircraft (RA) flight samples only, sorted by altitude and grouped by flight. Flights conducted during night hours are shaded in grey. Bubble sizes are proportional to the number of sequencing reads assigned to each taxon.

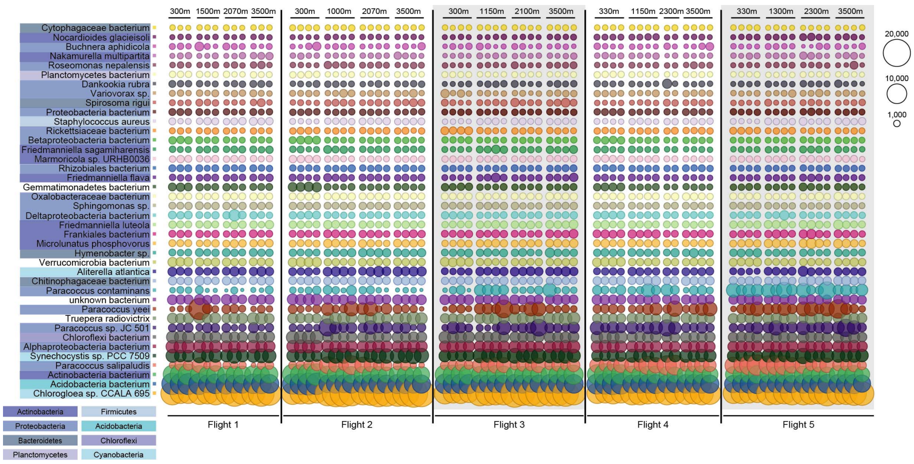

Figure S49. Top 40 bacteria. Research aircraft (RA) flight samples only, sorted by altitude and grouped by flight. Flights conducted during night hours are shaded in grey. Bubble sizes are proportional to the number of sequencing reads assigned to each taxon.

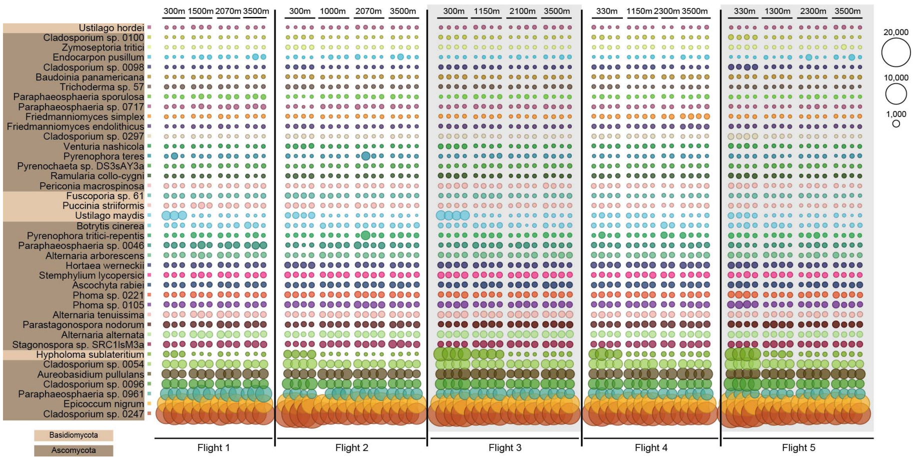

Figure S50. Top 40 fungi. Research aircraft (RA) flight samples only, sorted by altitude and grouped by flight. Flights conducted during night hours are shaded in grey. Bubble sizes are proportional to the number of sequencing reads assigned

8.5) Research aircraft (RA) experiment; flight samples only (samples grouped by height)

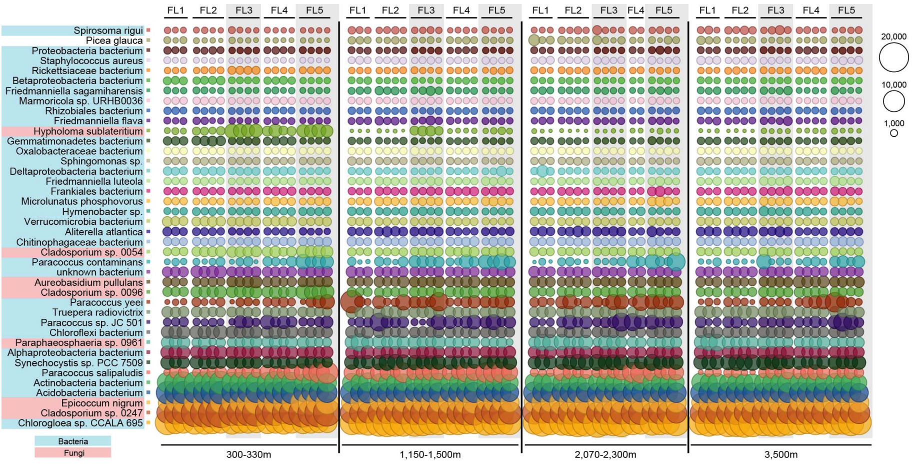

Figure S51. Top 40 all species. Research aircraft (RA) flight samples only, sorted by flight and grouped by altitude. Flights conducted during night hours are shaded in grey. Bubble sizes are proportional to the number of sequencing reads assigned to each taxon.

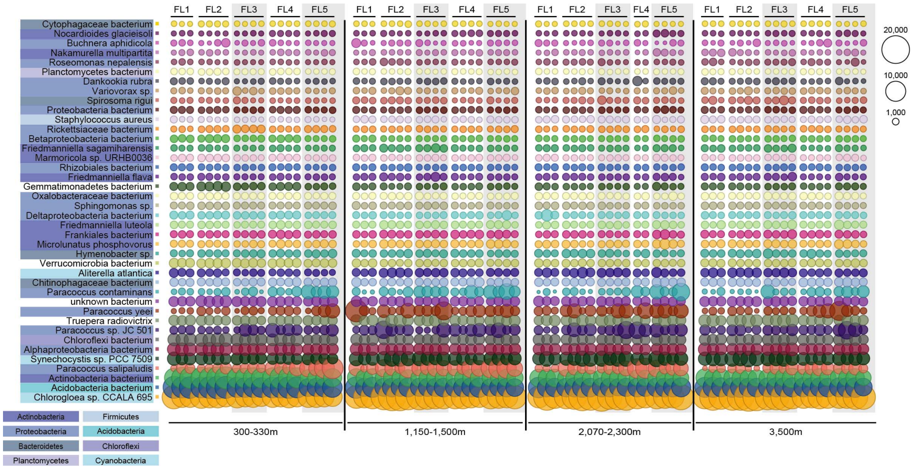

Figure S52. Top 40 bacteria. Research aircraft (RA) flight samples only, sorted by flight and grouped by altitude. Flights conducted during night hours are shaded in grey. Bubble sizes are proportional to the number of sequencing reads assigned to each taxon.

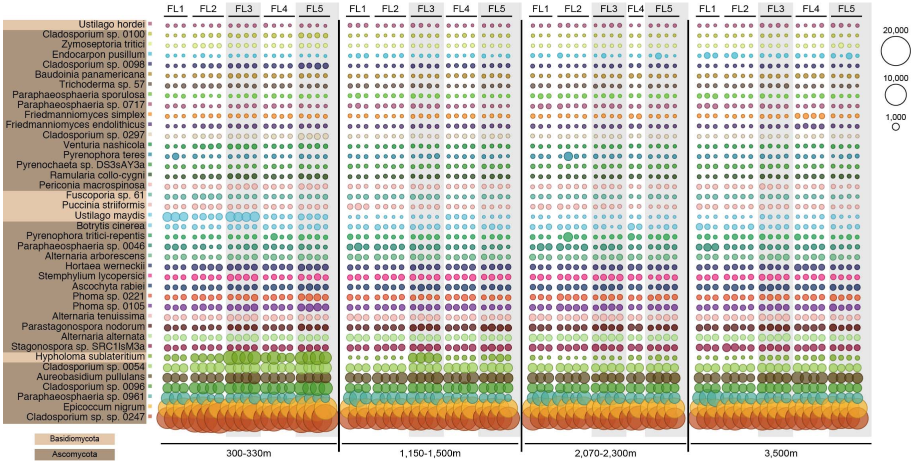

Figure S53. Top 40 fungi. Research aircraft (RA) flight samples only, sorted by flight and grouped by altitude. Flights conducted during night hours are shaded in grey. Bubble sizes are proportional to the number of sequencing reads assigned to each taxon.

8.6) Comparison between 300m samples from the research aircraft (RA) experiment and the 200m samples from the meteorological tower (MT) experiment

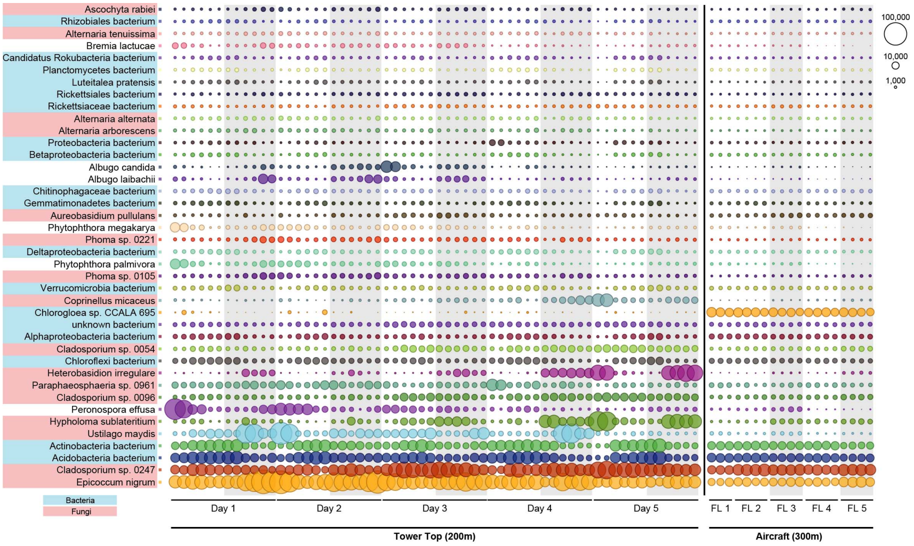

Figure S54. Top 40 all species. Tower 200m samples grouped together by sampling day, followed by 300m aircraft samples grouped by flight. Samples collected during night hours are shaded in grey. Bubble sizes are proportional to the number of sequencing reads assigned to each taxon.

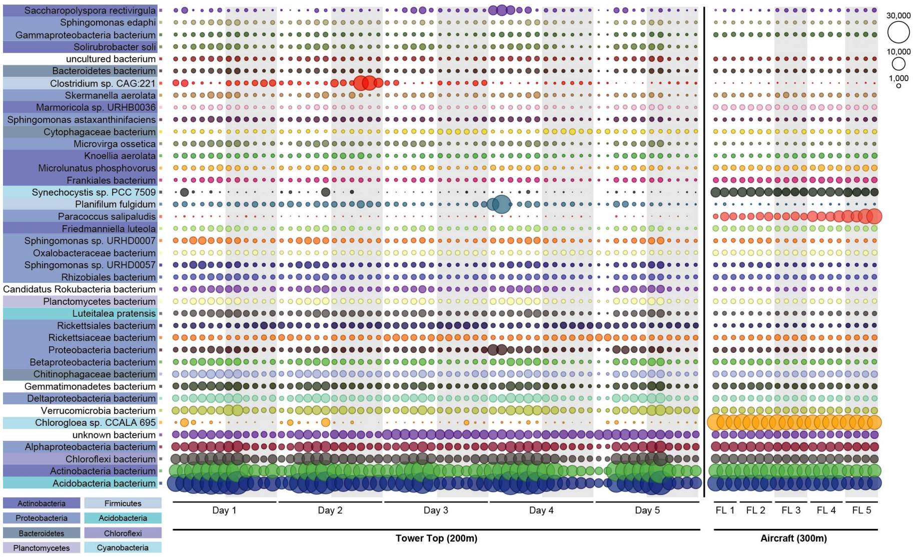

Figure S55. Top 40 bacteria. Tower 200m samples grouped together by sampling day, followed by 300m aircraft samples grouped by flight. Samples collected during night hours are shaded in grey. Bubble sizes are proportional to the number of sequencing reads assigned to each taxon.

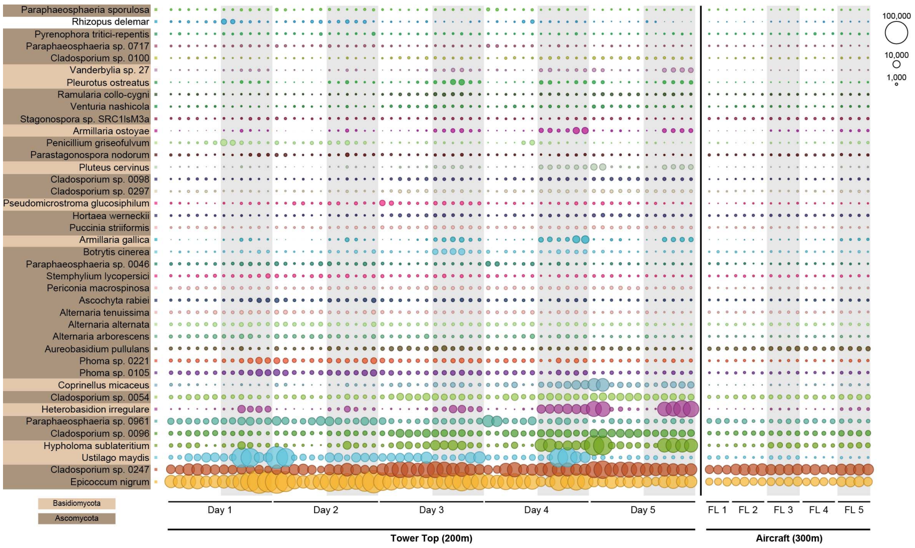

Figure S56. Top 40 fungi. Tower 200m samples grouped together by sampling day, followed by 300m aircraft samples grouped by flight. Samples collected during night hours are shaded in grey. Bubble sizes are proportional to the number of sequencing reads assigned to each taxon.

8.7) Comparison of ground samples collected at the airport in Brunswick as part of the research aircraft (RA) experiment and at the base of the tower in Karlsruhe as part of the meteorological tower (MT) experiment

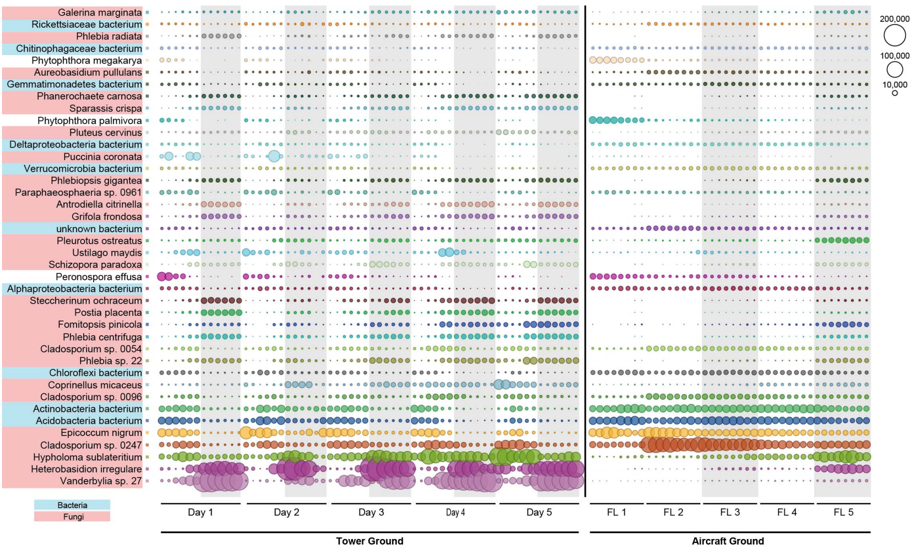

Figure S57. Top 40 all species. Karlsruhe tower ground samples, followed by Brunswick aircraft ground samples. Karlsruhe samples are sorted by sampling day and Brunswick samples by corresponding flight. Samples collected during night hours are shaded in grey. Bubble sizes are proportional to the number of sequencing reads assigned to each taxon.

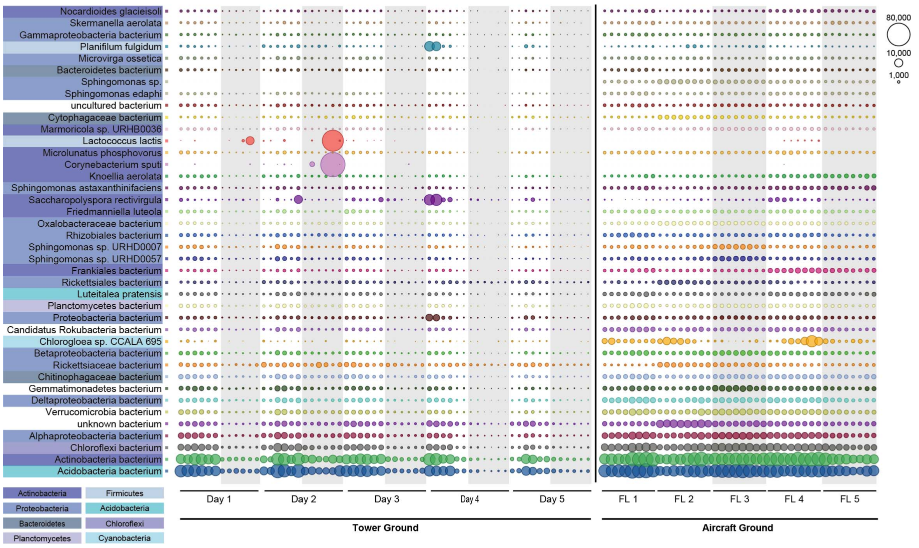

Figure S58. Top 40 bacteria. Karlsruhe tower ground samples, followed by Brunswick aircraft ground samples. Karlsruhe samples are sorted by sampling day and Brunswick samples by corresponding flight. Samples collected during night hours are shaded in grey. Bubble sizes are proportional to the number of sequencing reads assigned to each taxon.

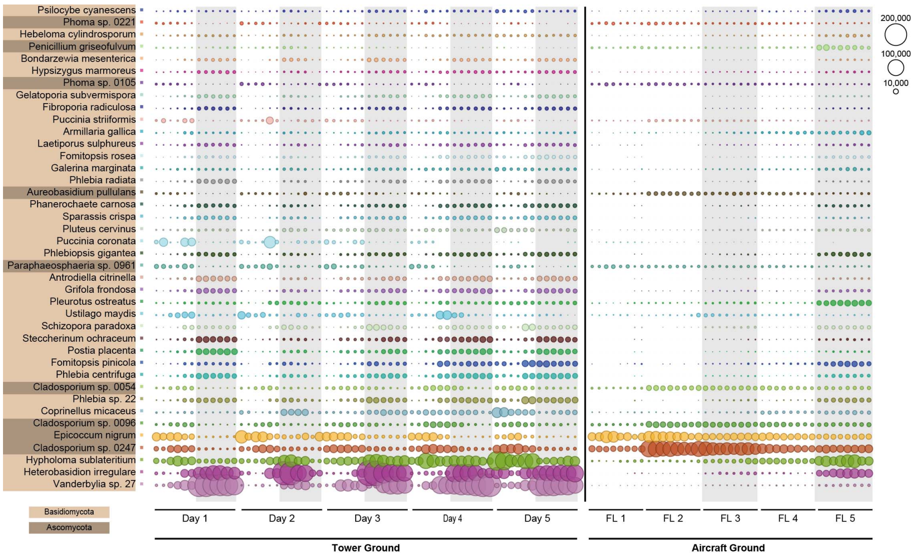

Figure S59. Top 40 fungi. Karlsruhe tower ground samples, followed by Brunswick aircraft ground samples. Karlsruhe samples are sorted by sampling day and Brunswick samples by corresponding flight. Samples collected during night hours are shaded in grey. Bubble sizes are proportional to the number of sequencing reads assigned to each taxon.

8.8) Comparison of ground samples, sorted by time of collection

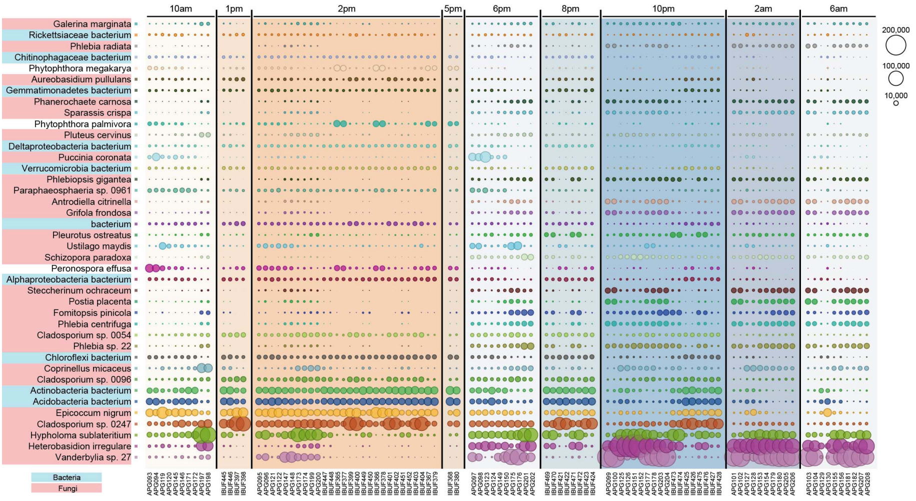

Figure S60. Top 40 all species. Karlsruhe tower ground samples and Brunswick aircraft ground samples, sorted by time of sample collection. Bubble sizes are proportional to the number of sequencing reads assigned to each taxon.

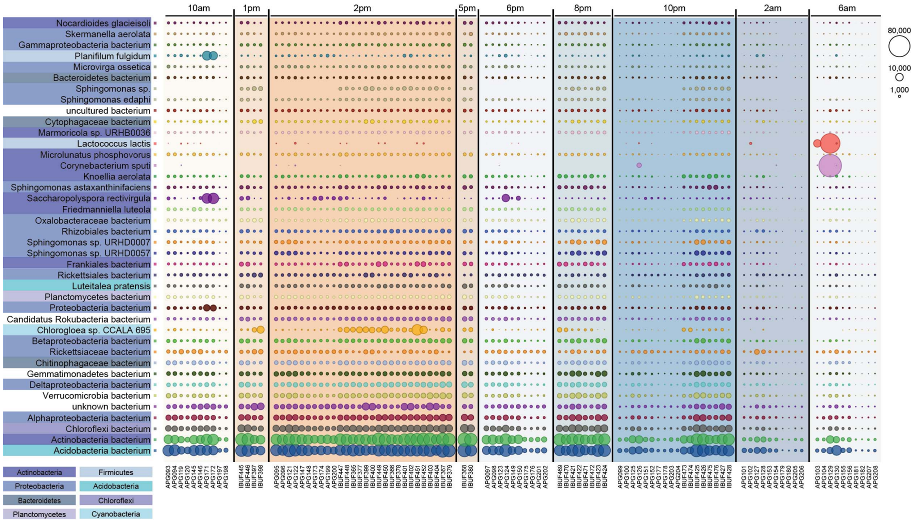

Figure S61. Top 40 bacteria. Karlsruhe tower ground samples and Brunswick aircraft ground samples, sorted by time of sample collection. Bubble sizes are proportional to the number of sequencing reads assigned to each taxon.

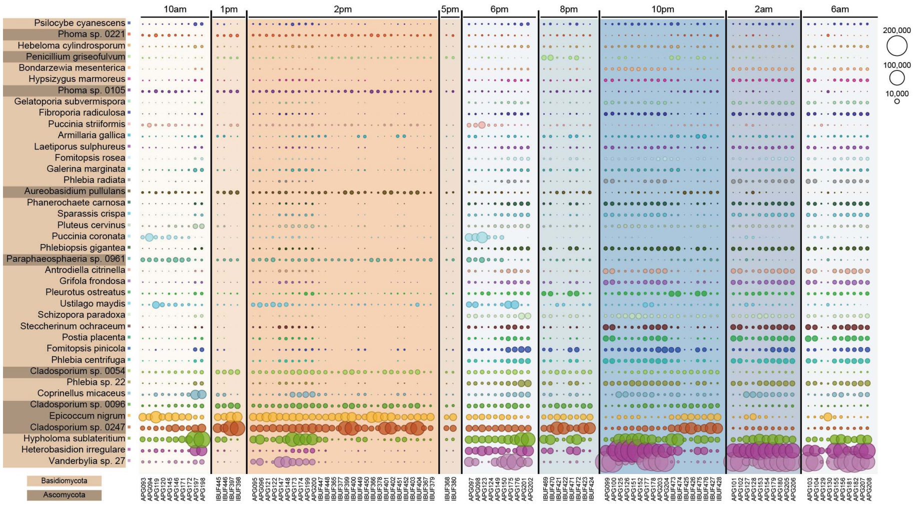

Figure 62. Top 40 fungi. Karlsruhe tower ground samples and Brunswick aircraft ground samples, sorted by time of sample collection. Bubble sizes are proportional to the number of sequencing reads assigned to each taxon.

9) Rarefaction curves of different sequencing depths

A representative set of samples from the MT and RA experiments were independently analyzed with different sequencing depths to assess the saturation of the number of identified species. For the MT experiment, ground-level air samples showed a lower diversity than tower-top air samples. This difference was the most prominent during night-time when minimal vertical mixing occurs, compounded by active release of Basidiomycota taxa which dominated the ground-level air. During daytime, the high degree of vertical mixing allowed height-associated taxa to enrich the ground-level air, which resulted in minimized differences in diversity between the two air layers (Fig. S63a).

The ground air samples of the RA experiment were found to be significantly more diverse. This is likely due to the difference in land-type between the two sampling campaigns (MT = forested, RA = grassland). Despite such differences, the higher altitude (3,500m) air samples were still found to have higher diversity than the ground air samples, albeit at a lesser extent (Fig. S63b). Most importantly, the ultra-deep sequencing of the two samples with the highest diversity indicated that saturation in species identification is not yet reached, even at the scale of 30 million reads per sample (Fig. S63c).

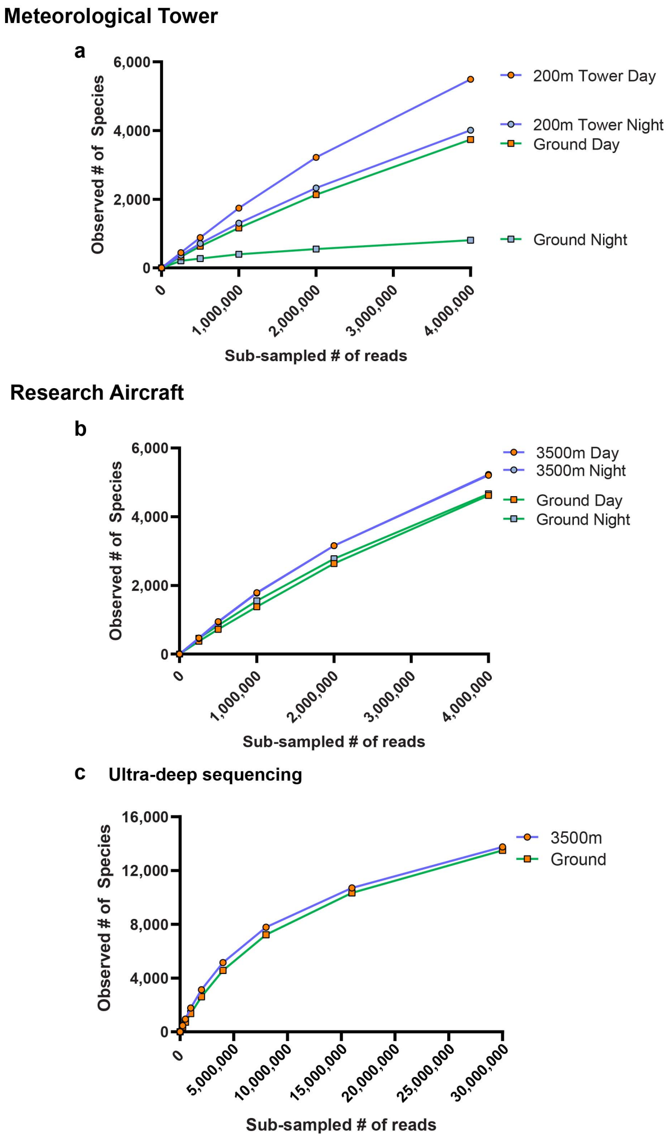

Figure S63. Rarefaction curves of representative air samples chosen from the MT and RA experiments.

10) <u>Presence/absence analysis of bacterial and fungal taxa in the vertical air column</u>

Using a presence/absence analysis, bacterial and fungal taxa were binned based on their temporal (day/night) and vertical distribution (0 and 200m MT, 0 and 1,000 - 3,500m RA) (Fig. S64). While the results are categorized at the taxonomic levels of Domain, Kingdom and Phylum, the underlying taxonomic resolution for each taxon is at species level. Fungal diversity was found to largely overlap between all height levels in both experiments (MT and RA). In detail, during daytime, the fungal taxa overlap between the ground and the height reached 85% (RA) and 88% (MT). During nighttime the overlap between ground and the various heights reduced to 73% (RA) and 84% (MT). Consequently, only a small number of fungal taxa were specific to either ground (6 - 23%) or height (4% @1,000-3500m RA, 10% @200m MT). The small fraction of fungal taxa specific to ground and height indicates the ground as a likely source for both settings. In contrast, overlap in bacterial diversity noticeably differs between ground and heights, in a diel-specific manner. Daytime overlap of bacterial OTUs between ground and height was 77% (RA) and 76% (MT). Night-time stratification of bacteria in the RA and MT experiment was more prominent, resulting in 61% and 49% overlap, respectively. The observed patterns in presence/absence are a consequence of the specific behavior of each of the bacterial phyla Actinobacteria, Proteobacteria, Firmicutes and Bacteroides. The higher fraction of height-specific bacterial taxa during night-time indicates these to originate from sources other than local ground sources.

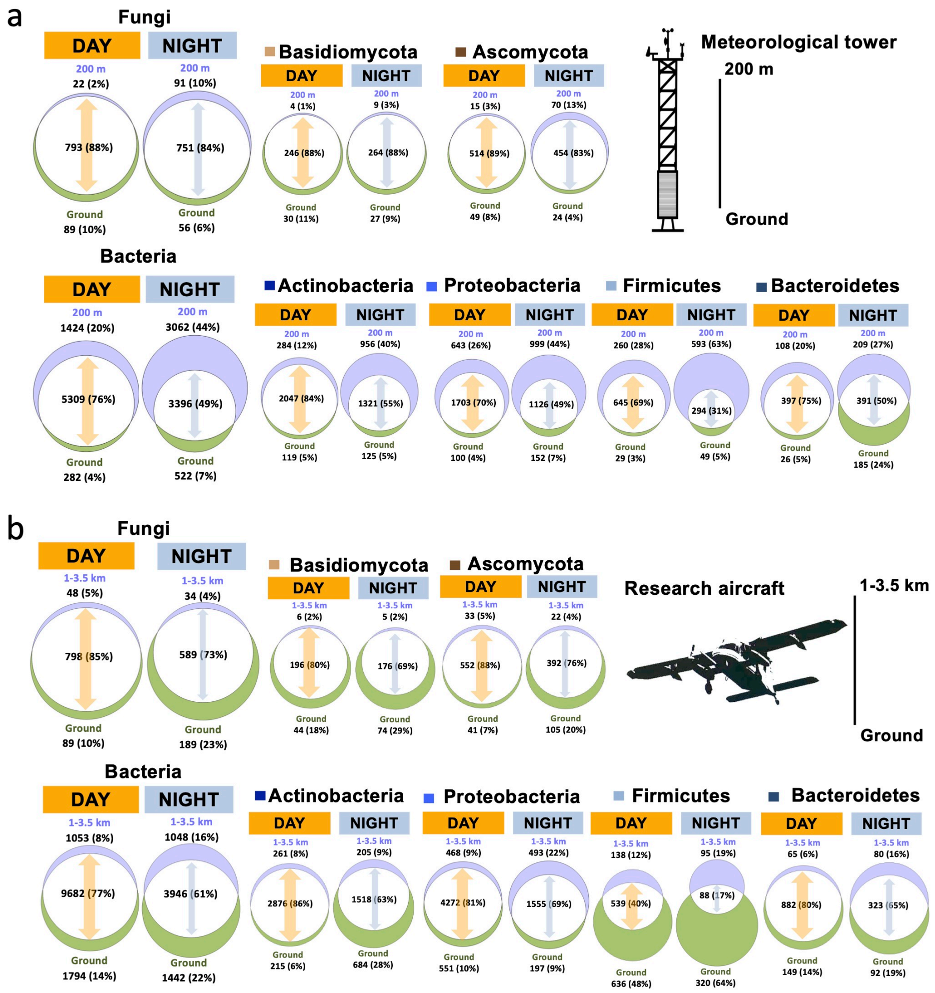

Figure S64. Venn diagram highlighting the number of detected bacterial and fungal species from air samples collected at different heights in the MT and RA experiment. The species were grouped based on their respective phyla.

11) Air Sampling at Mount Säntis, Switzerland

Air samples were collected on a roof top at the summit (2,502m) and near the car park at the base of the mountain on 4 Nov 2017 at 12pm and 3pm, respectively (Fig. S65). Mount Säntis is the highest mountain in the area and a popular tourist site. The buildings on the summit house several restaurants as well as a weather station.

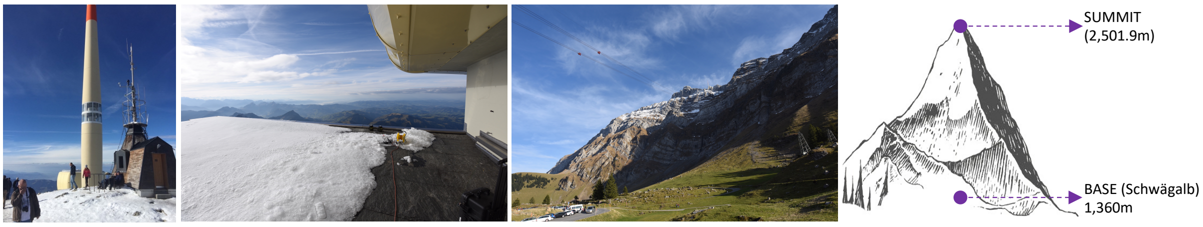

Figure S65. Air sampling at Mount Säntis, Switzerland. Overview of sampling sites.

With the exception of some bacterial species that may originate from a nearby wastewater treatment facility on the summit, no major differences were observed in the microbial composition of air samples collected at the summit and the base of the mountain (Figures S66 – S69). This may be explained by local wind systems and atmospheric turbulence caused by temperature differences between the mountain slopes and adjacent valleys. During daytime when the samples were collected, the air along mountain slopes is heated up more than air at the same elevation over an adjacent valley, resulting in the warm air rising up (upslope winds) and drawing of cooler air up from nearby valley ground layers. This results in mixing of air layers from different altitudes and allows for airborne microbial organisms from lower altitudes to reach air layers at the summit of the mountain. As such, we deemed mountains as unsuitable sites for accurate characterization of bioaerosols associated with different layers in the vertical column of the atmosphere.

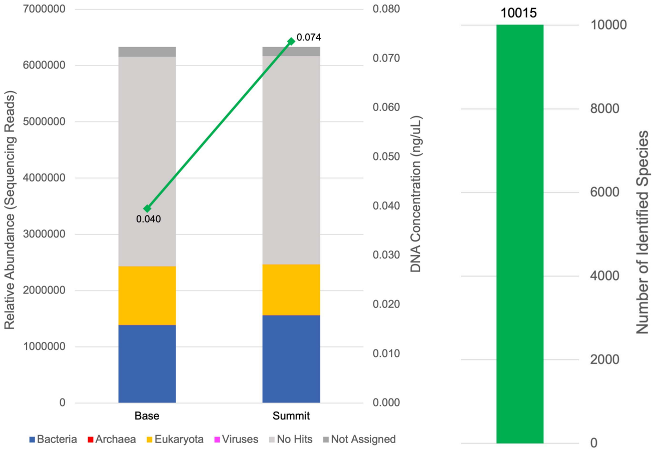

Figure S66. Taxonomic overview of relative abundances at superkingdom-level based on number of assigned sequencing reads for air samples collected at Mount Säntis. DNA concentrations of analyzed samples and total number of identified species (averages per time point and sampling location) are also shown.

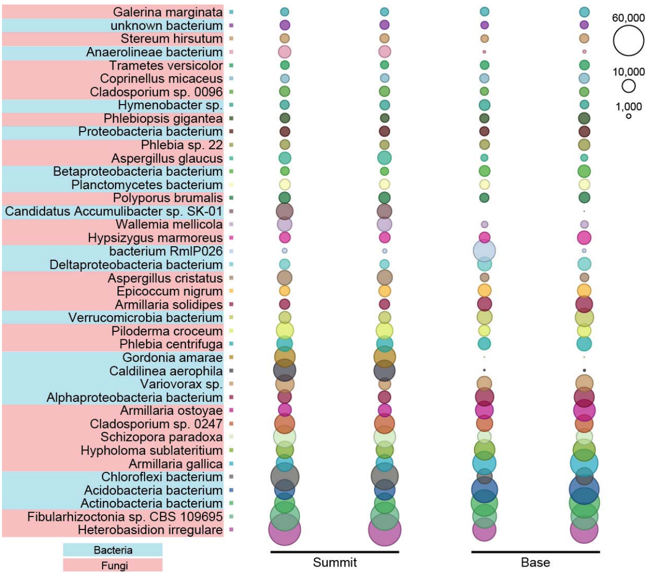

Figure S67. Top 40 most abundant species identified in the Mount Säntis air samples by the metagenomic analysis based on number of assigned sequencing reads. Bubble size is proportional to the number of sequencing reads assigned to each taxon.

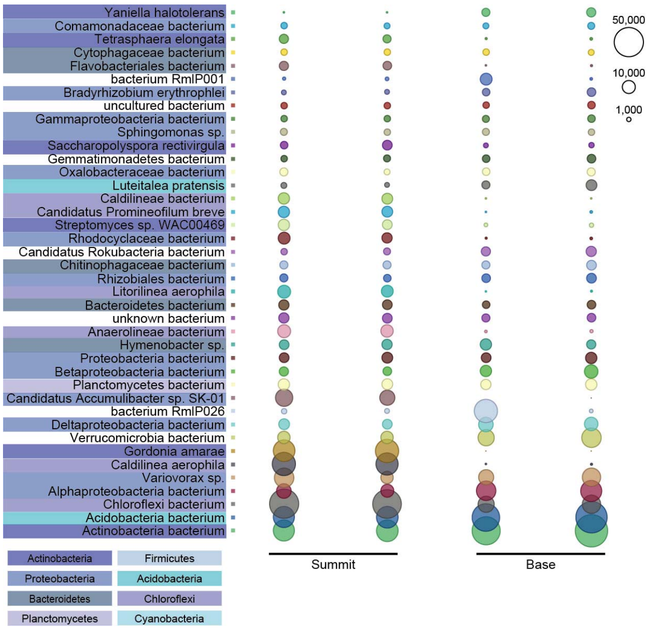

Figure S68. Top 40 most abundant bacterial species identified in the Mount Säntis air samples by the metagenomic analysis based on number of assigned sequencing reads. Bubble size is proportional to the number of sequencing reads assigned to each taxon.

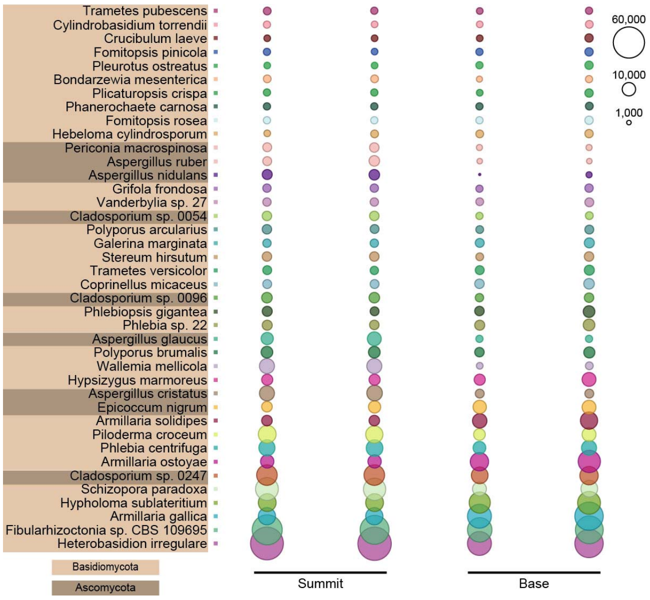

Figure S69. Top 40 most abundant fungal species identified in the Mount Säntis air samples by the metagenomic analysis based on number of assigned sequencing reads. Bubble size is proportional to the number of sequencing reads assigned to each taxon.

12) Vertical Air Sampling in the Built Environment (Residential High-rise Building)

Our very first vertical air sampling attempts were conducted on 4 Nov 2016 at the Pinnacle@Duxton residential housing complex in Singapore (Fig. S70). From 9:15-11:15am in the morning, air samples were simultaneously collected on open corridors of different floors at the Pinnacle@Duxton which is the tallest public housing (HDB) project in Singapore at a total height of 156m. Air samples were collected in triplicates at the ground floor (1.3m), Level 3 (~10m), Level 5 (~20m), Level 10 (~40m), Level 21 (~80m) and Level 49 (~140m). Replicates were later pooled to obtain sufficient DNA amounts for metagenomic sequencing.

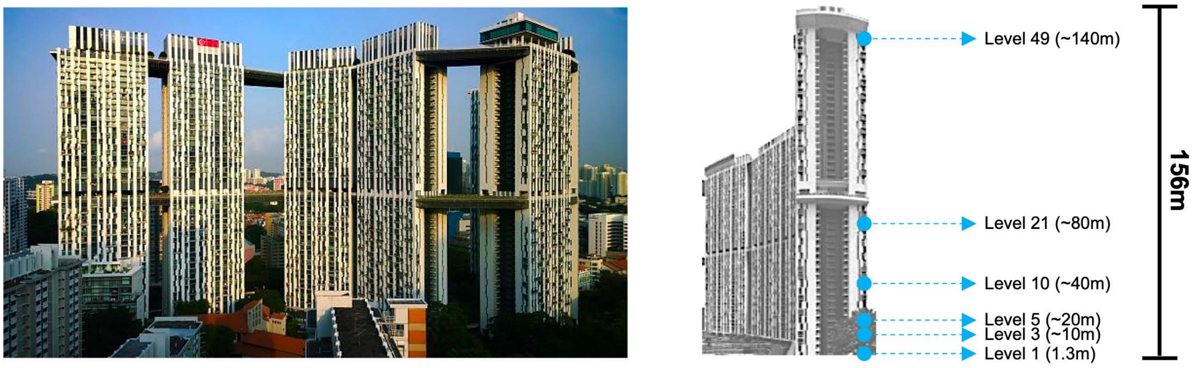

Figure S70. Vertical air sampling in the built environment. Overview of the sampling location at the Pinnacle@Duxton.

Metagenomic analysis did not reveal any significant differences in the microbial composition of the air samples collected at different levels of the building (Figures S71 – S74). As the sampling was conducted during the day when the surface of the planet heats up and causes ground-level air to rise up to higher altitudes, this most likely caused ground-level air to rise up to the highest sampling point and beyond, resulting in identical microbial composition of the air samples collected at the different levels of the building. In addition, solid, concrete structures such as this high-rise building may also alter the natural airflow of air masses near the ground. As such, we ruled out high-rise buildings as suitable sampling sites for future vertical air sampling experiments.

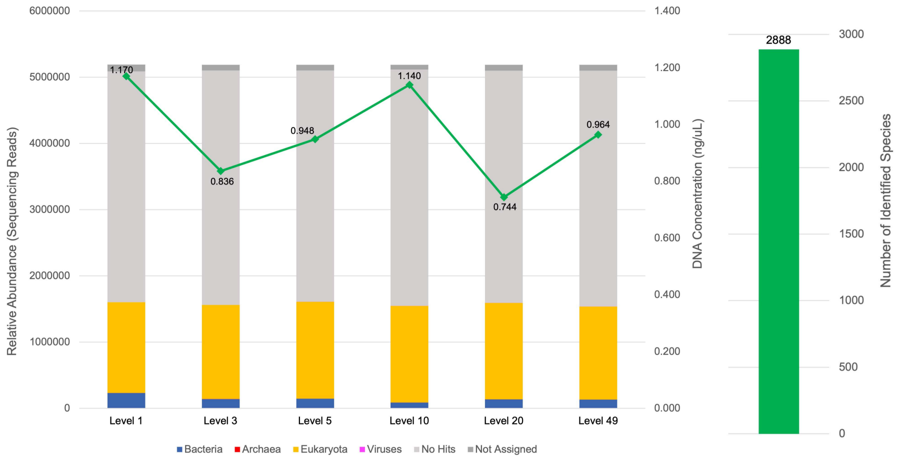

Figure S71. Taxonomic overview of relative abundances at superkingdom-level based on number of assigned sequencing reads for air samples collected at Pinnacle@Duxton. DNA concentrations of analyzed samples and total number of identified species (averages per time point and sampling location) are shown as well.

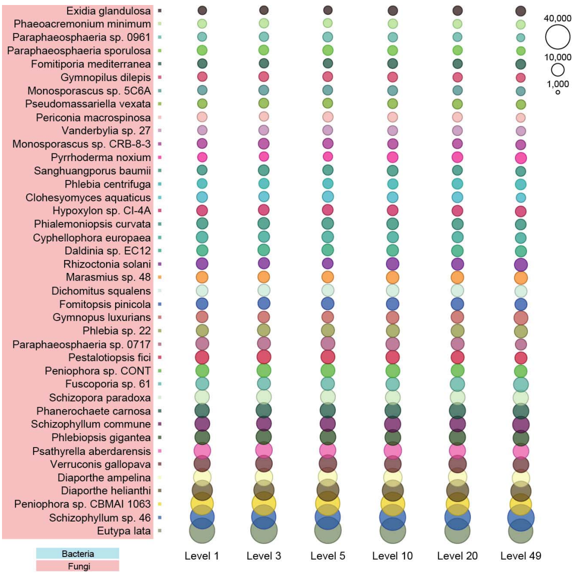

Figure S72. Top 40 most abundant species identified in the Pinnacle@Duxton air samples by the metagenomic analysis based on number of assigned sequencing reads. Bubble size is proportional to the number of sequencing reads assigned to each taxon.

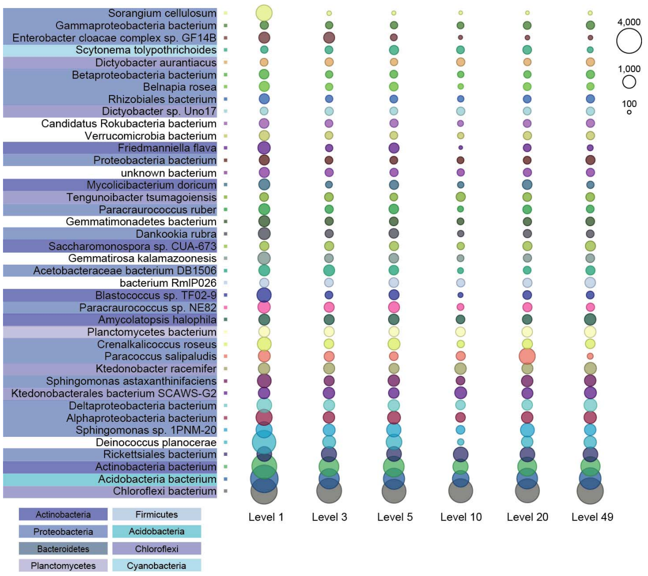

Figure S73. Top 40 most abundant bacterial species identified in the Pinnacle@Duxton air samples by the metagenomic analysis based on number of assigned sequencing reads. Bubble size is proportional to the number of sequencing reads assigned to each taxon.

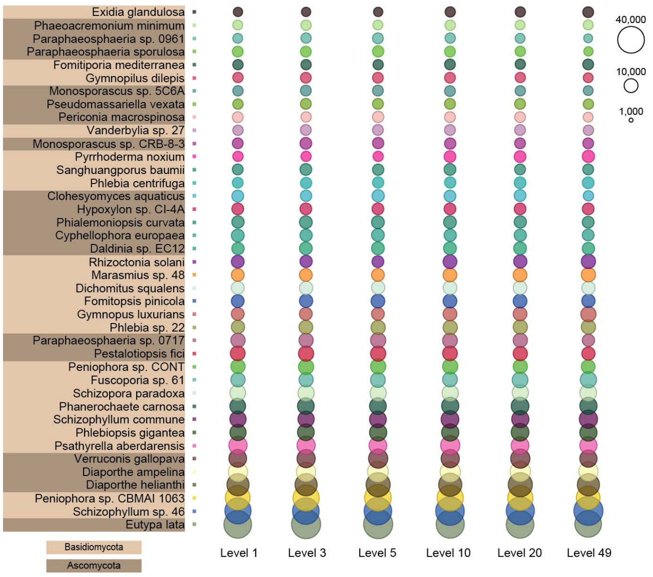

Figure S74. Top 40 most abundant fungal species identified in the Pinnacle@Duxton air samples by the metagenomic analysis based on number of assigned sequencing reads. Bubble size is proportional to the number of sequencing reads assigned to each taxon.

13) Sequencing statistics for additional samples presented in the supplement

Table S5. Overall statistics of total raw sequencing reads and remaining unmapped paired reads for Kaiju analysis after removal of human and human-associated microbial contamination for all additional samples presented here in the supplement. VRTV: Samples collected at the Pinnacle@Duxton; APCH: Samples collected at Mount Säntis; APG57-APG62: Samples collected at the meteorological tower in February 2018; APG63-APG72: Samples collected at the meteorological tower in July 2018.

Sample ID	Raw Reads (R1+R2)	Trimmed Reads (R1+R2)	GRCh38 mapping %	Unmapped Singletons	Unmapped Paired Reads	Human Microbiome Mapping %	Unmapped Singletons	Unmapped Paired Reads	Reads Used for Kaiju
VRTV1009	10,994,786	10,913,076	0.14%	11,668	10,894,536	-	-	-	10,894,536
VRTV1010	13,219,760	13,124,666	0.09%	13,344	13,109,326	-	-	-	13,109,326
VRTV1011	10,472,280	10,393,722	0.12%	11,656	10,378,118	-	-	-	10,378,118
VRTV1012	11,654,050	11,565,940	0.12%	11,896	11,548,986	-	-	-	11,548,986
VRTV1013	12,279,772	12,197,086	0.18%	13,564	12,171,522	-	-	-	12,171,522
VRTV1014	11,014,620	10,940,284	0.14%	12,452	10,922,196	-	-	-	10,922,196
APCH1	14,943,510	14,871,846	0.39%	12,936	14,810,576	-	-	-	14,810,576
APCH2	12,947,934	12,824,316	1.18%	26,800	12,666,634	-	-	-	12,666,634
APCH3	14,664,878	14,643,152	1.06%	27,668	14,481,186	-	-	-	14,481,186
APCH4	14,596,734	14,571,210	1.69%	20,808	14,319,334	-	-	-	14,319,334
APG57	6,977,350	6,960,034	0.08%	2,676	6,953,728	-	-	-	6,953,728
APG58	7,187,630	7,171,628	0.04%	2,424	7,168,222	-	-	-	7,168,222
APG60	6,942,074	6,926,228	0.16%	6,416	6,913,206	-	-	-	6,913,206
APG61	7,420,886	7,403,286	0.13%	6,360	7,391,750	-	-	-	7,391,750
APG62	6,906,612	6,889,256	0.11%	3,724	6,880,862	-	-	-	6,880,862
APG63	4,560,716	4,550,418	0.09%	8,388	4,544,230	-	-	-	4,544,230
APG64	5,004,222	4,992,550	0.16%	9,544	4,982,008	-	-	-	4,982,008
APG65	5,224,908	5,212,694	0.10%	10,908	5,204,934	-	-	-	5,204,934
APG66	6,099,236	6,085,422	0.10%	12,588	6,076,274	-	-	-	6,076,274
APG67	6,475,852	6,461,876	0.15%	12,540	6,449,082	-	-	-	6,449,082
APG68	5,581,542	5,569,316	0.13%	10,476	5,559,212	-	-	-	5,559,212
APG69	6,018,112	6,005,484	5.83%	35,716	5,646,248	-	-	-	5,646,248
APG70	4,757,270	4,746,464	0.44%	8,404	4,723,634	-	-	-	4,723,634
APG71	5,711,124	5,699,084	0.19%	13,148	5,685,002	-	-	-	5,685,002
APG72	6,297,928	6,268,710	0.76%	10,816	6,218,252	-	-	-	6,218,252

14) Presence of Cyanobacteria in the meteorological tower (MT) and research aircraft (RA) datasets

Cyanobacteria are photoautotrophic microorganisms and the only oxygenic photosynthetic prokaryotes that can be found in a wide range of diverse and extreme environments (1). Screening of the MT and RA metagenomic datasets for the presence of this unique group of bacteria revealed that Cyanobacteria can be found at the ground as well as greater heights at both locations (Figs. S75 and S76). Interestingly, similar to radio-tolerant bacteria, cyanobacterial species are more abundant at the tower top (200m) and at heights of 300m and above in the aircraft experiment as compared to the respective ground samples. In addition, while Cyanobacteria can be found throughout the day at greater heights, their abundance diminishes during the night hours at ground level. Furthermore, approximately 5 times more sequencing reads are being assigned to the most abundant cyanobacterial species in the RA experiment as compared to the MT dataset (e.g., up to 20,000 reads per sample assigned to a cyanobacterial species in RA (up to 0.54% of the total reads of a sample) as compared to up to 4,000 reads in MT (up to 0.09% of the total reads of a sample).

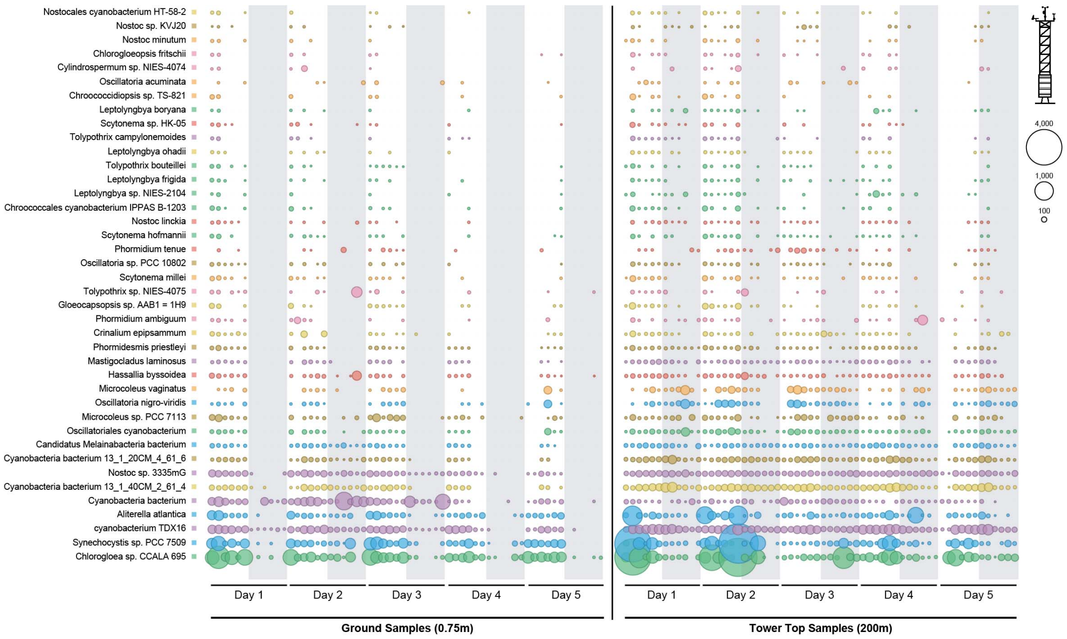

Figure S75. Top 40 Cyanobacteria in the meteorological tower (MT) dataset. Temporal (5 consecutive days) and vertical distribution (0-200m) of the top 40 most abundant cyanobacterial species based on number of assigned sequencing reads identified in air samples collected at the meteorological tower in October 2018. Ground samples grouped together and sorted by sampling time and day, followed by the 200m (tower top) samples. Samples collected during night hours are shaded in grey. Bubble sizes are proportional to the number of sequencing reads assigned to each taxon.

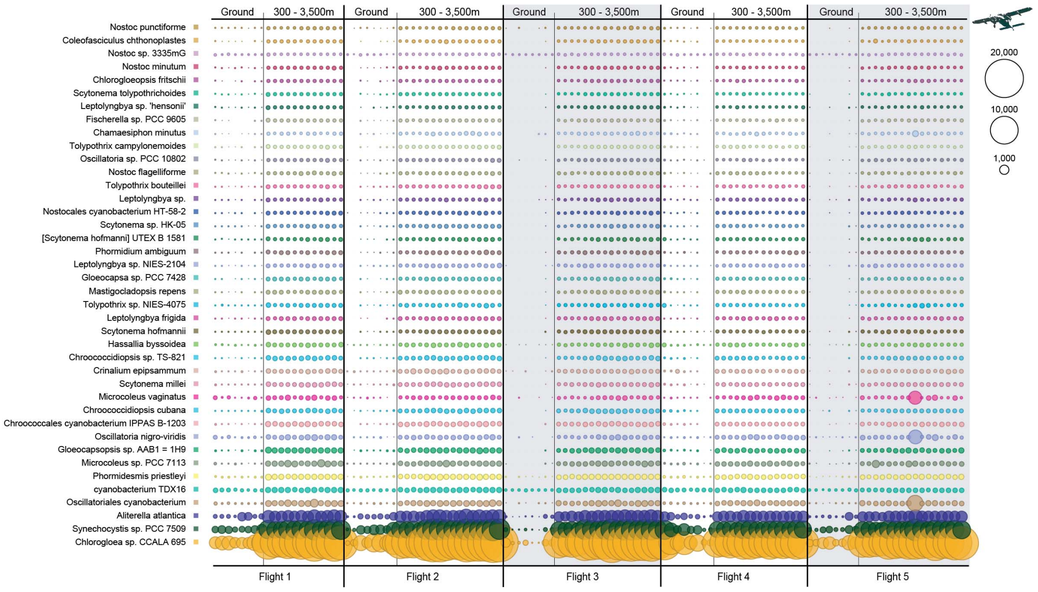

Figure S76. Top 40 Cyanobacteria in the research aircraft (RA) dataset. Ground samples followed by flight samples, grouped together by flight. Flight samples are ordered by altitude with the lowest altitude (300m) displayed first, followed by the higher altitudes. Ground samples are ordered according to their corresponding flight samples, e.g., ground samples that were taken simultaneously to the 300m flight samples are show first. Flights that were undertaken during night hours are shaded in grey. Bubble sizes are proportional to the number of sequencing reads assigned to each taxon.

15) Analysis into the presence of protists in the meteorological tower (MT) and research aircraft (RA) datasets

Protists are a diverse collection of eukaryotic organisms such as algae, amoebas and ciliates (e.g., paramecium). With some exceptions, protists are primarily microscopic in size, unicellular and form a paraphyletic assemblage that consists of diverse taxa with similar appearance (2). We screened the metagenomic datasets from the MT and RA experiments for the presence of protists (Rhodophyta, Glaucophyta, Stramenopiles, Apicomplexa, Ciliophora, Dinoflagella, Cercozoa, Foraminifera, Radiolaria, Euglenozoa, Percolozoa, Metamonada, Amoebozoa, Hemimastigophora, Apusozoa and Choanozoa). Some species belonging to the Stramenopiles clade (Fig. S77), Amoebozoa and Apicomplexa (MT dataset only, Fig. S78) were identified as shown in the following figures.

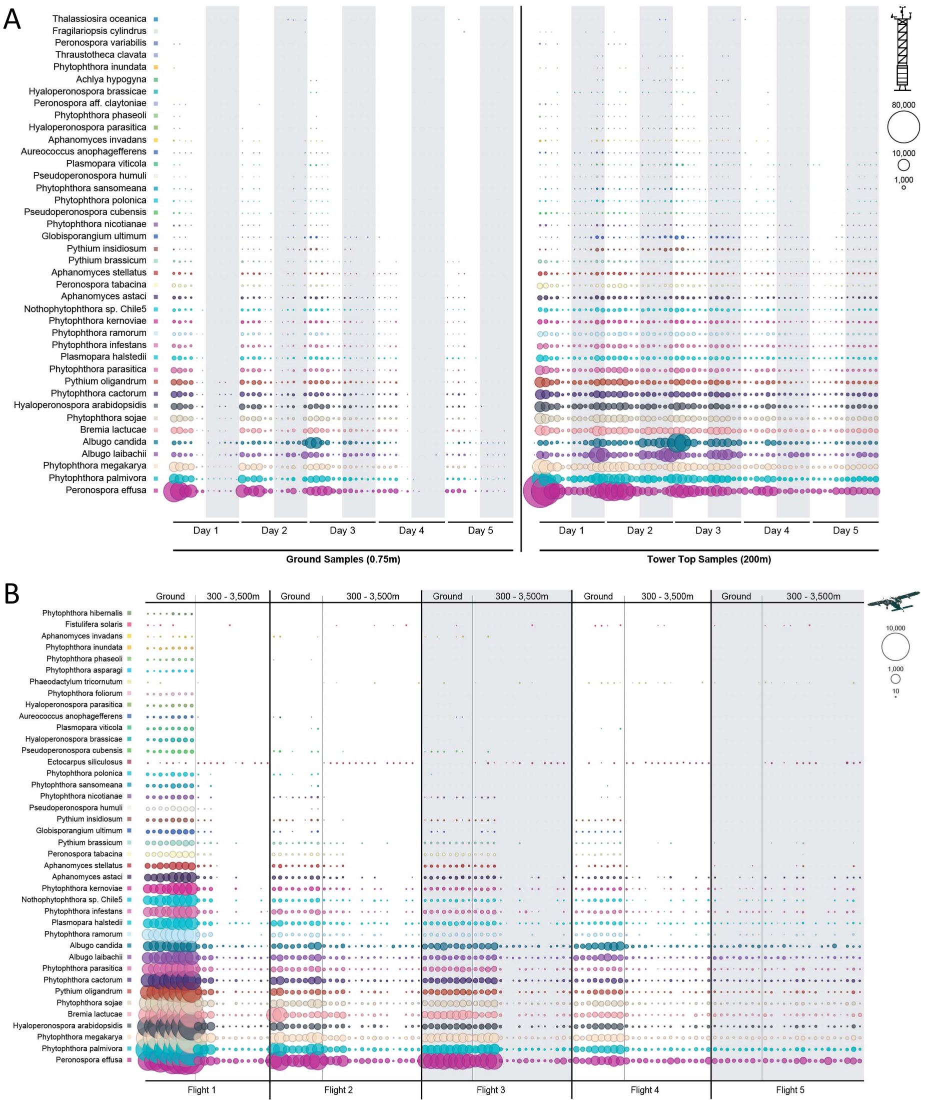

Figure S77. Top 40 Stramenopiles. A) Meteorological tower (MT) dataset. Temporal (5 consecutive days) and vertical distribution (0-200m) of the top 40 most abundant Stramenopiles species based on number of assigned sequencing reads identified in air samples collected at the meteorological tower in October 2018. Ground samples grouped together and sorted by sampling time and day, followed by the 200m (tower top) samples. B) Research aircraft (RA) dataset. Ground samples followed by flight samples, grouped together by flight. Flight samples are ordered by altitude with the lowest altitude (300m) displayed first, followed by the higher altitudes. Ground samples are ordered according to their corresponding flight samples, e.g., ground samples that were taken simultaneously to the 300m flight samples are shown first. Samples collected during night hours are shaded in grey. Bubble size is proportional to the number of sequencing reads assigned to each taxon.

At the meteorological tower, the identified Stramenopiles species appear to have slightly higher relative abundances at the tower top (200m) as compared to the ground. In addition, Stramenopiles are consistently present throughout the day at 200m whereas their abundances decrease at the ground during the nighttime hours (Fig. S77A). For the RA experiment, Stramenopiles were also observed at greater altitudes >300m throughout the day with little fluctuation in their relative abundance over time (Fig. S77B). In contrast to MT, however, the relative abundances of Stramenopiles species at the ground in RA was up to 100x higher than at greater altitudes of 300-3,500m. This may be caused by the different land types at the two sampling sites (e.g., MT = forested, RA = grass lands).

Protist species belonging to the Amoebozoa and Apicomplexa clades, identified only in the MT dataset:

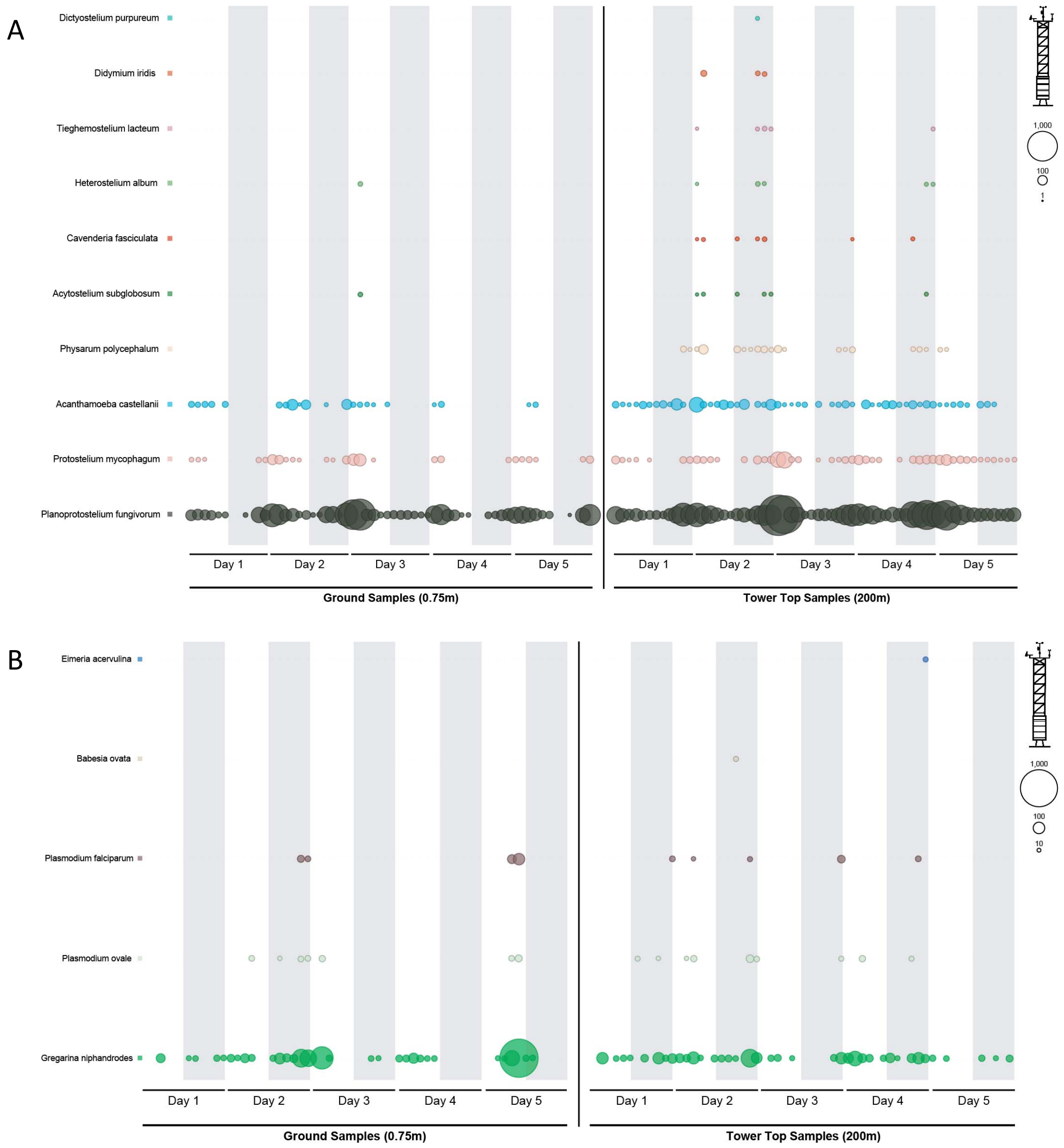

Figure S78. Other protists identified in the meteorological tower dataset (MT). Ground samples grouped together and sorted by sampling time and day, followed by the 200m (tower top) samples. Samples collected during night hours are shaded in grey. Bubble size is proportional to the number of sequencing reads assigned to each taxon. A) Amoebozoa. B) Apicomplexa.

16) <u>Day/night differences in abundance of cyanobacterial species and ground-associated bacteria at various altitudes</u>

Additional species-level analyses show that other airborne microbial taxa besides radio-tolerant bacterial species with specific physiological properties are also associated with either ground-or height-based settings (Fig. S79). Members of the cyanobacterial phylum (Figs. S79a, b), for example, which adhere to an oxygenic photoautotrophic lifestyle, show similar height distribution patterns as observed for radio-tolerant taxa, with greater abundance at altitudes >1,000m throughout the day and strong depletion below the mixing layer during the night-time hours. For ground-based taxa, which include members of the Actinobacteria phylum as well as other soil-associated microbiota, the dispersal pattern is reversed (Fig. S79c, d).

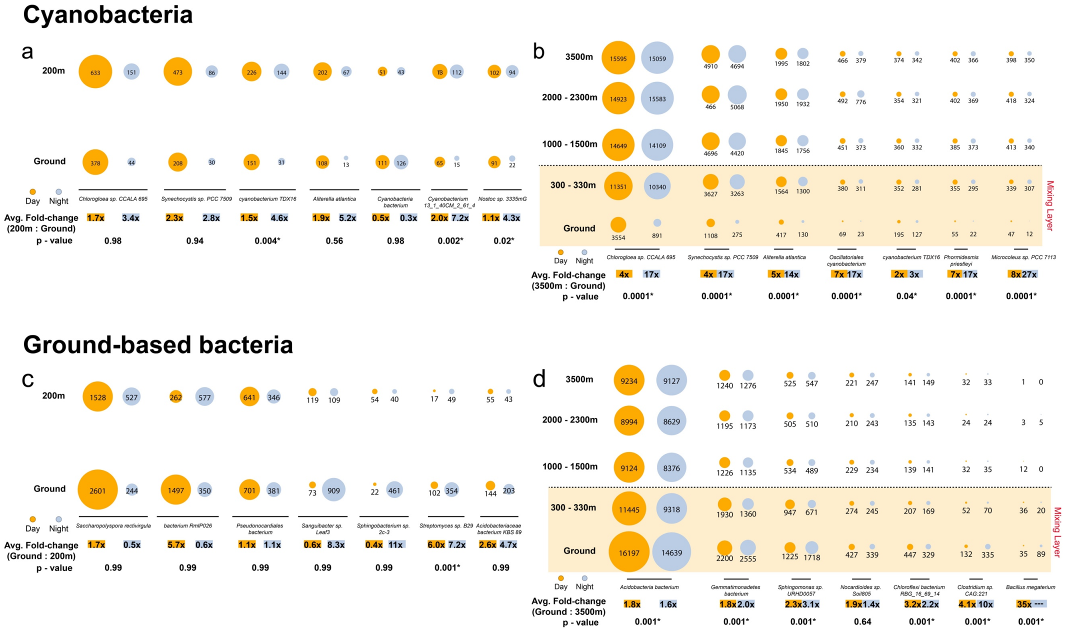

Figure S79. Day/night differences in abundance at various altitudes in the meteorological tower (MT) and research aircraft (RA) experiments. Bubble charts depicting the changes in relative proportion of the top 7 most abundant cyanobacterial species (a, b) as well as seven ground-associated bacteria (c, d) at different sampling times/altitudes in the MT and RA experiments, based on the number of assigned reads. Day abundances are colored in orange, while night abundances are highlighted in grey. The panels below the bubble charts shows the fold-change and the corresponding p-value (manyglm test) for the chosen taxa. a) Relative proportion of the 7 most abundant cyanobacterial species in MT at the ground and 200m height. b) Relative proportion of the 7 most abundant cyanobacterial species in RA at the ground and various heights. c) Relative proportion of the seven ground-associated bacterial species in MT at the ground and 200m height. b) Relative proportion of seven ground-associated bacterial species in RA at the ground and various heights.

17) <u>Abundance of UV repair genes</u>

The stratification of specific bacterial groups in the vertical air column is likely driven by a combination of their physiological capabilities and their resilience to various environmental stresses, such as desiccation, temperature, air pressure and radiation. Resistance to UV radiation is one such example.

Trimmed metagenomic reads were subjected to the humann3 pipeline with default parameters for functional analysis. Uniref90 database was used. Reads associated with the UvrABC system protein were then selected to assess the abundance of UV repair-related

genes at different air sampling heights (uvrA, uvrB, uvrC, phrB genes) in both experiments (MT and RA). The proportions of UV repair-related genes in our air samples increased with altitude for both, MT and RA experiments. On average, these genes were more prominent in the RA air samples as compared to the MT air samples (Fig. S80).

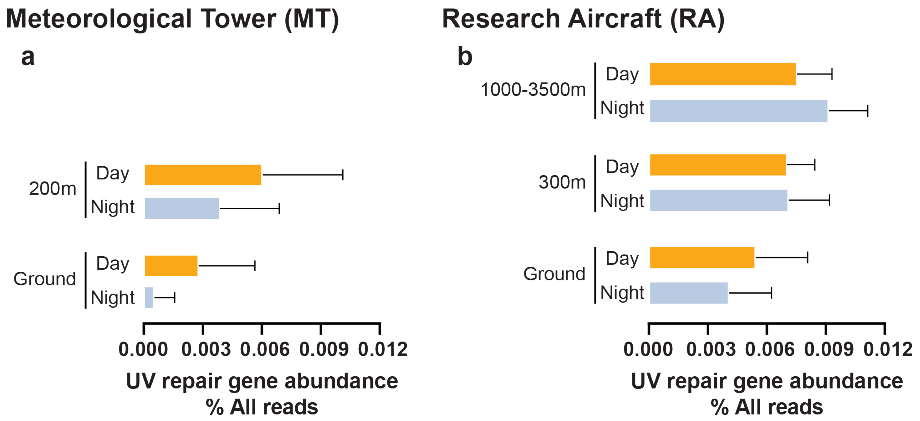

Figure S80. Abundance of UV repair genes. a) Meteorological tower (MT). b) Research aircraft (RA). The combined proportion of genes related to UV repair function. Daytime air samples are highlighted in orange, while night air samples are highlighted in grey. The error bars represent standard deviations among the air sample replicates.

18) <u>Supplementary References</u>

1. B.A. Whitton, *Ecology of Cyanobacteria II: Their Diversity in Space and Time*. Springer Science & Business Media (2012).
2. S. M. Adl *et al.*, The revised classification of eukaryotes. *J. Eukaryot. Microbiol*. 59, 429–493 (2012).
